# Geometry-Driven Control of PCL Fiber Deposition in a Hybrid Electrospinning–3D Printing Platform

**DOI:** 10.3390/polym18212662

**Published:** 2026-10-27

**Authors:** Manuel-Felipe Carranza-Montenegro, Dianney Clavijo-Grimaldo, Pedro-F Cárdenas

**Affiliations:** 1 School of Medicine, Universidad Nacional de Colombia, Bogotá, DC 111321, Colombia; dclavijog@gmail.com; 2 INPAC Group, School of Medicine, Fundación Universitaria Sanitas, Bogotá, DC 111321, Colombia; 3 School of Engineering, Universidad Nacional de Colombia, Bogotá, DC 111321, Colombia; pfcardenash@unal.edu.co

**Keywords:** polycaprolactone, electrospinning, programmed trajectory, XYZ motion, scanning electron microscopy, fiber morphology, tensile testing, reproducibility

## Abstract

The reproducibility of electrospun fibrous microarchitecture remains limited by the interaction between jet instability and deposition history. This study evaluated four programmed needle trajectories—Spiral, Static, Waves, and Zig-Zag—using a hybrid electrospinning–3D-printing motion platform. Four independent deposition runs were produced per trajectory (*n* = 4), yielding 16 SEM datasets and 16 tensile specimens. SEM images were calibrated automatically from the 50 µm scale bar and analyzed using a common physical ROI, skeleton-based fiber-diameter estimation, pore-opening analysis, spatial coverage, segment orientation, and 2D FFT descriptors. The four motion programs generated distinct and reproducible macroscopic deposition paths, while the replicated whole-ROI scalar descriptors showed substantial overlap: mean fiber diameter, pore opening, coverage, porosity, FFT alignment, and angular spread did not differ significantly among trajectories (one-way ANOVA *p* = 0.726–0.985 for the principal descriptors; Kruskal–Wallis *p* = 0.608–0.931). PCA likewise showed substantial overlap in the reduced global-descriptor space. Maximum tensile stress was 0.185 ± 0.052, 0.227 ± 0.079, 0.214 ± 0.147, and 0.189 ± 0.051 MPa for Spiral, Waves, Static, and Zig-Zag, respectively, without a significant inter-trajectory difference (*p* = 0.900). These results support a two-scale interpretation: programmed XYZ geometry provides deterministic control of the macroscopic deposition path and changes the spatial history of fiber landing and overlap, whereas fiber formation and whole-ROI averaged morphology remain strongly governed by electrohydrodynamic/material conditions and deposition-to-deposition variability. These findings demonstrate the feasibility of integrating electrospinning with programmable XYZ needle motion within a custom-designed platform. Although the programmed trajectories define distinct needle-motion geometries, no statistically significant differences were detected in the principal global morphological or mechanical descriptors under the investigated conditions.

## 1. Introduction

Reproducible control of electrospun fibrous architecture remains a central challenge in the development of polymeric membranes and scaffolds for biomedical applications. Structural characteristics such as fiber diameter, porosity, pore size distribution, fiber orientation, and spatial organization influence the mechanical behavior, transport properties, and biological performance of fibrous materials [1,2]. Electrospinning has attracted considerable interest because of its ability to produce continuous fibers with diameters ranging from the nanometer to the micrometer scale, generating highly porous structures with large specific surface areas and morphological features resembling components of the extracellular matrix [3,4,5]. Nevertheless, achieving reproducible control over the spatial distribution of electrospun fibers remains difficult, particularly when conventional electrospinning configurations are employed.

The electrospinning process is governed by complex interactions among solution properties, electrohydrodynamic forces, environmental conditions, and processing parameters. Polymer concentration, molecular weight, solvent composition, viscosity, electrical conductivity, applied voltage, flow rate, and working distance collectively influence jet formation, stretching, solvent evaporation, and fiber solidification [6,7,8,9]. In conventional far-field electrospinning, bending and whipping instabilities introduce substantial variability in the trajectory of the charged jet, limiting precise control over the position at which individual fibers reach the collector. Consequently, even under nominally constant operating conditions, differences in local fiber accumulation, overlapping, and orientation may arise. These characteristics complicate the fabrication of fibrous structures with predefined and reproducible microarchitectures [10,11,12].

Several strategies have been investigated to improve the spatial organization of electrospun fibers, including rotating collectors, patterned electrodes, modified electric-field configurations, and controlled relative displacement between the spinneret and collector [13,14,15]. These approaches can influence fiber alignment, deposition distribution, and the overall morphology of the resulting membranes. However, their effectiveness depends on the interplay between the imposed geometric or mechanical constraints and the electrohydrodynamic behavior of the polymer jet. Controlling the movement of the electrospinning needle does not necessarily imply equivalent control over the landing position or orientation of individual fibers. This distinction is essential when evaluating the extent to which programmed motion can be translated into reproducible changes in fibrous microarchitecture.

Three-dimensional (3D) printing has emerged as a complementary manufacturing technology because it enables the fabrication of structures with predefined geometries through computer-controlled positioning and material deposition. Conventional 3D-printing techniques provide considerable flexibility in the design of macroscopic architectures, including complex geometries and interconnected porous structures. However, their spatial resolution is frequently insufficient to reproduce the intricate organization of fibers at the nanoscale, which may limit applications requiring extracellular-matrix-like fibrous architectures. Specialized high-resolution 3D-printing systems can overcome some of these limitations, although their implementation may involve additional technical complexity, specialized equipment, and material-dependent processing constraints. Accordingly, integrating the geometric positioning capabilities associated with 3D-printing technologies with the fiber-forming capacity of electrospinning represents a promising strategy for developing manufacturing platforms that operate across different structural scales [16,17].

The integration of electrospinning and 3D printing has been investigated through several manufacturing strategies, particularly for the fabrication of multilayered and hierarchical scaffolds. In many reported approaches, the two techniques are implemented sequentially: a supporting structure is first fabricated by 3D printing and subsequently covered with electrospun fibers, or an electrospun membrane is produced before a 3D-printed structure is deposited onto its surface. Other configurations employ alternating fabrication steps to assemble composite structures with complementary architectural characteristics. Although these strategies have demonstrated the potential benefits of combining both technologies, they generally retain distinct fabrication stages and may require intermediate handling, sample repositioning, or alignment procedures. These operations can increase manufacturing complexity and introduce additional sources of variability [16,17,18].

An alternative approach involves integrating electrospinning directly with a programmable motion system derived from 3D-printing technology. In this configuration, the 3D-printer positioning mechanism provides controlled displacement of the electrospinning needle, while fiber generation and deposition remain governed by the electrohydrodynamic process. Unlike conventional sequential combinations, this arrangement allows programmed needle motion and electrospinning to operate simultaneously within a single experimental platform, without requiring material extrusion through a 3D-printing nozzle. The resulting configuration offers an opportunity to investigate how predefined needle trajectories influence the spatial history of fiber deposition and the characteristics of the resulting fibrous membranes. However, the relationship between programmed trajectory geometry and final fiber microarchitecture cannot be assumed to be deterministic, because jet instability, fiber–fiber interactions, and deposition variability remain operative throughout the process [19,20,21,22,23].

Poly(ε-caprolactone) (PCL) is a widely investigated biodegradable polyester for the fabrication of electrospun membranes and scaffolds because of its processability, relatively slow degradation, and mechanical characteristics suitable for a range of biomedical applications [24,25,26,27]. Its ability to form continuous fibers under appropriate electrospinning conditions makes it a useful model polymer for examining the relationship between processing variables and fibrous architecture. When the polymer formulation and electrospinning conditions are maintained constantly, variations in programmed needle movement can be investigated as an independent experimental factor. Such an approach provides a means of assessing whether changes in the geometric history of deposition are accompanied by measurable differences in fiber morphology, porosity, orientation, or mechanical response.

In this context, the present study investigates the influence of four programmed needle trajectories—Spiral, Static, Waves, and Zig-Zag—on the morphology and mechanical behavior of electrospun PCL membranes fabricated using a custom-designed hybrid electrospinning–3D-printing motion platform. The system integrates electrospinning with programmable XYZ positioning, enabling fiber deposition while the needle follows predefined motion trajectories. The experimental design maintains the polymer formulation and principal electrospinning parameters constantly isolating the influence of programmed needle movement. Fiber diameter, pore characteristics, spatial coverage, porosity, orientation-related descriptors, and tensile behavior are evaluated using scanning electron microscopy, quantitative image analysis, and mechanical testing. Attention is given to distinguishing the deterministic geometry of the programmed needle movement from the potentially stochastic organization of the deposited fibers. Rather than if different trajectories necessarily produce distinct microarchitectures, the study examines whether trajectory-dependent differences can be detected reproducibly in the resulting morphological and mechanical properties. This approach contributes to evaluating the capabilities and limitations of integrating electrospinning with programmable motion systems for the fabrication of fibrous polymeric materials.

## 2. Materials and Methods

### 2.1. Materials

Poly(ε-caprolactone) (PCL; CAS 134490-19-0; average Mn = 80,000 g/mol), chloroform (99.5%; CAS 67-66-3), and isopropyl alcohol (99.7%; CAS 67-63-0) were supplied by Sigma-Aldrich (St. Louis, MO, USA). The same commercial PCL material was used for all experimental groups. The polydispersity index (PDI) was not measured experimentally and was not available in the documentation used for this study; therefore, no PDI value is reported.

### 2.2. Solution Preparation

PCL was dissolved at 9% (*w*/*v*) in a 50:50 (*v*/*v*) chloroform/isopropyl alcohol mixture. The solution was stored at room temperature for 72 h and homogenized by ultrasonication using an Elmasonic 30H unit for 20 min at 21 °C. The same formulation and preparation procedure were used for every deposition. Viscosity and rheological flow curves were not measured during the experimental campaign; this omission is acknowledged as a limitation for full process reproducibility rather than replaced by literature-derived values.

### 2.3. Hybrid Electrospinning Platform and Operating Conditions

**Figure 1:**
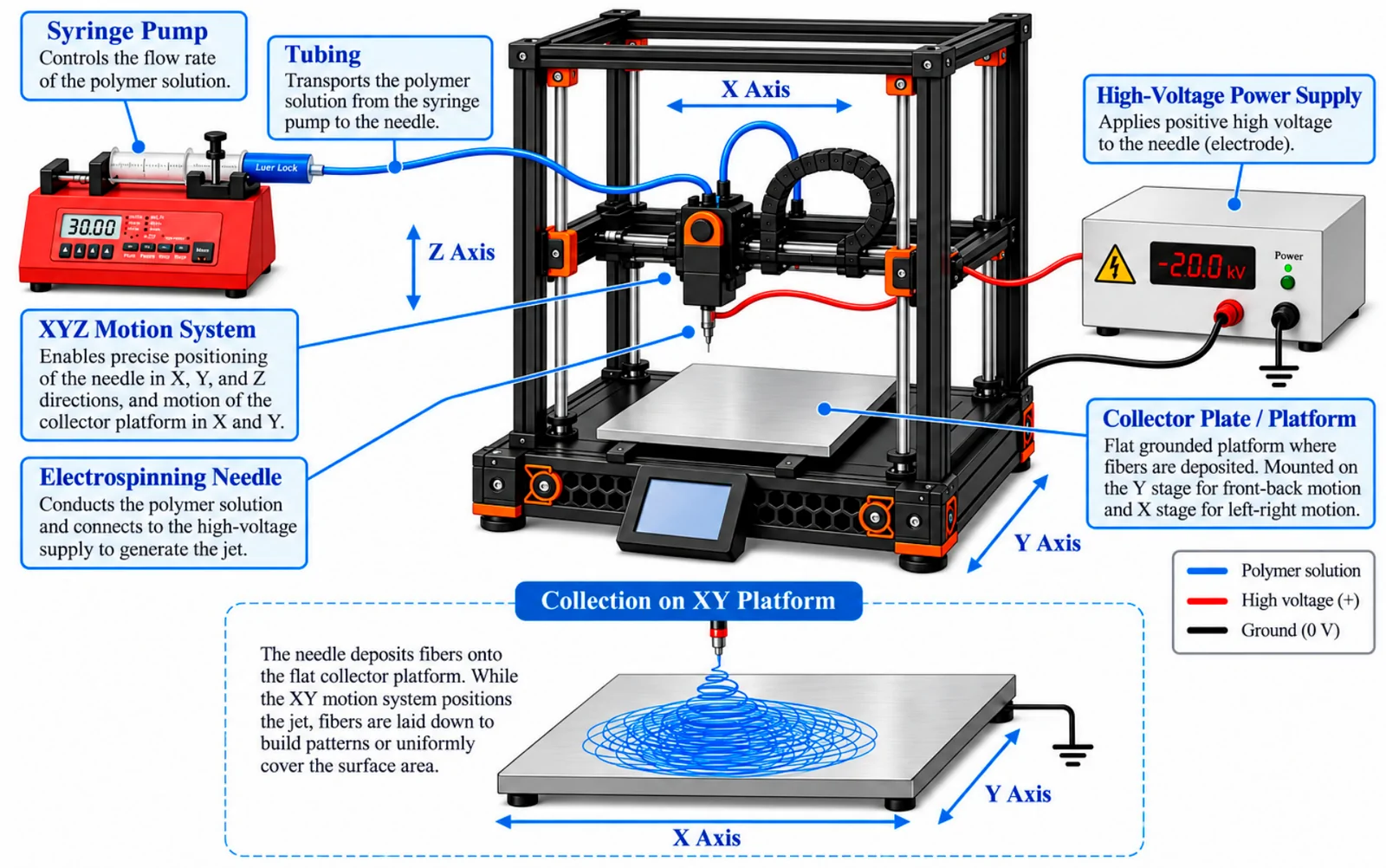
Hybrid electrospinning–3D-printing motion platform used for programmable needle displacement. The 3D-printing component provides XYZ positioning; fiber formation remains electrospinning-based.

**Figure 2:**
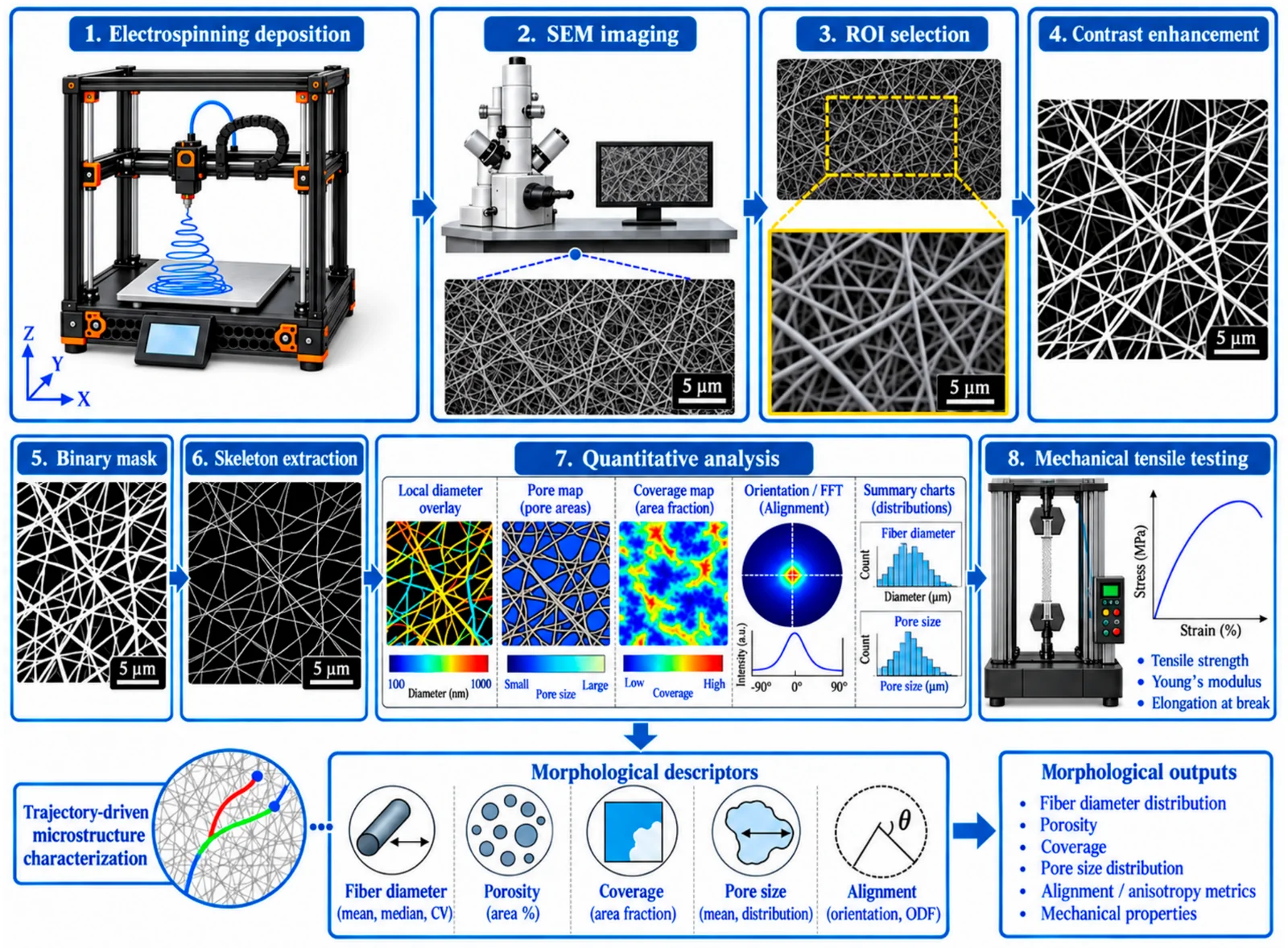
Experimental and analytical workflow used in the revised study, from programmed electrospinning deposition and SEM imaging to automated ROI-based morphology analysis and replicated tensile testing. The figure summarizes the image-processing sequence without duplicating the trajectory-specific result panels.

Fibrous mats were fabricated using a custom hybrid platform comprising a CZE1000R high-voltage power supply (Spellman High Voltage Corporation, Hauppauge, NY, USA), an NE-300 syringe pump (New Era Pump Systems, Inc., Farmingdale, NY, USA), a syringe–needle assembly, and a 3D-printer-derived XYZ motion system. The aluminum collector remained stationary while the needle assembly was displaced in the X–Y plane. Electrospinning was performed at 14 kV, a tip-to-collector distance of 14 cm, a flow rate of 1 mL/h, an in-plane nominal travel speed of 0.0025 m/s (2.5 mm/s), and a deposition time of 60 min. The Z position remained fixed during deposition. Room conditions during fabrication were 20 ± 2 °C and 55 ± 5% relative humidity. The platform configuration and the experimental analysis workflow are illustrated in Figure 1 and Figure 2, respectively.

### 2.4. Programmed Needle Trajectories

**Figure 3:**
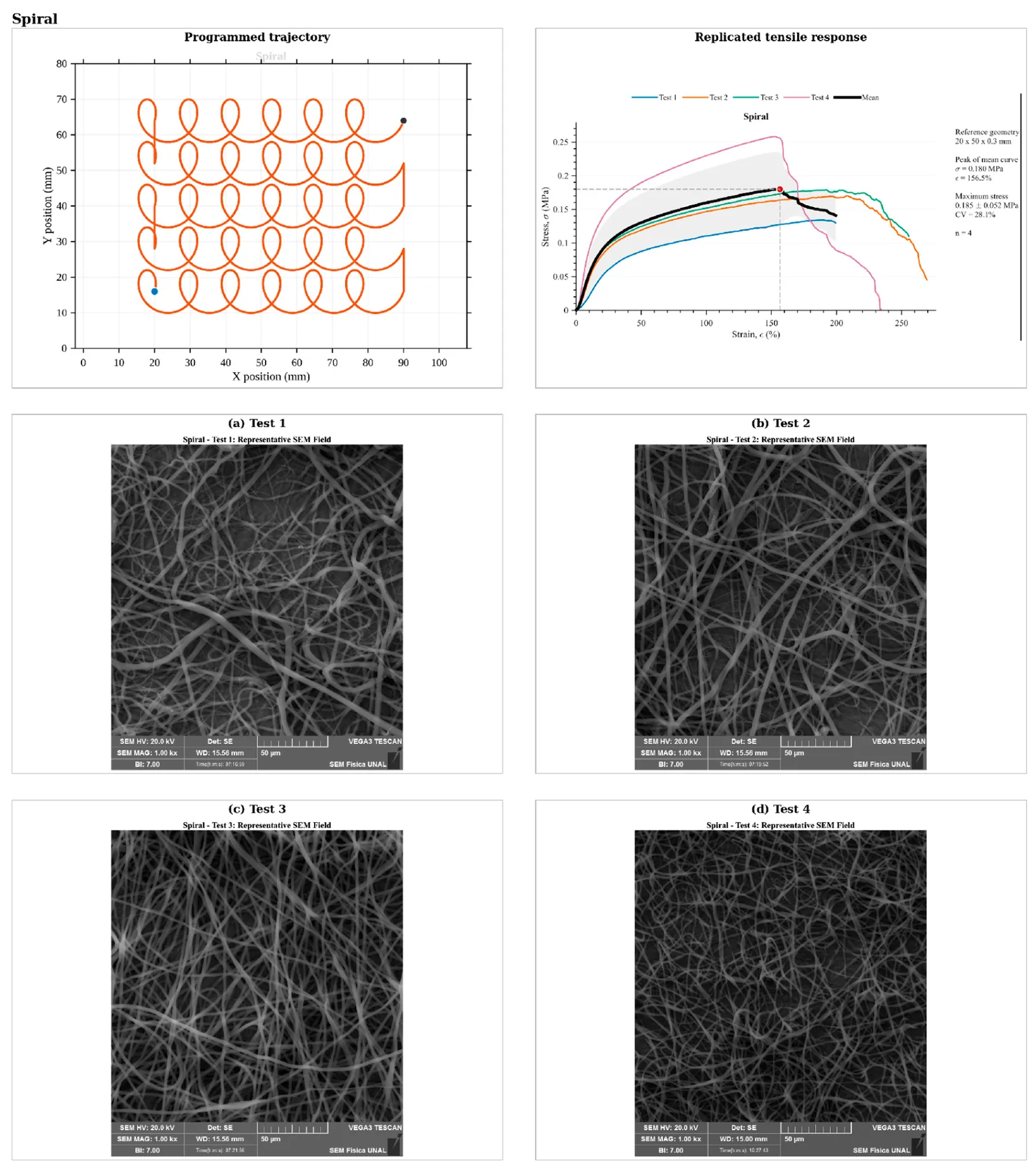
Spiral condition. The programmed trajectory and replicated tensile response are shown side by side at the top; the four independent SEM fields (T1–T4) are enlarged and arranged in a 2 × 2 matrix below. Each SEM field includes the original 50 µm scale bar.

**Figure 4:**
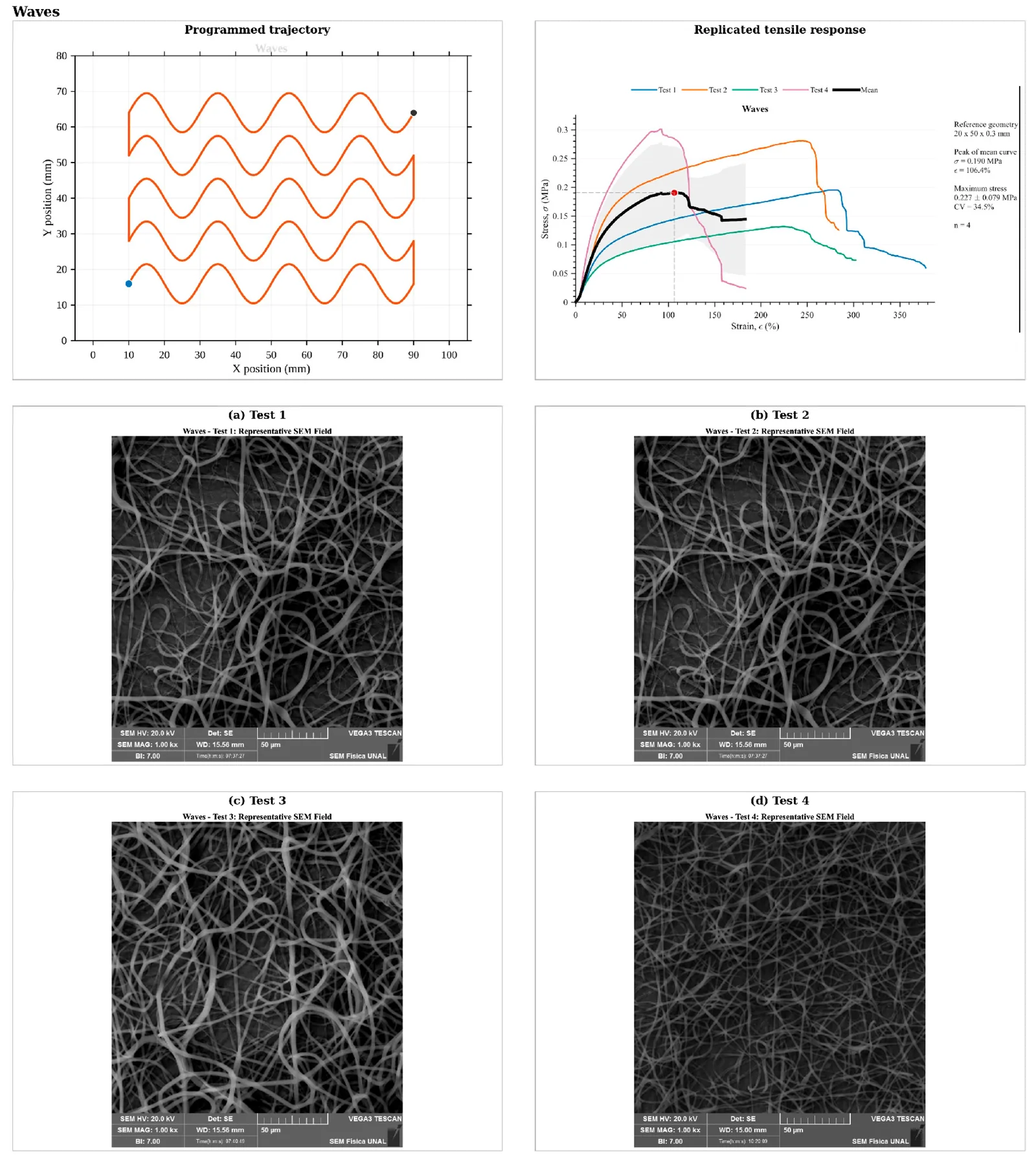
Waves condition. The programmed trajectory and four replicated tensile responses with their equal-weight mean are shown at the top; the four independent SEM fields (T1–T4) are enlarged in a 2 × 2 matrix below, following the same layout as Figure 3.

**Figure 5:**
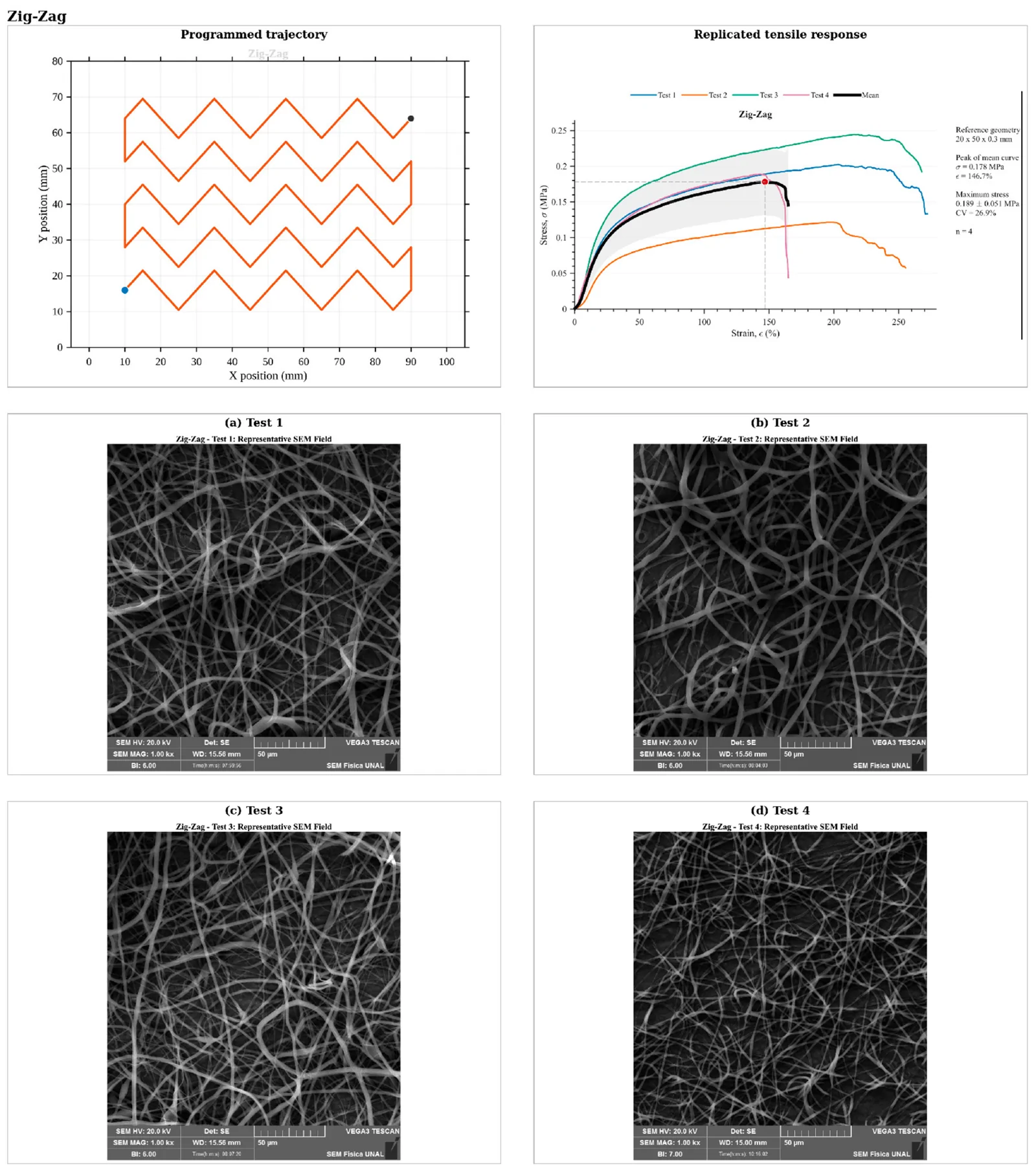
Zig-Zag condition. The triangular programmed path and four replicated tensile responses with their equal-weight mean are shown at the top; the four independent SEM fields (T1–T4) are enlarged in a 2 × 2 matrix below.

**Figure 6:**
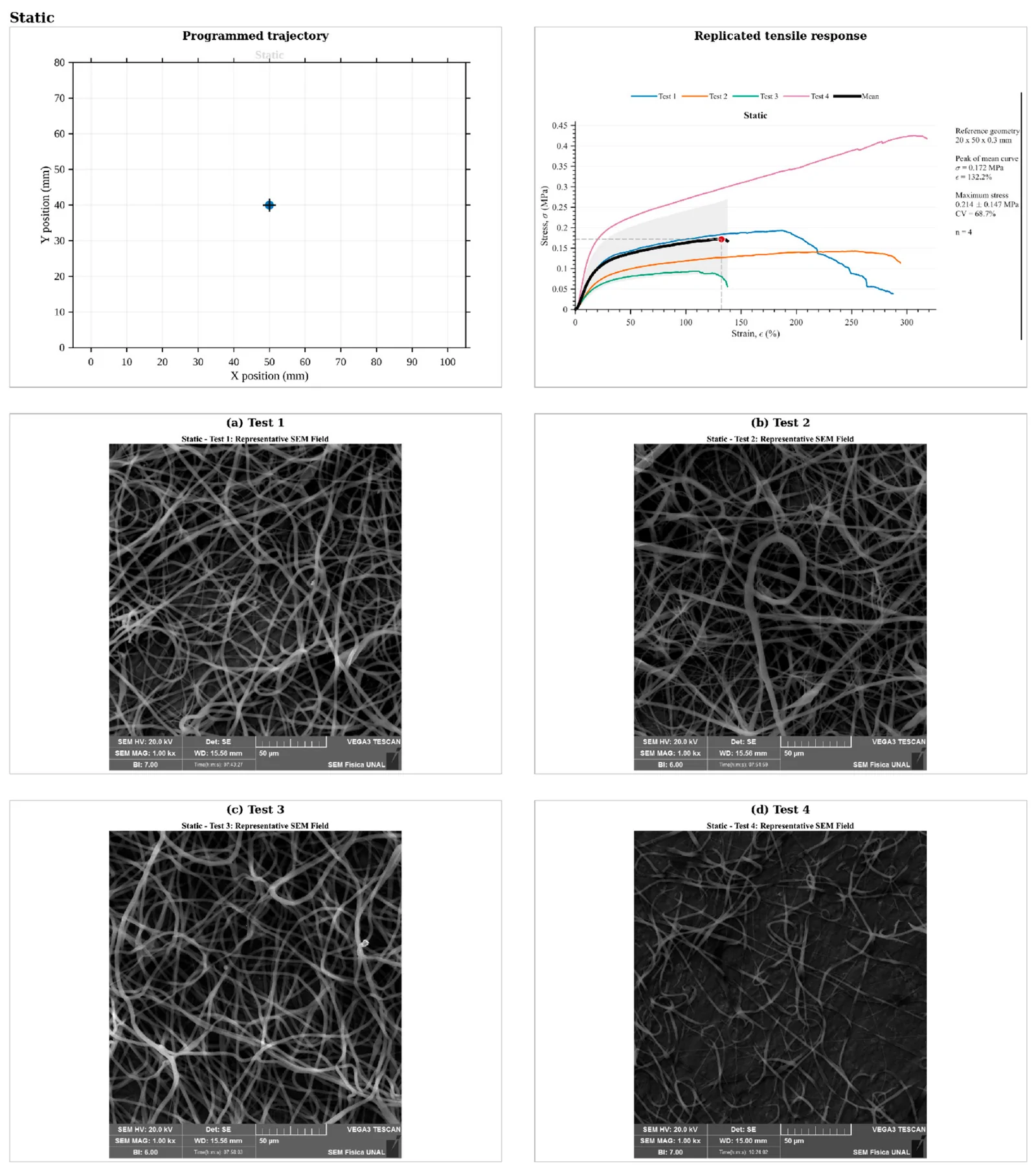
Static condition. The stationary programmed condition and four replicated tensile responses with their equal-weight mean are shown at the top; the four independent SEM fields (T1–T4) are enlarged in a 2 × 2 matrix below.

The four trajectories were reconstructed directly from the programmed trajectory files used in the experiment. All moving patterns were distributed over five horizontal bands with 12 mm center-to-center row spacing. The geometric parameters are summarized in Table 1. To avoid duplicating trajectory graphics, each programmed path is shown once, in the upper-left panel of its corresponding trajectory-specific result figure (Figure 3, Figure 4, Figure 5 and Figure 6). The Spiral program was a translating sequence of constant-size elliptical loops rather than a radially expanding Archimedean spiral; therefore, its initial and final loop radii were identical.

### 2.5. Experimental Replication

Four independent deposition runs were performed for each trajectory (*n* = 4 per group; 16 depositions total). Across those independent runs, four SEM fields and four tensile specimens were evaluated per trajectory. Replication was therefore defined at the deposition level. Local diameter points, pore-opening points, skeleton segments, spatial tiles, and FFT windows were treated as within-image measurements and were not counted as independent experimental replicates.

### 2.6. SEM Imaging

SEM imaging was performed with a VEGA3 scanning electron microscope (TESCAN, Brno, Czech Republic) using the secondary-electron detector. The images used for quantitative analysis were acquired at 20.0 kV and 1.00 × 10^3^ magnification with a 50 µm scale bar; the representative records show a working distance of approximately 15.5 mm. Four independent SEM fields were analyzed for each trajectory, for a total of 16 images.

### 2.7. Automated Calibration, ROI Selection, and Morphological Analysis

Image processing, quantitative morphological analysis, statistical calculations, and figure generation were performed using MATLAB R2025b, Update 7 (Version 25.2.0.3360999; The MathWorks, Inc., Natick, MA, USA), under Academic License No. 40830014 provided by the university. The image footer and 50 µm scale bar were detected automatically; the detected scale length was 187 pixels for all 16 images, corresponding to 0.267380 µm/pixel. A common physical ROI of approximately 183.02 × 182.77 µm (33,449.9 µm^2^) was then extracted while excluding the instrument footer and borders. Automatic detection outputs were visually quality checked before quantitative analysis. However, the automated segmentation and morphological measurements were not independently validated against manual measurements or an alternative image-analysis method. Consequently, the reported morphological descriptors represent estimates obtained using the specified image-processing workflow.

The ROI was converted to grayscale and processed using contrast-limited adaptive histogram equalization (8 × 8 tiles; clip limit 0.01), Gaussian smoothing (σ = 0.8 pixel; 0.214 µm), white top-hat background correction (10-pixel radius; 2.674 µm), and combined adaptive/global fiber-enhancement thresholds. Morphological cleaning, closing, bridging, majority filtering, skeletonization, and branch/end-point detection were applied to obtain the final fiber network.

Local fiber diameter was estimated from the Euclidean distance transform sampled on the skeleton after excluding a two-pixel neighborhood around branch and end points. At the calibrated scale, the exclusion radius was 0.535 µm. Diameter outliers were filtered with the 1.5 × IQR rule. Coverage was defined as segmented fiber-area fraction and porosity as 1 − coverage. Pore opening was estimated from regional maxima of the void-distance map. Segment-based orientation and a complementary 2D FFT workflow were used to calculate dominant directions, alignment indices, angular spread, orientation entropy, and spatial alignment maps [11,12]. A 4 × 4 within-image grid was additionally used to quantify spatial variability, but these tiles were not treated as independent replicates.

Across all 16 images, the workflow generated 401,092 local diameter measurements, 70,824 pore-opening measurements, 20,690 accepted skeleton orientation segments, and 14,400 local FFT windows. These measurement counts describe within-image sampling density; trajectory-level statistical tests used *n* = 4 independent depositions per group.

### 2.8. Tensile Testing

Four tensile specimens were tested per trajectory (16 specimens total). Rectangular specimens were prepared using a common reference geometry of 20 × 50 × 0.3 mm and were held with flat grips. Testing was performed on a Shimadzu Autograph AG-IS universal testing machine equipped with a 5 kN load cell under stroke control at 50 mm/min. The recorded laboratory conditions were 16.5 °C and 59% relative humidity; these values describe ambient conditions at the time of testing and were not actively imposed or controlled.

The original test setup was informed by ASTM D638; however, ASTM D638 is intended for standard plastic tensile specimens and explicitly identifies ASTM D882 as the preferred method for thin sheeting below 1 mm. Because the present fibrous mats had a nominal thickness of 0.3 mm and rectangular strip geometry, the results are reported as comparative tensile responses rather than as formal D638-compliant material properties [28,29]. The 5 kN load cell also provides much greater capacity than required for the measured forces, which is acknowledged as a sensitivity limitation. Future standardized testing should use a lower-capacity load cell and a protocol aligned with D882 or another validated method for nonwoven fibrous mats.

Elastic modulus was not reported because deformation was inferred from crosshead/grip separation without an extensometer and because grip compliance, possible slippage, and uncertainty in the effective load-bearing cross-section of porous fibrous mats would make a conventional modulus value difficult to interpret. Basis weight (GSM) was not measured during the campaign, so equal GSM among groups is not claimed. Mechanical interpretation is therefore restricted to the tested geometry and the reported replicated stress–strain responses.

### 2.9. Statistical Analysis

Image-level and specimen-level results are reported as mean ± standard deviation (SD) across the four independent depositions/specimens in each trajectory group, together with the coefficient of variation (CV) where informative. Between-trajectory comparisons used one-way ANOVA as a parametric analysis and Kruskal–Wallis testing as a distribution-free sensitivity analysis. The significance level was α = 0.05. Because none of the omnibus comparisons of the principal SEM descriptors or maximum tensile stress reached *p* < 0.05, post hoc pairwise significance testing was not interpreted. Effect magnitude was summarized with η^2^. Exploratory multivariate analysis was performed by standardizing a nonredundant descriptor set and applying principal-component analysis (PCA), centroid distances, and silhouette analysis. Within-image pixel- or segment-level measurements were never substituted for independent deposition replicates. Given the limited sample size (*n* = 4 per trajectory), nonsignificant results were not interpreted as evidence of equivalence among experimental groups.

## 3. Results

### 3.1. Replicate Consistency

Replication substantially changed the interpretation relative to the original single-sample comparison. Mean fiber diameter showed between-deposition CV values of 13.6–18.7% across the four trajectories, whereas mean pore opening was more reproducible (CV 3.1–8.3%). Coverage variability ranged from 2.7% for Spiral to 13.0% for Static. In contrast, FFT alignment index was less reproducible, with between-deposition CV values of 23.9–65.6%, indicating that directional organization was especially sensitive to local stochasticity and deposition-to-deposition variation. The four full SEM fields for each trajectory are shown once in the trajectory-specific composite Figure 3, Figure 4, Figure 5 and Figure 6. To avoid duplicate figures, the complete replicate-resolved intermediate image-processing and quantitative outputs are provided only in Appendix A (Figure A1, Figure A2, Figure A3, Figure A4, Figure A5, Figure A6, Figure A7, Figure A8, Figure A9, Figure A10, Figure A11, Figure A12, Figure A13, Figure A14, Figure A15, Figure A16, Figure A17, Figure A18, Figure A19, Figure A20, Figure A21, Figure A22, Figure A23, Figure A24, Figure A25, Figure A26, Figure A27, Figure A28, Figure A29, Figure A30, Figure A31, Figure A32, Figure A33, Figure A34, Figure A35, Figure A36, Figure A37, Figure A38, Figure A39, Figure A40, Figure A41, Figure A42, Figure A43, Figure A44, Figure A45, Figure A46, Figure A47, Figure A48, Figure A49, Figure A50, Figure A51, Figure A52 and Figure A53).

### 3.2. Spiral

Across the four independent Spiral depositions, mean fiber diameter was 1.736 ± 0.235 µm, mean pore opening was 1.984 ± 0.073 µm, coverage was 0.560 ± 0.015, and porosity was 0.440 ± 0.015. The low coverage CV (2.7%) and pore-opening CV (3.7%) indicate good internal reproducibility for compactness-related descriptors. FFT alignment was more variable (0.138 ± 0.090), consistent with the visually stochastic orientation of individual fibers despite the regular macroscopic looped path.

The maximum tensile stress across Spiral specimens was 0.185 ± 0.052 MPa (CV 28.1%). The equal-weight mean stress–strain curve reached a peak of approximately 0.180 MPa at 156.5% strain. These results show reproducible gross deformation behavior with moderate between-specimen variability, but they do not by themselves establish a trajectory-specific mechanical advantage.

### 3.3. Waves

Waves produced a mean fiber diameter of 1.752 ± 0.252 µm, a mean pore opening of 1.994 ± 0.061 µm, coverage of 0.553 ± 0.020, and porosity of 0.447 ± 0.020. Compactness-related measures were internally consistent (coverage CV 3.6%; pore-opening CV 3.1%). The FFT alignment index was 0.114 ± 0.037 and remained more variable than pore or coverage descriptors.

Maximum tensile stress was 0.227 ± 0.079 MPa (CV 34.5%), the highest group mean among the four trajectories, although the large overlap among individual curves prevented a statistically significant inter-group difference. The mean curve peaked near 0.190 MPa at 106.4% strain.

### 3.4. Zig-Zag

Zig-Zag showed a mean fiber diameter of 1.719 ± 0.274 µm, a mean pore opening of 2.018 ± 0.168 µm, coverage of 0.544 ± 0.031, and porosity of 0.456 ± 0.031. The segment-based and FFT orientation descriptors did not show a reproducible strong alignment signature across the four independent depositions; the mean FFT alignment index was 0.095 ± 0.040 and the angular spread was 48.74 ± 1.05°.

Maximum tensile stress was 0.189 ± 0.051 MPa (CV 26.9%). The mean curve peaked near 0.178 MPa at 146.7% strain. The four curves again showed substantial overlap with those of the other programmed trajectories.

### 3.5. Static

Static yielded a mean fiber diameter of 1.791 ± 0.335 µm, mean pore opening of 2.028 ± 0.127 µm, coverage of 0.546 ± 0.071, and porosity of 0.454 ± 0.071. Static displayed the largest between-deposition variability in coverage and porosity among the four groups (coverage CV 13.0%; porosity CV 15.7%), illustrating that a stationary needle does not eliminate electrospinning stochasticity.

Maximum tensile stress was 0.214 ± 0.147 MPa, corresponding to a CV of 68.7%, the highest mechanical dispersion observed. One specimen reached markedly higher stress than the other three, which increased the group mean and SD. The mean curve peaked near 0.172 MPa at 132.2% strain.

### 3.6. Replicated Inter-Trajectory Morphological Comparison

**Figure 7:**
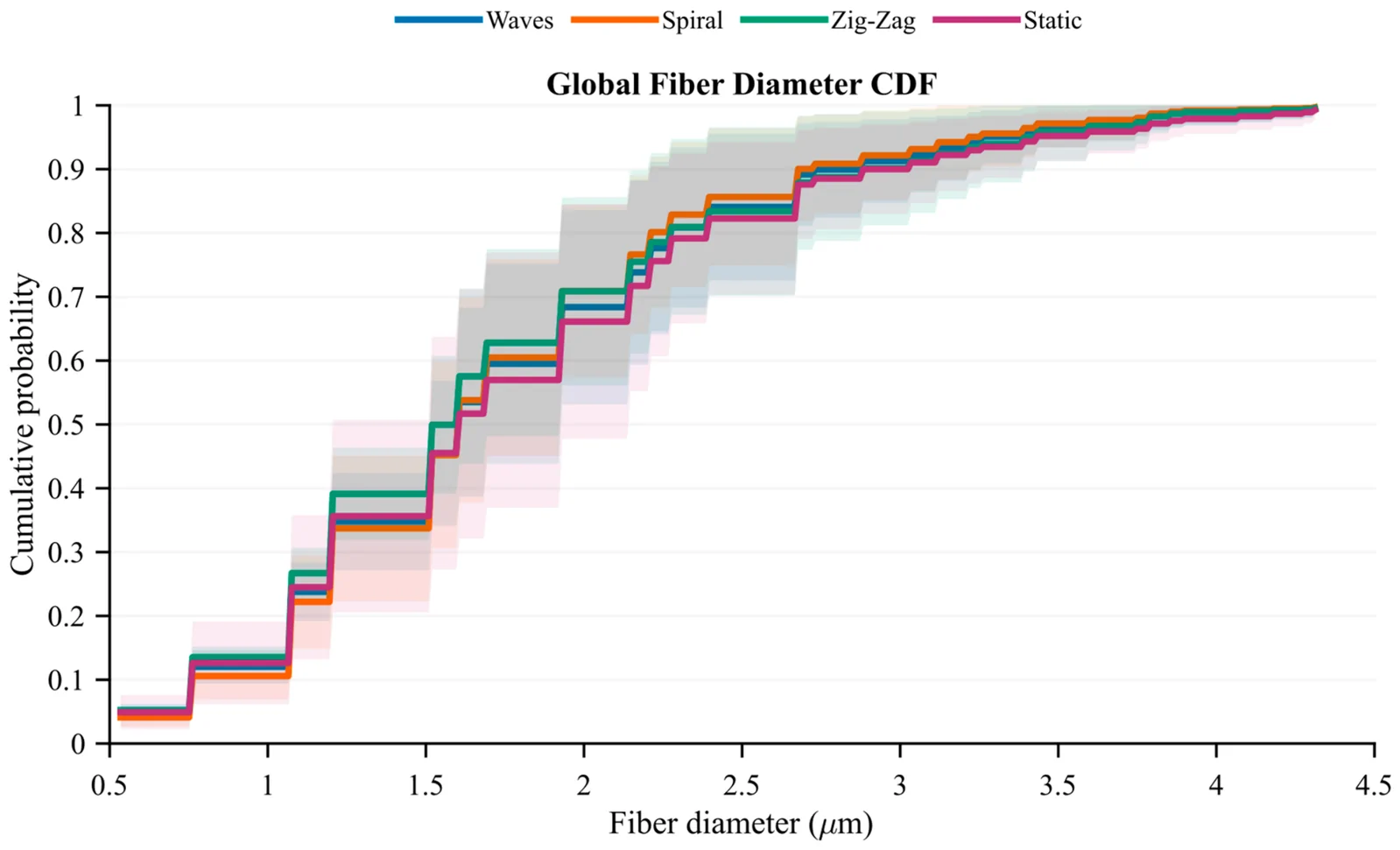
Equal-weight trajectory-level comparison of fiber-diameter distributions. The replicated image-level central values overlap substantially among groups.

**Figure 8:**
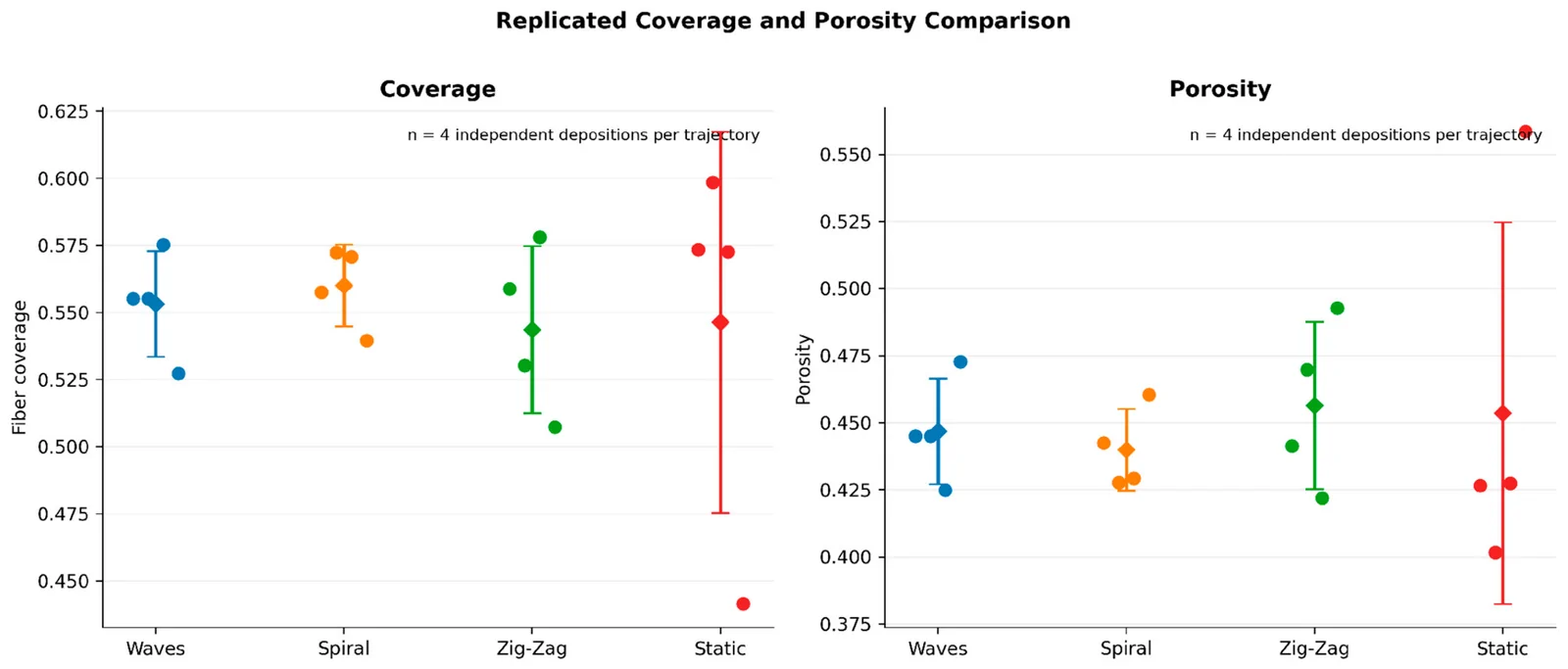
Replicated comparison of fiber coverage and porosity among trajectories. Error bars represent between-deposition variability. Blue, orange, green, and red denote Waves, Spiral, Zig-Zag, and Static, respectively.

**Figure 9:**
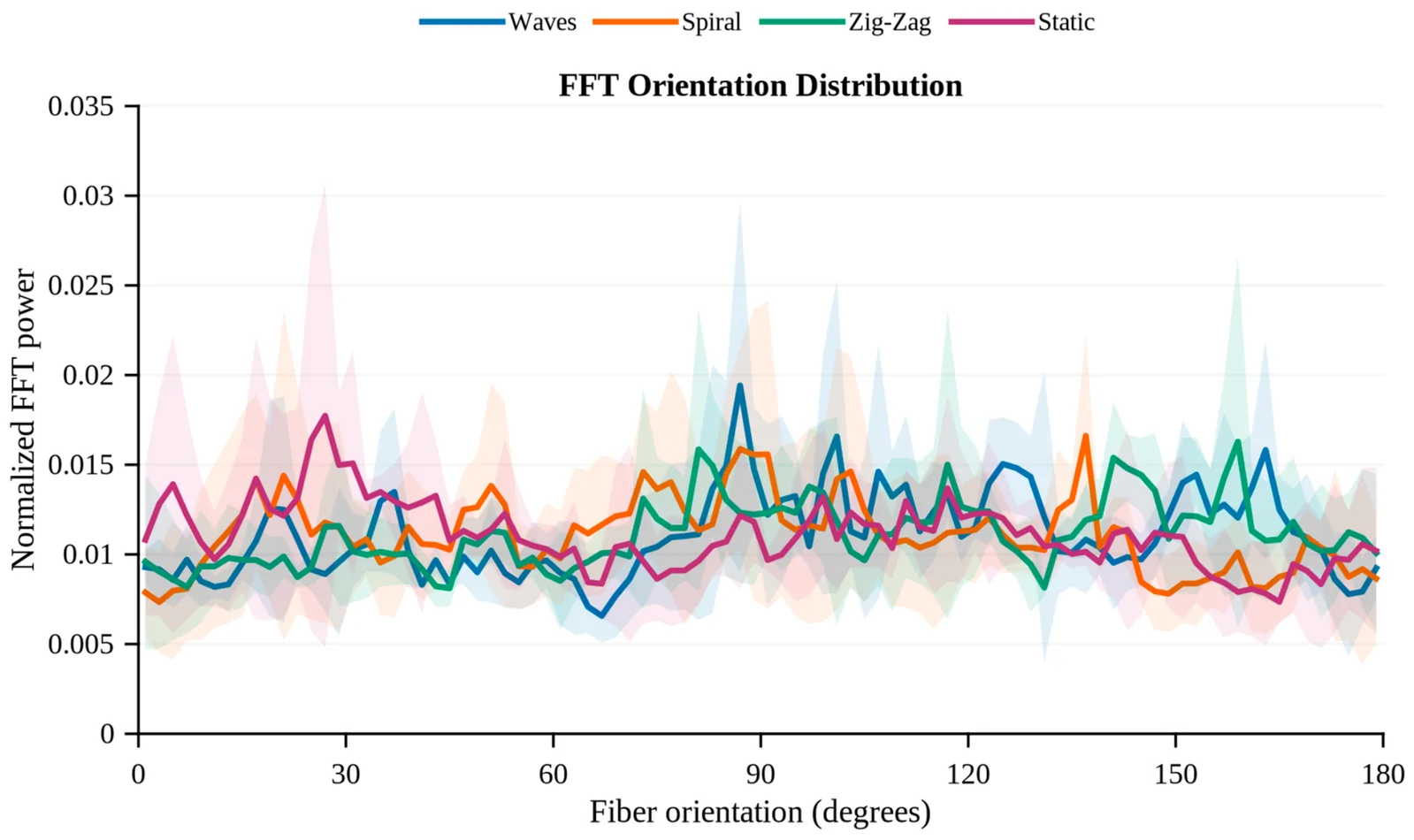
FFT-based orientation comparison across the replicated trajectory groups.

**Figure 10:**
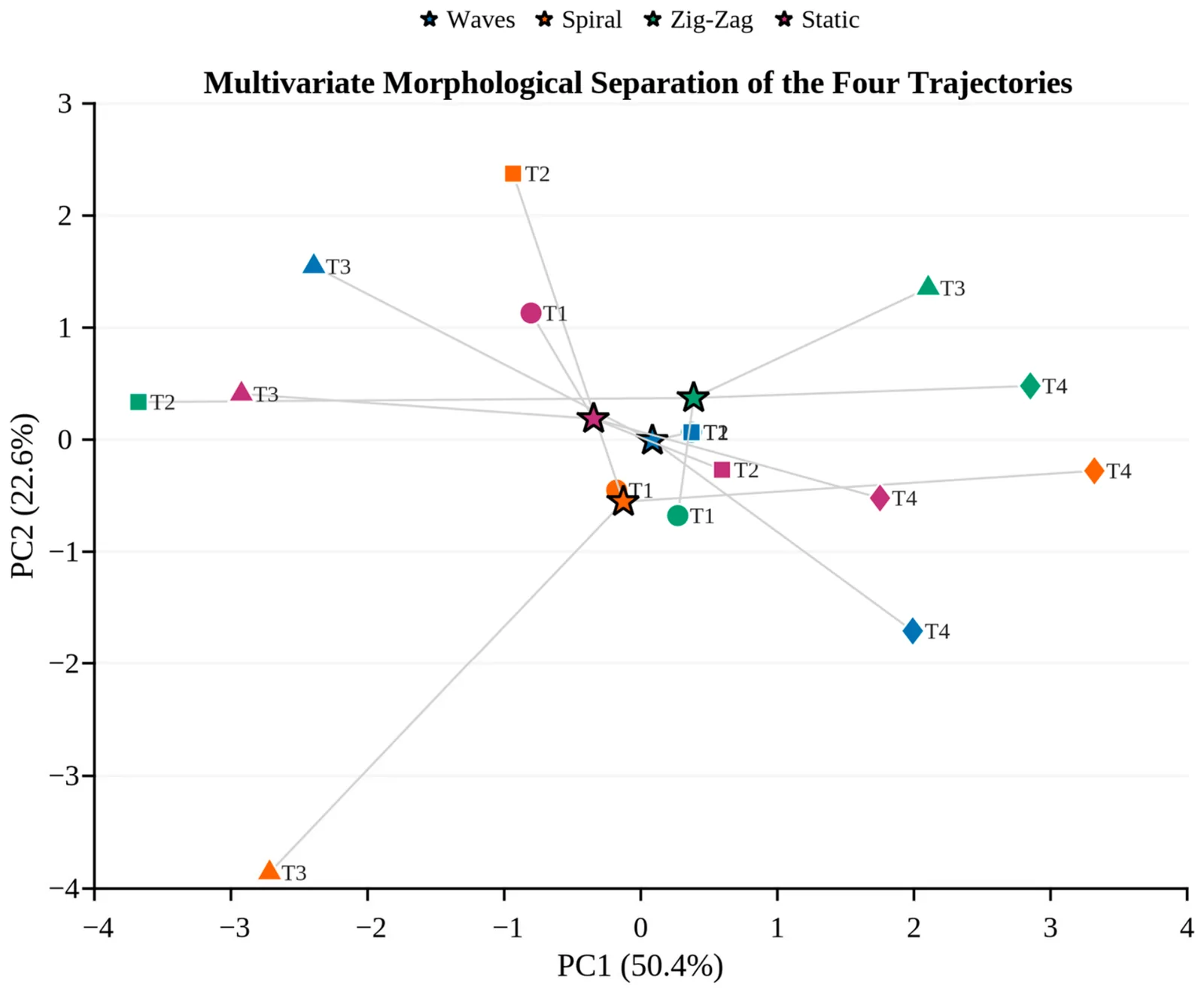
Exploratory PCA of nonredundant image-level descriptors. The first two components explained 73.0% of total standardized variance, but the trajectory groups overlapped substantially. Colors distinguish Waves (blue), Spiral (orange), Zig-Zag (green), and Static (purple); circles, squares, triangles, and diamonds denote independent tests T1, T2, T3, and T4, respectively; black-outlined stars mark the group centroids.

None of the principal whole-ROI SEM descriptors showed a statistically significant difference among the four trajectory groups. The smallest ANOVA *p*-value among the listed descriptors was 0.673 for segment alignment, and the largest η^2^ values were approximately 0.10–0.12 for orientation-related metrics. Diameter, pore opening, coverage, and porosity all had small η^2^ values (≤0.031). These overlapping global averages indicate that the four programmed trajectories did not produce statistically distinguishable whole-ROI morphological descriptors under the investigated conditions. Although the XYZ motion programs define distinct needle paths and spatial sequences of jet presentation, the correspondence between these programmed paths and the resulting macroscopic fiber-deposition patterns was not directly quantified. Therefore, the present results support reproducible control of needle-motion geometry but do not establish quantitative control of the resulting spatial fiber distribution. The replicated SEM descriptors are summarized in Table 2. Fiber-diameter distributions, coverage and porosity, and FFT orientation are compared in Figure 7, Figure 8 and Figure 9, respectively. The PCA results are presented in Figure 10, and the inter-trajectory statistical comparisons are summarized in Table 3.

Exploratory PCA showed the same overlap in the global descriptor space. PC1 and PC2 explained 50.4% and 22.6% of the standardized variance, respectively. Mean silhouette was −0.187 and the ratio of mean between-group to within-group distance was 0.885. Because the PCA inputs were whole-ROI scalar descriptors, this overlap indicates that the trajectories were not cleanly separable using those global averages; it does not test whether the programmed stage imposed different macroscopic toolpaths or local spatial deposition histories. These multivariate results are therefore interpreted descriptively and not as a classifier-validation study.

### 3.7. Replicated Mechanical Comparison

**Figure 11:**
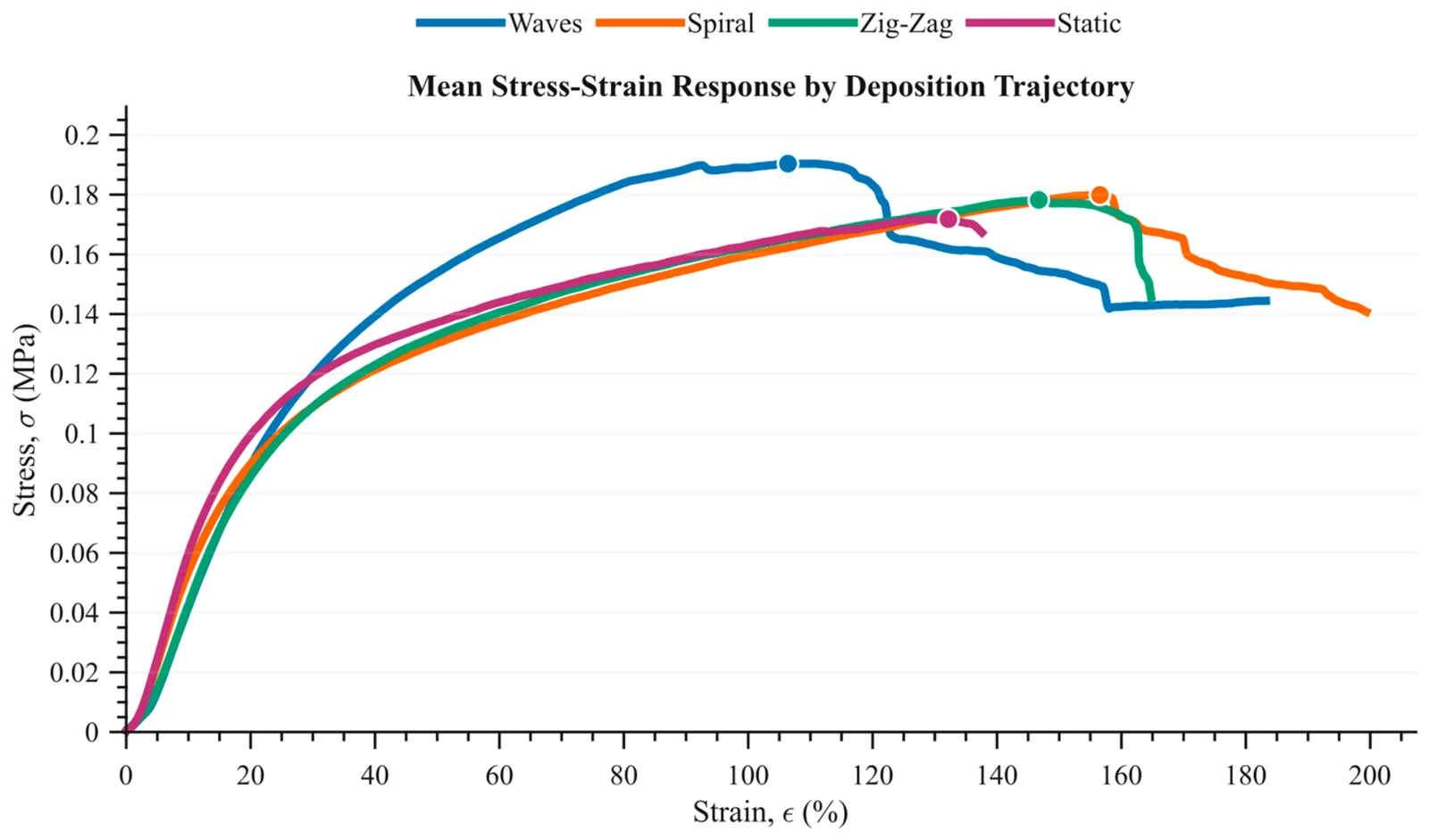
Comparison of equal-weight mean stress–strain curves for the four trajectories. Individual replicate curves are shown in Figure 3, Figure 4, Figure 5 and Figure 6.

Maximum tensile stress did not differ significantly among trajectories (one-way ANOVA F = 0.191, *p* = 0.900; Kruskal–Wallis H = 0.993, *p* = 0.803; η^2^ = 0.046). The apparently higher mean stress of Waves and Static was therefore not statistically supported, particularly because Static showed substantial between-specimen variability. These results are reported as geometry-normalized comparative responses, not as standardized material constants. The trajectory-level mean curves and replicated tensile descriptors are shown in Figure 11 and Table 4, respectively.

## 4. Discussion

### 4.1. Programmed Needle Trajectories and Their Relationship to Global Microstructural Descriptors

The principal outcome of the replicated experiment is a two-scale distinction between programmed deposition geometry and whole-ROI microstructural averages. Spiral, Waves, Zig-Zag, and Static imposed clearly different and reproducible macroscopic needle paths; however, the image-level scalar descriptors extracted from the resulting fibrous networks were not statistically separable under the tested processing window. This finding is more precise than the original n = 1 interpretation because it shows that geometric control of the commanded deposition path can coexist with overlapping mean fiber diameter, pore, coverage, porosity, and orientation metrics.

Statistical overlap of global descriptors should not be interpreted as absence of geometric influence. Programmed X–Y motion deterministically defines the needle position and the temporal sequence of jet presentation over the collector. However, electrohydrodynamic instabilities prevent a direct correspondence between the programmed needle position and the final location of deposited fibers. At the same time, bending instability and whipping introduce stochasticity between the commanded needle position and the final landing position of individual fibers. Consequently, trajectory effects are expected to be expressed most directly in the spatial organization and deposition history of the mat, whereas whole-ROI averages can integrate away part of that information. Future validation should therefore pair replicated global descriptors with direct trajectory–fiber orientation correlation, spatial autocorrelation, local coverage heterogeneity, and distance-to-commanded-path analyses.

The absence of statistically significant differences should not be interpreted as evidence of morphological equivalence among trajectories. With only four independent depositions per group, the analyses may have limited statistical power to detect small or moderate effects. Therefore, the findings indicate that trajectory-dependent differences were not statistically detected under the investigated conditions, rather than demonstrating equivalent microstructural outcomes.

### 4.2. Fiber Diameter Versus Deposition Redistribution

The absence of a significant difference in mean fiber diameter is mechanistically expected and is consistent with the proposed separation between fiber formation and deposition redistribution. Fiber diameter is established primarily during jet stretching, solvent evaporation, and electrohydrodynamic thinning before deposition, whereas X–Y needle motion primarily changes where already formed fibers are delivered, revisited, and overlapped on the collector. Thus, the programmed geometry can influence the observed deposition pattern without necessarily changing the average diameter of individual fibers. In the present data, mean diameter varied only from 1.719 to 1.791 µm across trajectory means and the ANOVA effect size was η^2^ = 0.012.

### 4.3. Pore Metrics and the Limitations of 2D SEM

Coverage, porosity, and pore opening were derived from two-dimensional SEM projections. They are therefore surface-projection descriptors and do not quantify through-thickness pore connectivity, tortuosity, or volumetric porosity. The common ROI and physical calibration improve comparability among images, but the measurements should not be interpreted as 3D pore-throat dimensions. For scaffold applications, a mean 2D pore-opening value near 2 µm cannot by itself demonstrate accessibility to mammalian cells. Future work should combine SEM with through-thickness or volumetric methods such as micro-CT, confocal reconstruction, or complementary porosimetry where appropriate [10,19].

### 4.4. Mechanical Response and Test-Method Limitations

The replicated tensile tests also showed no statistically significant trajectory effect. The most important observation was the magnitude of within-group dispersion, especially in Static. Several methodological factors can contribute to this variability in porous fibrous mats: local thickness differences, nonuniform areal mass, fiber rearrangement, grip compliance, and the use of a load cell with substantially greater capacity than the measured forces. Because GSM was not measured, equal basis weight cannot be demonstrated, and differences in deposited mass may contribute to the stress–strain response.

The standardization issue is also important. ASTM D638 covers plastics in standard dumbbell specimens, while ASTM D882 is preferred by ASTM for thin plastic sheeting below 1 mm [28,29]. The 0.3 mm fibrous strips used here do not constitute a formal D638 specimen, and porous nonwoven behavior differs from homogeneous film behavior. Accordingly, the present study does not claim standard-compliant tensile strength or elastic modulus. The reported stress values were calculated using the nominal specimen cross-sectional area rather than individually measured effective load-bearing areas. Local variations in thickness, porosity, and deposited mass may therefore affect the calculated values. Furthermore, the measurement uncertainty associated with the 5 kN load cell in the low-force range was not independently established. The mechanical results should consequently be interpreted as apparent, protocol-dependent tensile responses rather than intrinsic material properties. Future studies should incorporate a lower-capacity load cell, direct strain measurement, specimen-specific thickness and basis-weight characterization, and a validated testing protocol appropriate for thin fibrous mats.

### 4.5. Hybrid 3D-Printing Motion Versus Conventional Electrospinning

The 3D-printing contribution in this system is programmable motion rather than fused-filament fabrication of the PCL fibers. Compared with a static electrospinning setup, the XYZ stage provides repeatable macro-scale positioning, path geometry, speed control, revisit frequency, and spatial deposition history. Compared with material-extrusion 3D printing, electrospinning provides much finer fibrous topography and higher surface area but does not offer deterministic placement of each fiber. Hybrid approaches therefore trade exact filament placement for multiscale architecture [7,8,9]. The replicated results demonstrate that the platform reliably imposes distinct macroscopic deposition paths, while also showing that programmed motion alone does not guarantee statistically distinct whole-ROI microstructural averages. The principal contribution of the platform is the integration of electrospinning with programmable XYZ needle motion, rather than demonstrated control of the resulting fiber-deposition pattern or individual fiber dimensions. A comparison with representative literature is presented in Table 5.

### 4.6. Unmeasured Material Parameters and Future Work

Three parameters requested during peer review—solution viscosity/rheology, polymer PDI, and basis weight (GSM)—were not measured during the completed experimental campaign. The revised manuscript therefore does not invent or back-calculate these values. Their absence limits complete reproduction and the ability to distinguish trajectory effects from solution-state or deposited-mass variability. If residual mats are available, basis weight can still be measured directly by weighing coupons of known area. PDI should be obtained from the supplier lot certificate or measured by SEC/GPC, and rheology should be measured on a freshly prepared formulation using the same composition and conditioning protocol; any such later measurement should be identified as supplementary formulation characterization rather than as a retrospective measurement of the original batch.

Future experiments should increase deposition-level sample size, measure solution viscosity and conductivity, document polymer molecular-weight distribution, control or measure basis weight and thickness at multiple locations, use a lower-capacity load cell, adopt a thin-sheet/nonwoven-appropriate tensile protocol, and include volumetric pore characterization. A factorial design varying trajectory geometry together with translation speed or electric-field configuration may be more informative than changing trajectory shape alone.

## 5. Conclusions

The main contribution of this study is the development and experimental evaluation of a custom-designed electrospinning platform incorporating the programmable XYZ motion system of a 3D printer. Unlike hybrid manufacturing approaches that sequentially combine 3D-printed structures and electrospun fibrous layers, the proposed system enables continuous fiber formation and deposition while the electrospinning needle follows predefined trajectories. By integrating programmable motion directly into the electrospinning process, without material extrusion, this platform provides an alternative technological approach for investigating the spatial organization of fibrous scaffolds.

The experimental evaluation of four programmed trajectories (Spiral, Static, Waves, and Zig-Zag), using four independent depositions per condition, revealed no statistically significant differences in the principal global morphological descriptors or apparent maximum tensile stress. Multivariate analysis also showed substantial overlap among experimental groups. These findings indicate that distinct programmed needle trajectories do not necessarily produce distinguishable global morphological or mechanical responses under the investigated processing conditions.

Although the platform enables reproducible programming of needle motion, the relationship between the programmed trajectories and the resulting spatial distribution of deposited fibers remains to be quantitatively established. The interpretation of the findings is also constrained by the limited number of independent depositions, the absence of independent quantitative validation of the automated SEM analysis, and uncertainties associated with the tensile-testing protocol. Consequently, the lack of statistically significant differences should not be interpreted as evidence of equivalence among trajectories.

Overall, this study demonstrates the feasibility of integrating electrospinning with programmable XYZ motion within a single fabrication platform and establishes a basis for further investigation of trajectory-dependent fibrous architectures. Future research should focus on quantitative spatial deposition mapping, validated image-analysis procedures, improved mechanical characterization, and optimization of processing parameters to determine how programmed motion can be translated into controlled scaffold microarchitecture.

## Figures and Tables

**Table 1:** Geometric definition of the programmed X–Y trajectories reconstructed from the trajectory files.

Trajectory	Path Definition	Amplitude/Radius	Pitch/Period	Additional Geometry
Spiral	Translating elliptical-loop sweep	5 mm X semi-axis; 6 mm Y semi-axis	6 loops per row; horizontal pitch ≈ 11.67 mm/rev	Initial = final semi-axes (5, 6 mm); five rows, 12 mm row pitch
Static	Stationary point	X = 50 mm; Y = 40 mm	Not applicable	No X–Y motion
Waves	Sinusoidal sweep	Amplitude = 5.5 mm	Spatial period = 20 mm	Five rows; 12 mm row pitch; approximate temporal period 12.3 s at 2.5 mm/s path speed
Zig-Zag	Triangular-wave sweep	Amplitude = 5.5 mm	Spatial period = 20 mm	Segment Δx = 10 mm, Δy = 11 mm; length = 14.87 mm; heading ±47.7°; direction change ≈95.5°

**Table 2:** Replicated SEM descriptors reported as mean ± SD across four independent depositions per trajectory (*n* = 4).

Trajectory	Diameter (µm)	Pore Opening (µm)	Coverage	Porosity	FFT Alignment	Angular Spread (°)
Spiral	1.736 ± 0.235	1.984 ± 0.073	0.560 ± 0.015	0.440 ± 0.015	0.138 ± 0.090	47.98 ± 3.68
Static	1.791 ± 0.335	2.028 ± 0.127	0.546 ± 0.071	0.454 ± 0.071	0.105 ± 0.025	48.64 ± 1.11
Waves	1.752 ± 0.252	1.994 ± 0.061	0.553 ± 0.020	0.447 ± 0.020	0.114 ± 0.037	48.44 ± 1.71
Zig-Zag	1.719 ± 0.274	2.018 ± 0.168	0.544 ± 0.031	0.456 ± 0.031	0.095 ± 0.040	48.74 ± 1.05

**Table 3:** Inter-trajectory statistical comparison of principal SEM descriptors (*n* = 4 independent depositions per trajectory).

Descriptor	ANOVA *p*	Kruskal–Wallis *p*	η^2^
Mean fiber diameter	0.985	0.866	0.012
Mean pore opening	0.941	0.897	0.031
Coverage	0.940	0.787	0.031
Porosity	0.940	0.787	0.031
FFT alignment index	0.726	0.792	0.100
FFT angular spread	0.960	0.931	0.024
Segment alignment index	0.673	0.608	0.116

**Table 4:** Replicated tensile-response descriptors (*n* = 4 specimens per trajectory).

Trajectory	Maximum Stress (MPa)	CV (%)	Peak of Mean Curve (MPa)	Strain at Mean-Curve Peak (%)
Spiral	0.185 ± 0.052	28.1	0.180	156.5
Static	0.214 ± 0.147	68.7	0.172	132.2
Waves	0.227 ± 0.079	34.5	0.190	106.4
Zig-Zag	0.189 ± 0.051	26.9	0.178	146.7

**Table 5:** Comparison with representative literature on collector patterning, alignment, and hybrid electrospinning/3D-printing strategies.

Study	Approach	Principal Observation	Relation to the Present Study
Clavijo-Grimaldo et al. [1]	Collector-dependent PCL electrospinning	Collector type altered membrane morphology and scaffold characteristics.	Supports collector/deposition history as a design variable.
Zhao et al. [3]	Structured non-conductive substrates	Topography enabled patterned nanofiber deposition.	Demonstrates substrate-guided spatial organization.
Palomares et al. [4]	Patterned electrospinning	Defined fibrous constructs affected cell adhesion/retention.	Shows functional consequences of imposed patterns.
García-Limón et al. [5]	XYZ electrospinning + induced fields	Trajectory orientation and jet collimation were enhanced by coordinated motion/field control.	Suggests motion may be more effective when coupled to field guidance.
Hosseinian et al. [6]	Origami-based far-field alignment	High fiber alignment obtained with a geometry-assisted strategy.	Provides an alignment benchmark stronger than that observed here.
Vyas et al. [9]	3D printing + electrospinning PCL	Dual-scale scaffolds promoted oriented fiber architecture and cell alignment.	Illustrates complementary macro/micro-scale hybridization.
Present study	Four programmed XYZ paths, *n* = 4	Distinct reproducible macroscopic deposition paths; whole-ROI SEM descriptors and maximum tensile stress remained statistically overlapping under the tested window.	Supports programmed trajectory geometry as a spatial deposition-control variable while distinguishing it from direct control of fiber formation or global scalar morphology.

**Figure A1:**
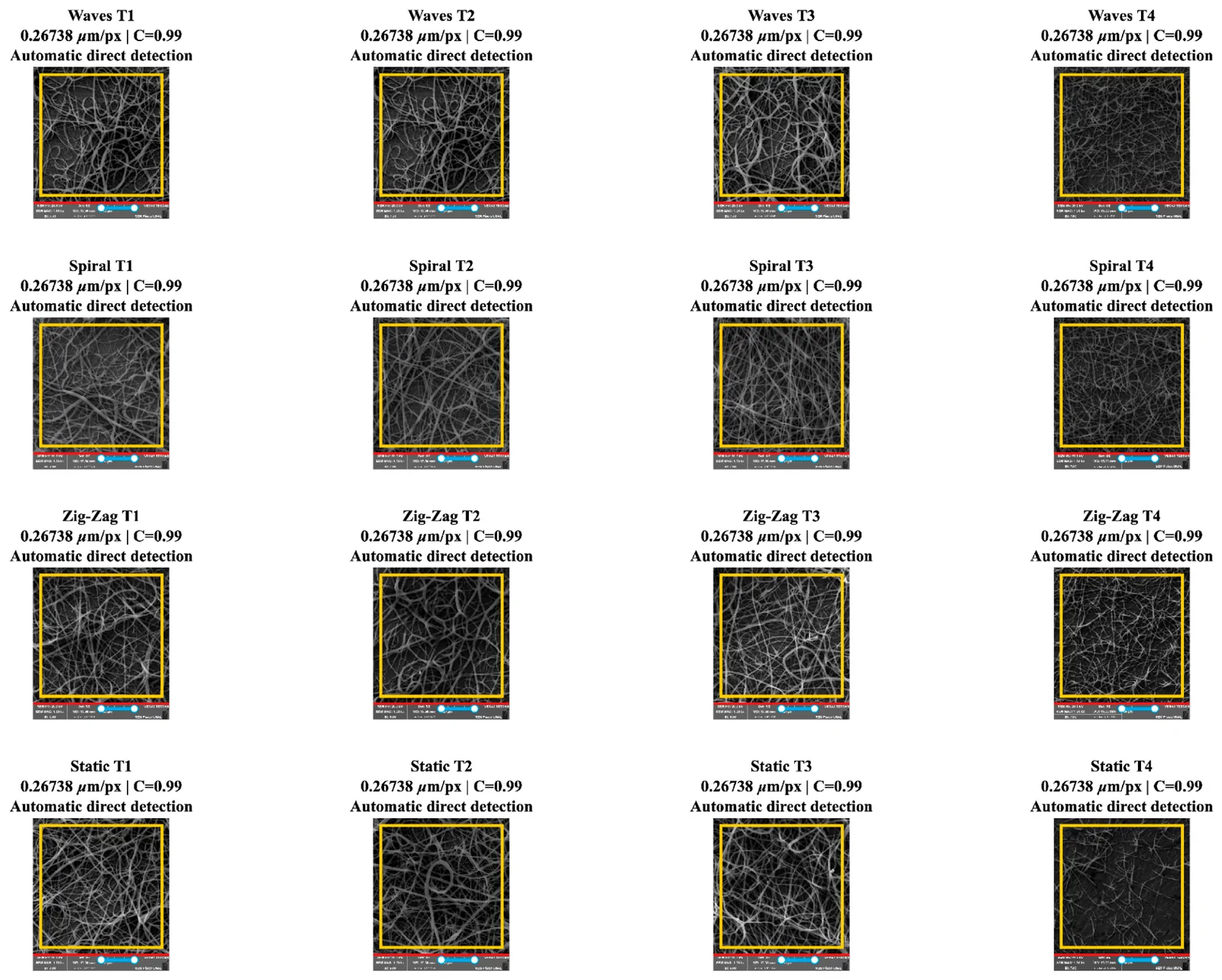
Automatic SEM scale-bar and common-ROI quality control for the 16 independent images. Red lines indicate the detected instrument-footer boundary, cyan marks the detected 50 µm scale bar, and yellow rectangles indicate the common physical analysis ROI.

**Figure A2:**
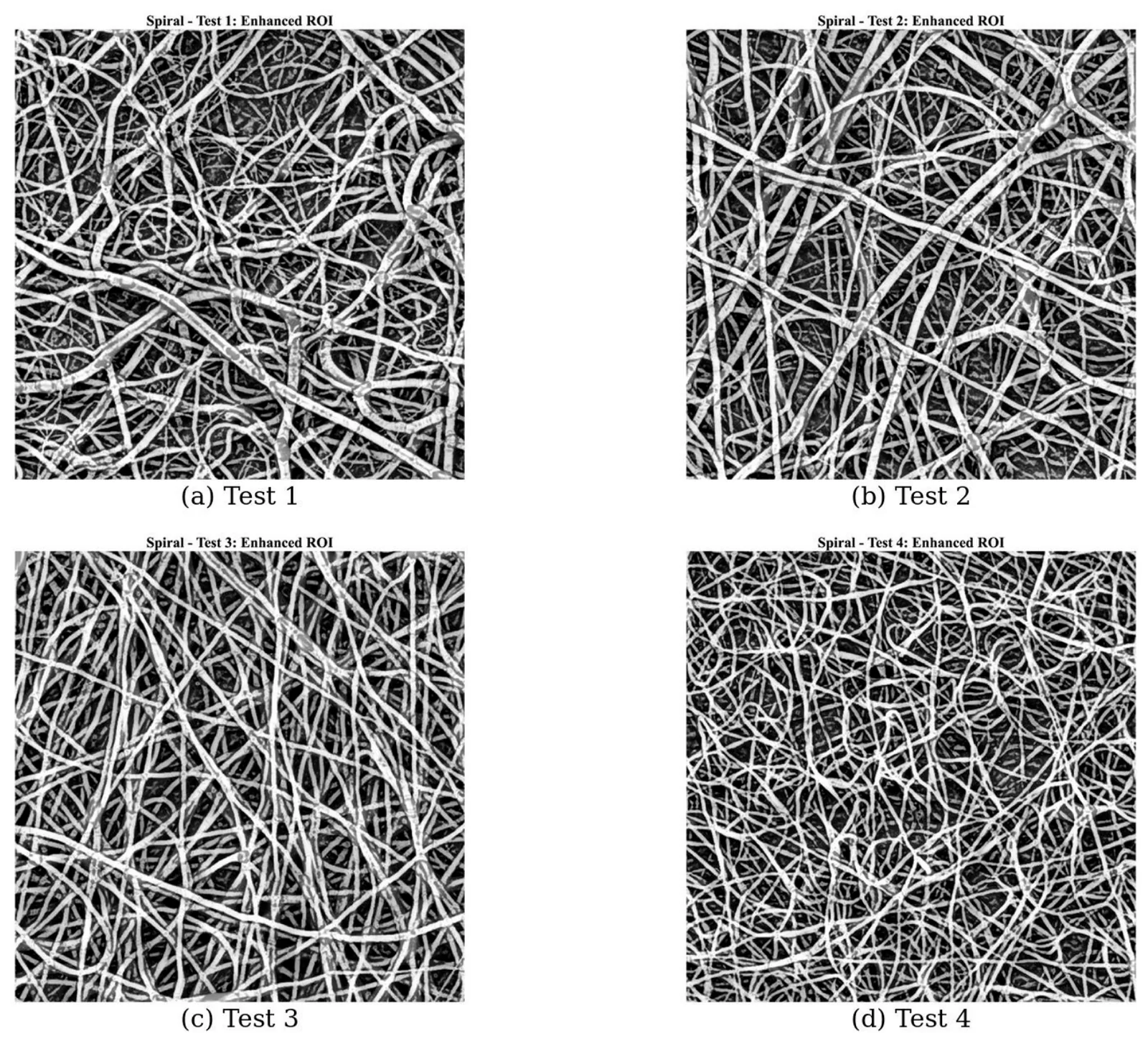
Spiral: enhanced ROI images for the four independent depositions (T1–T4).

**Figure A3:**
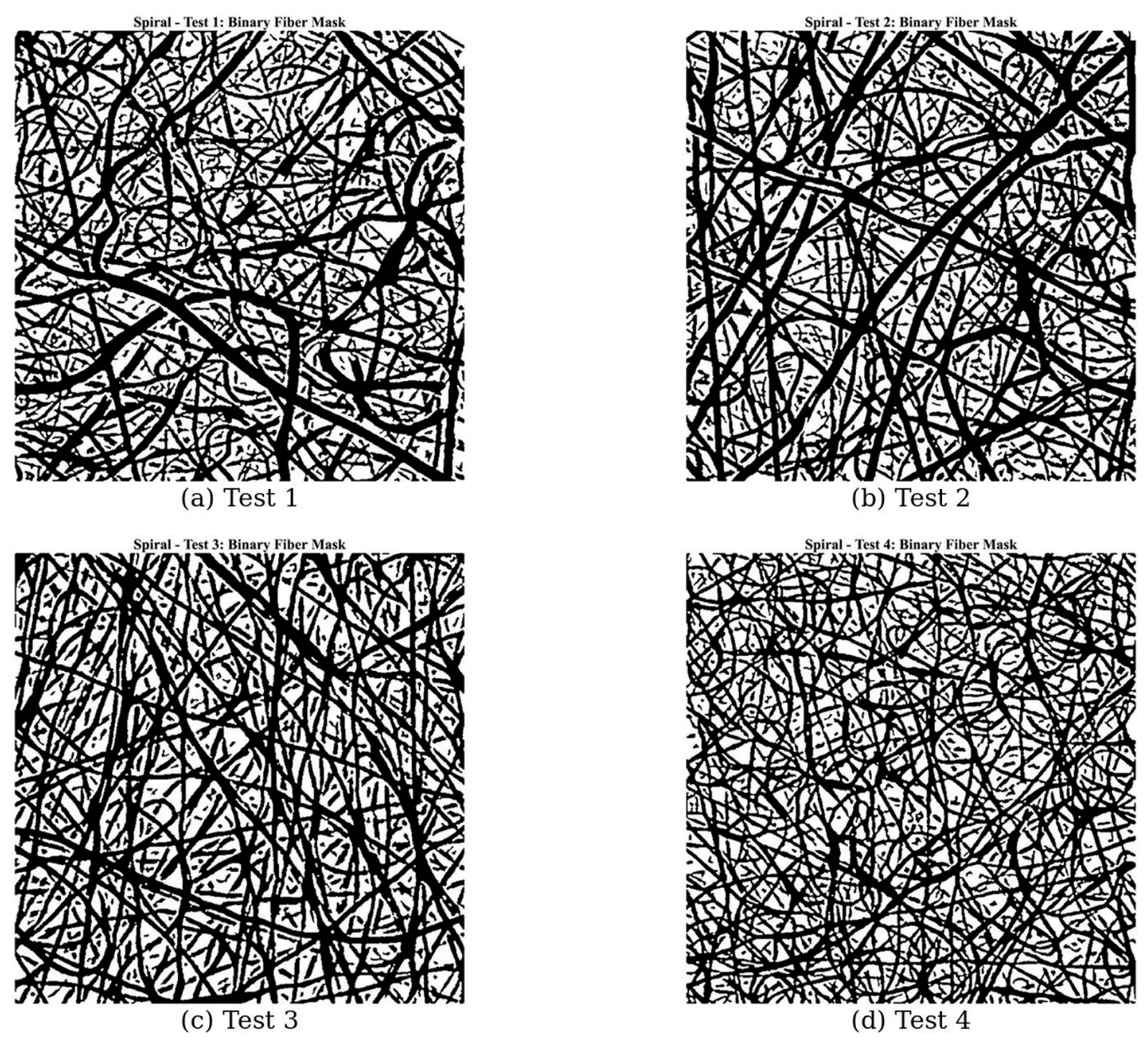
Spiral: binary fiber masks for the four independent depositions (T1–T4).

**Figure A4:**
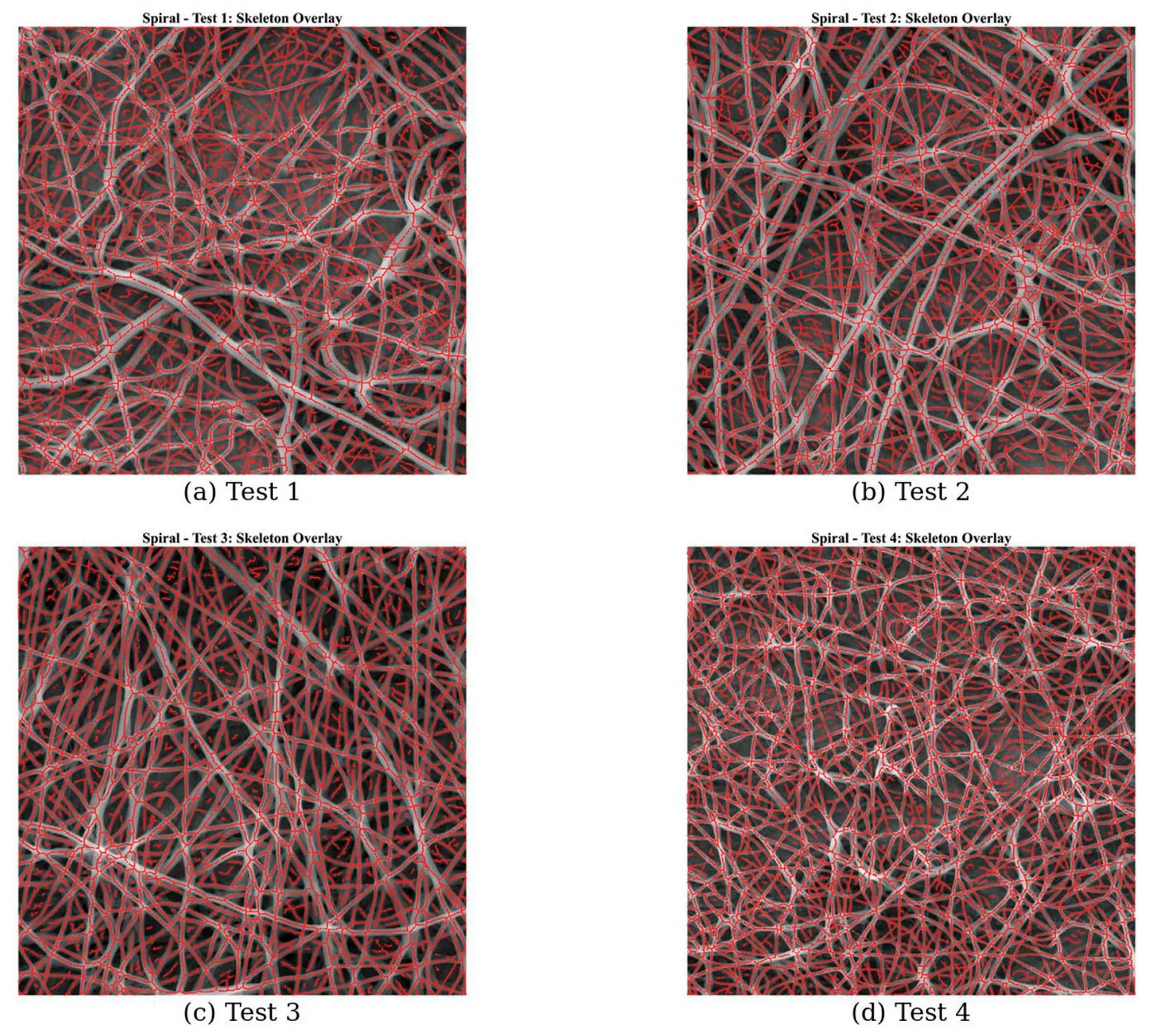
Spiral: skeleton overlays for the four independent depositions (T1–T4).

**Figure A5:**
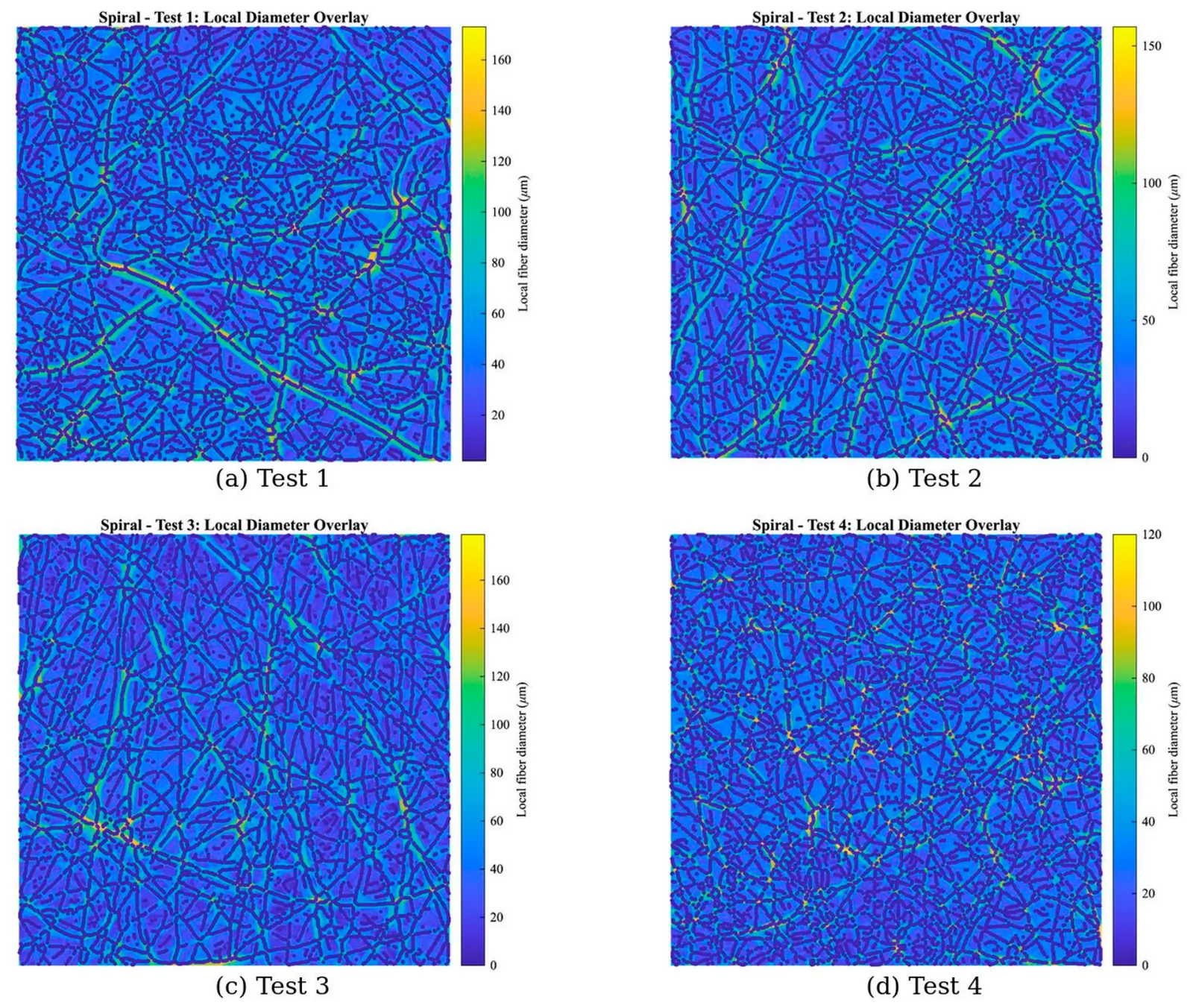
Spiral: local diameter overlays for the four independent depositions (T1–T4).

**Figure A6:**
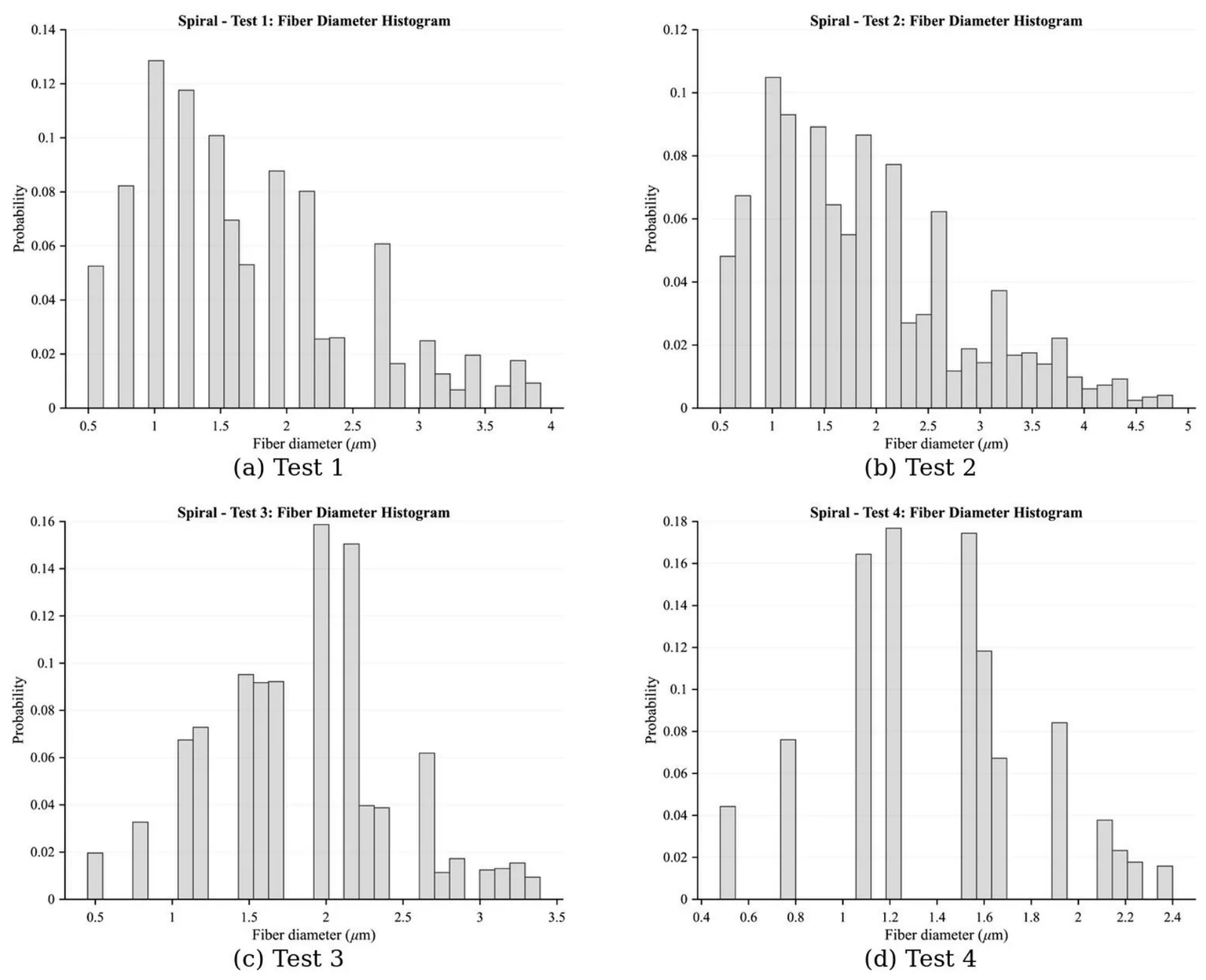
Spiral: fiber-diameter histograms for the four independent depositions (T1–T4).

**Figure A7:**
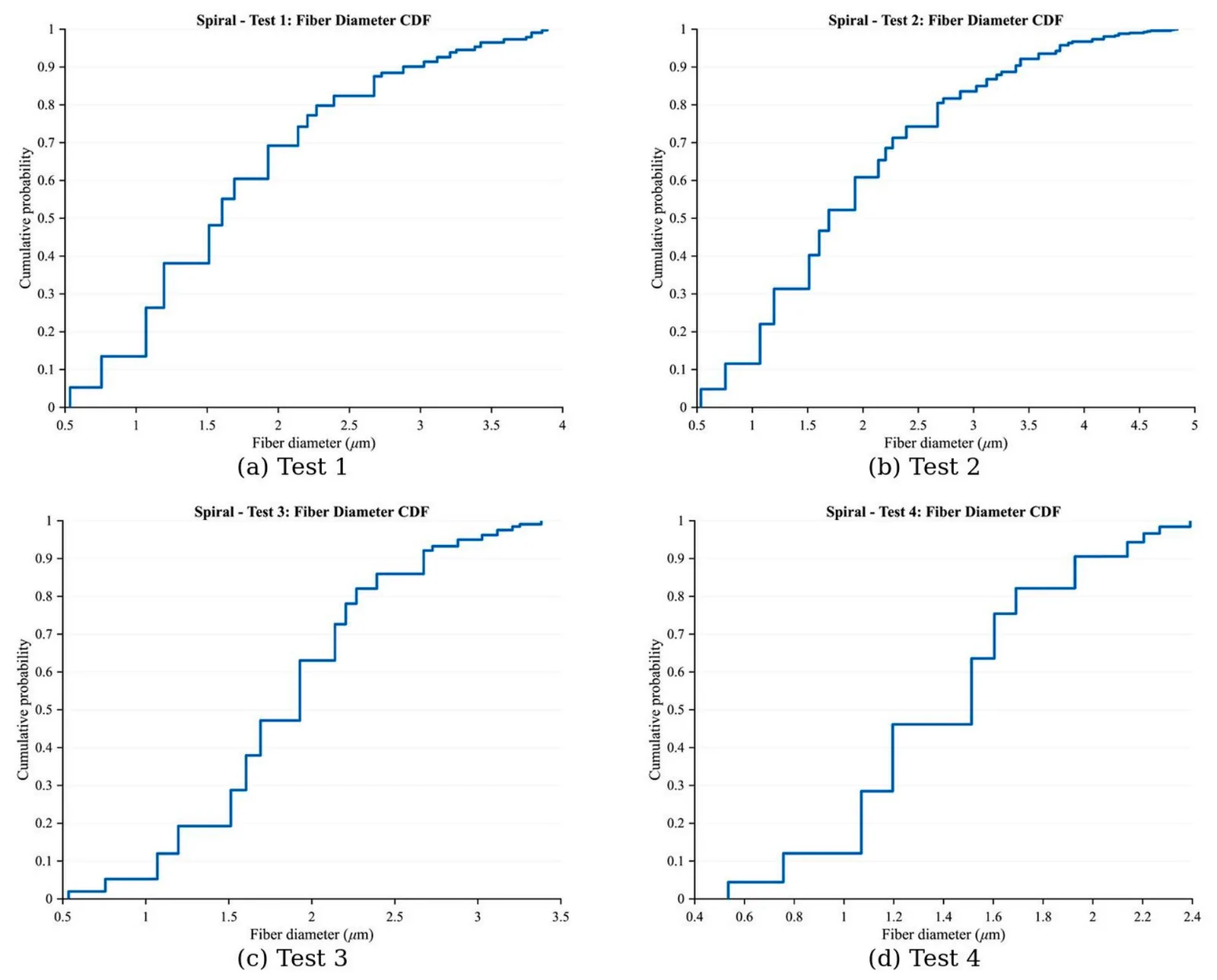
Spiral: fiber-diameter cumulative distributions for the four independent depositions (T1–T4).

**Figure A8:**
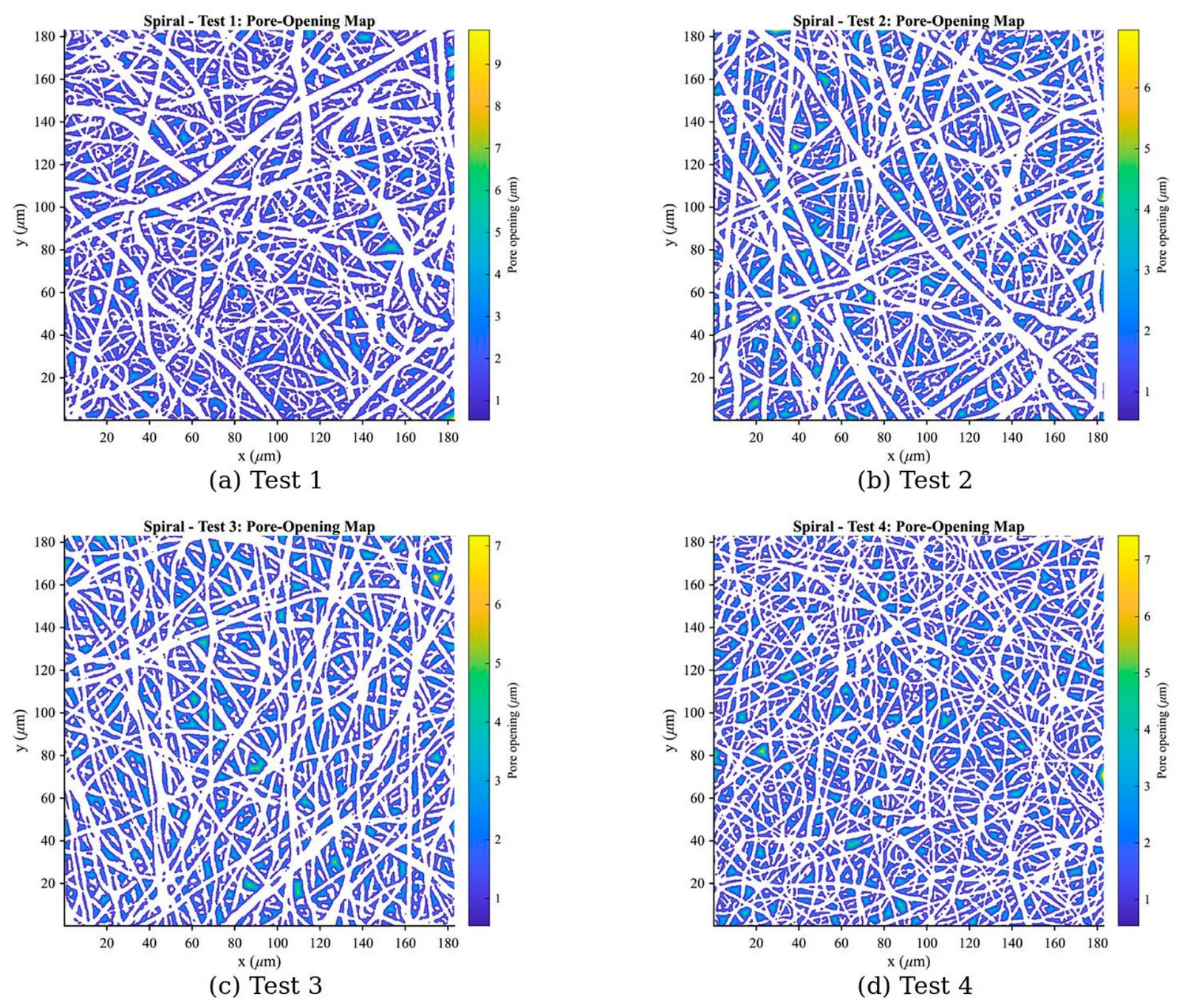
Spiral: local pore-opening maps for the four independent depositions (T1–T4).

**Figure A9:**
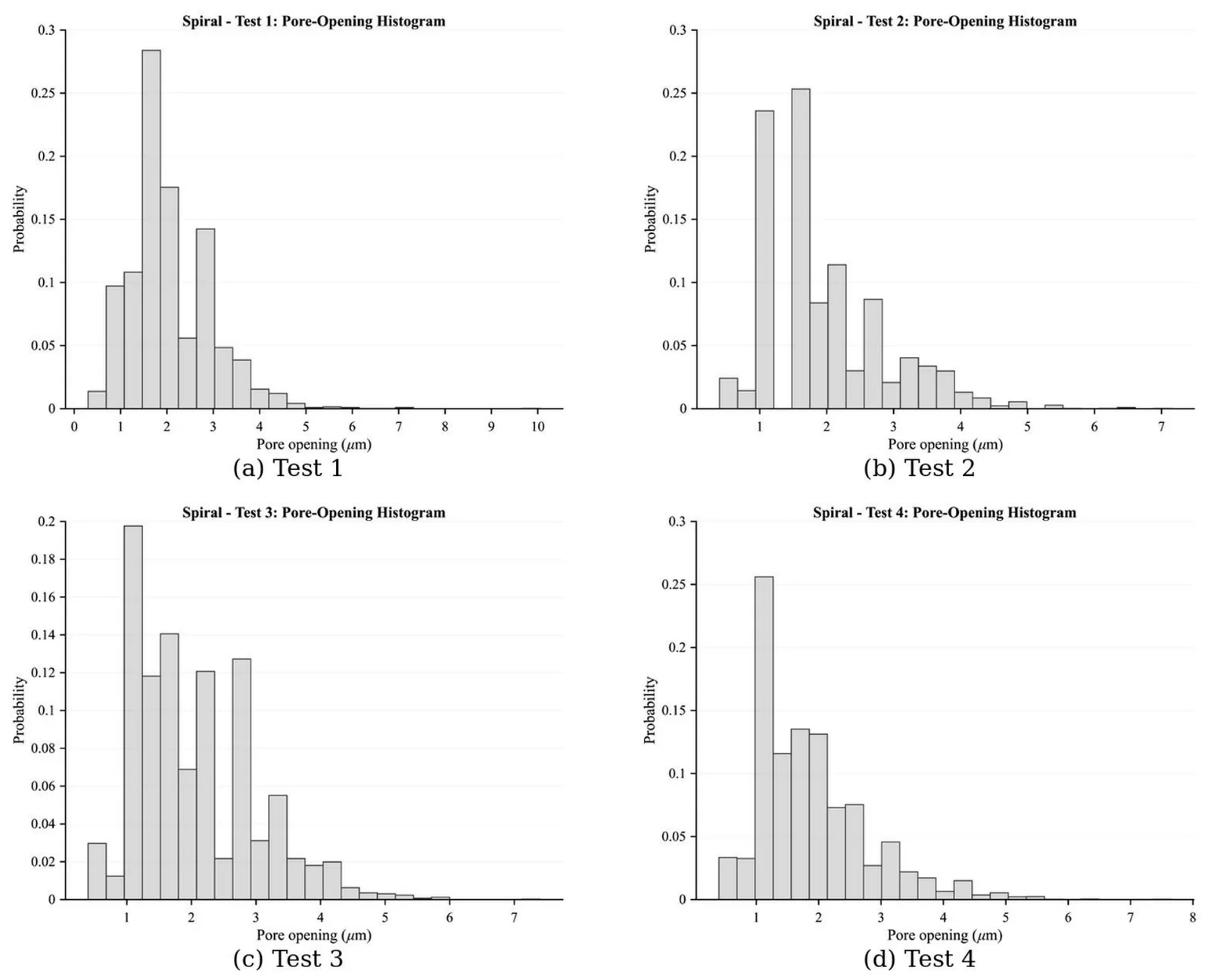
Spiral: pore-opening histograms for the four independent depositions (T1–T4).

**Figure A10:**
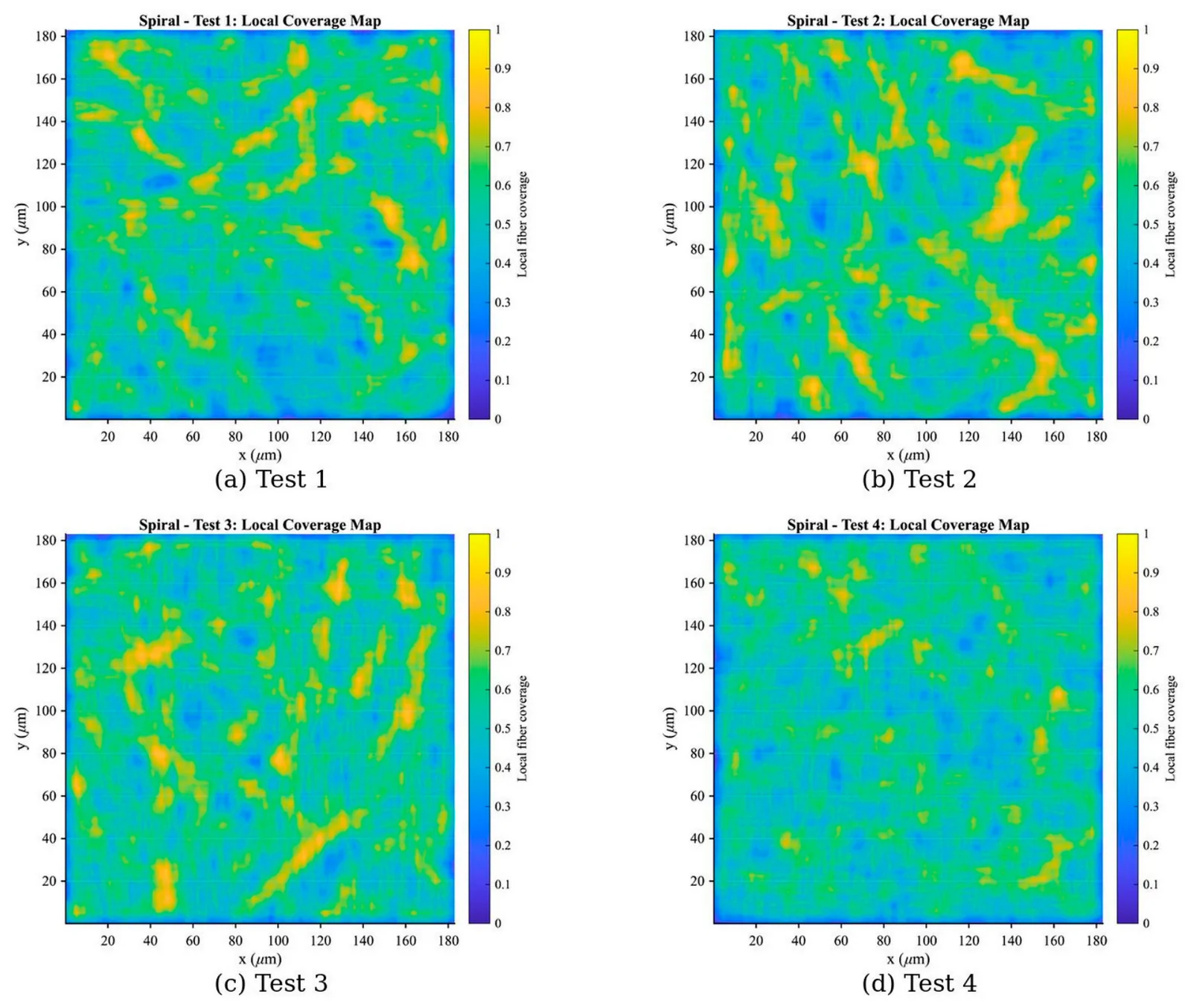
Spiral: local coverage maps for the four independent depositions (T1–T4).

**Figure A11:**
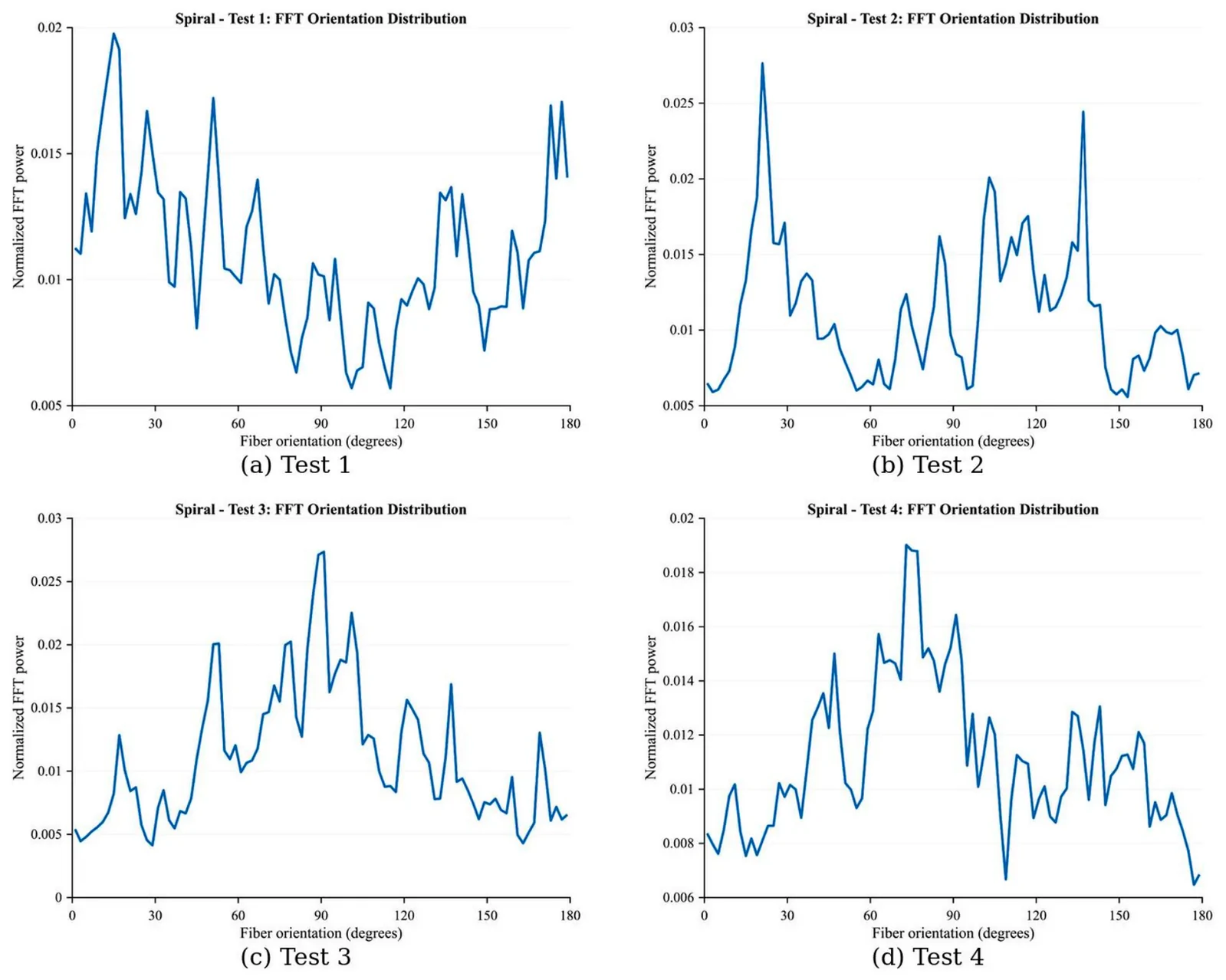
Spiral: FFT-based orientation distributions for the four independent depositions (T1–T4).

**Figure A12:**
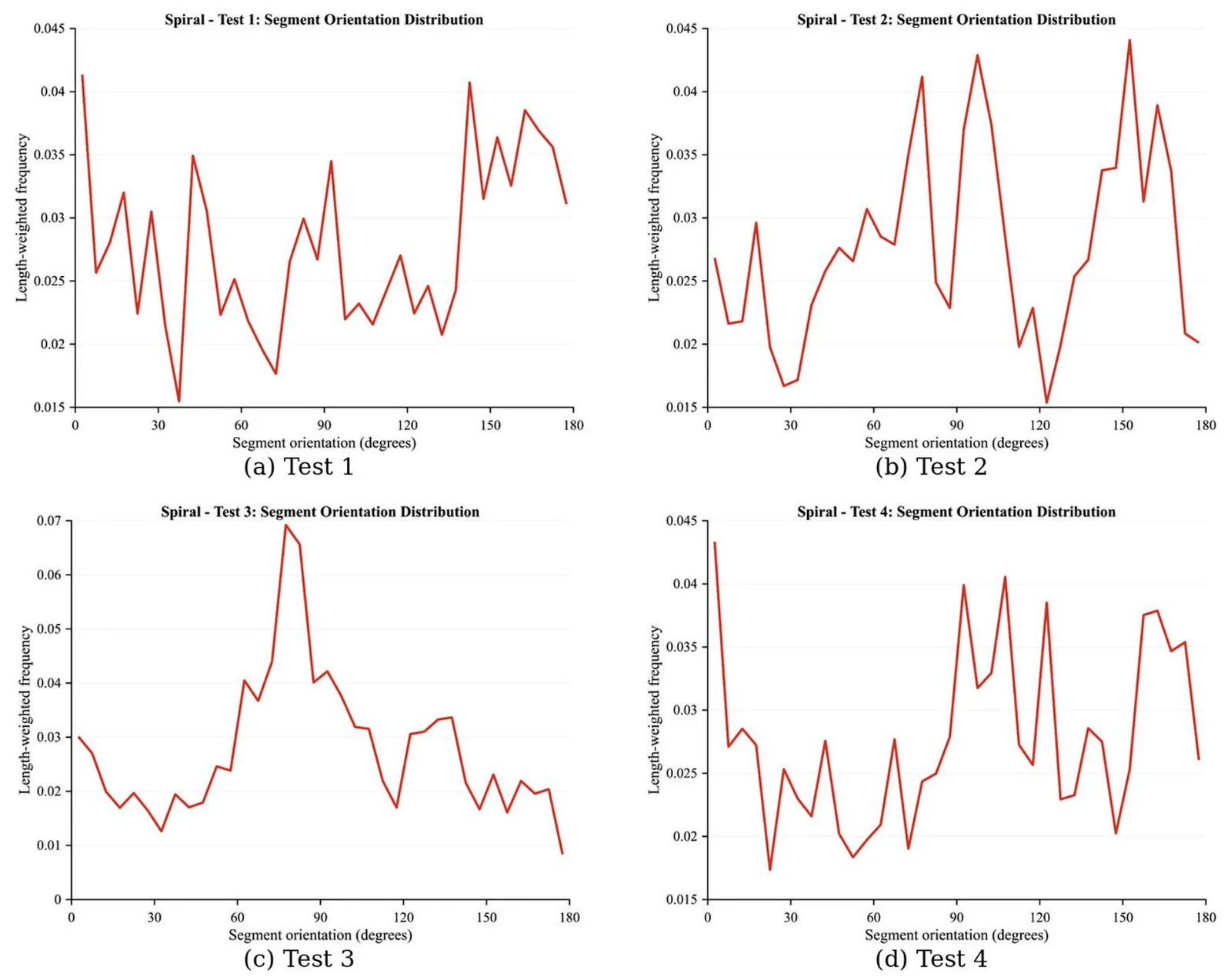
Spiral: skeleton-segment orientation distributions for the four independent depositions (T1–T4).

**Figure A13:**
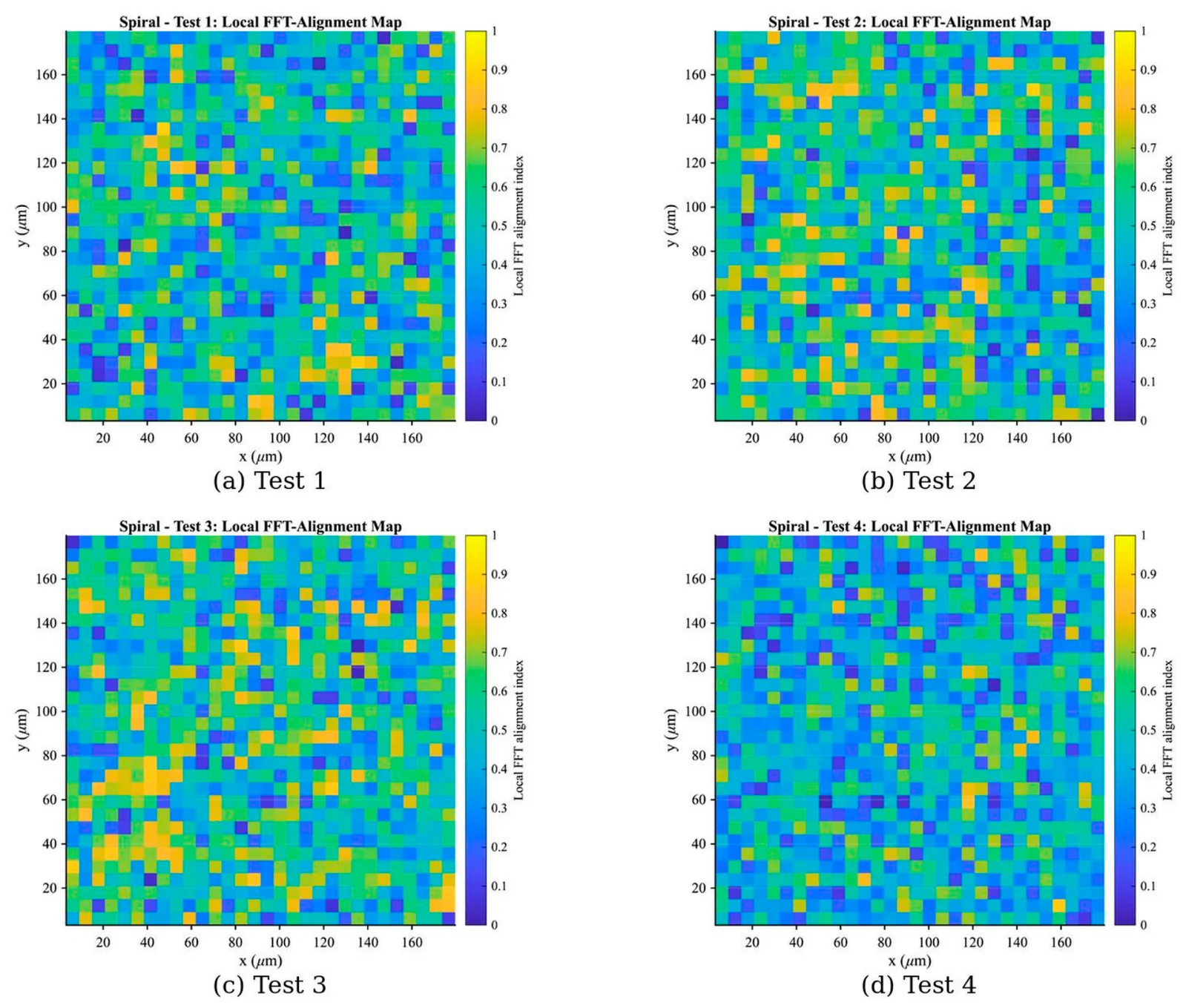
Spiral: local FFT-alignment maps for the four independent depositions (T1–T4).

**Figure A14:**
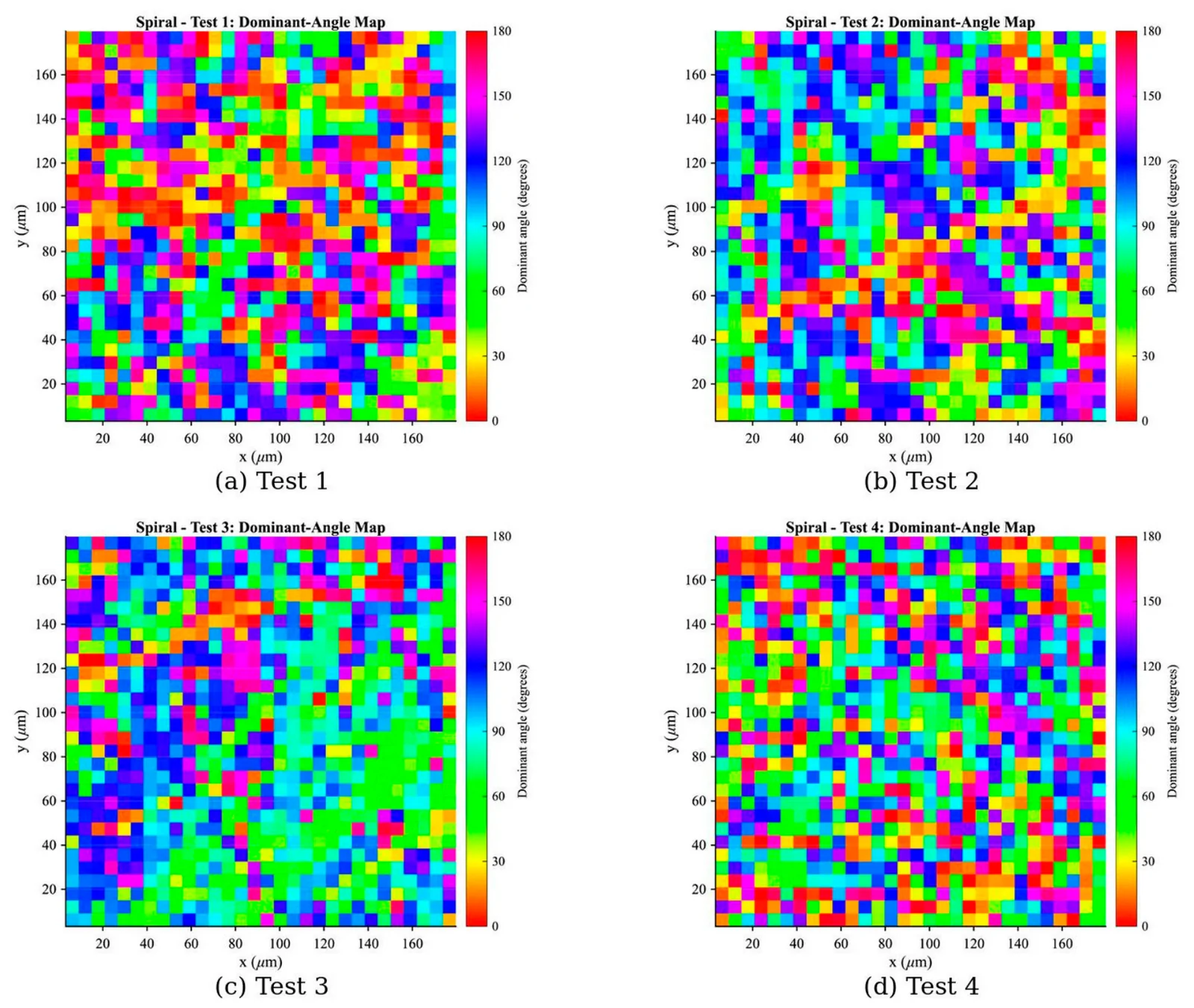
Spiral: dominant-angle maps for the four independent depositions (T1–T4).

**Figure A15:**
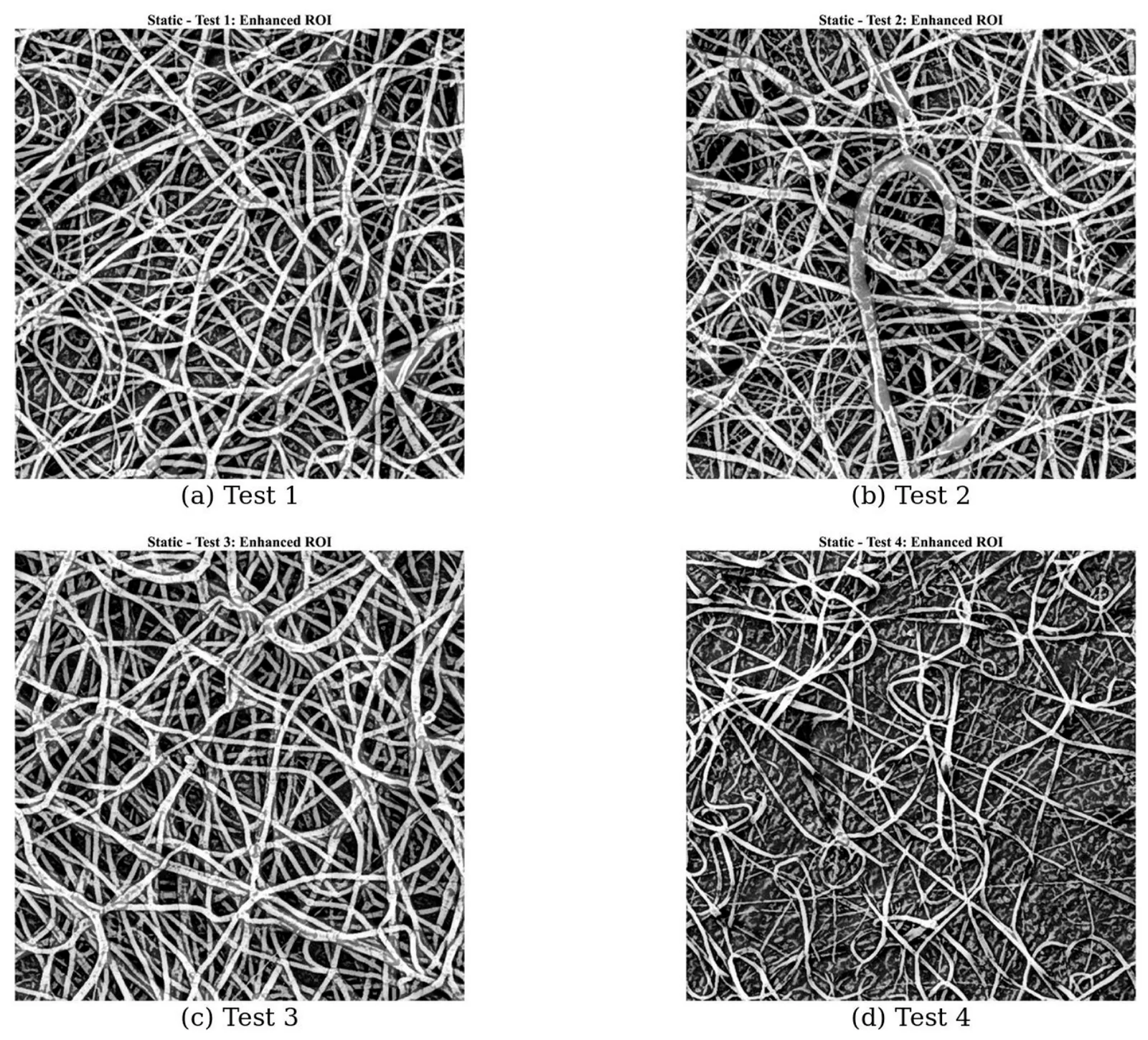
Static: enhanced ROI images for the four independent depositions (T1–T4).

**Figure A16:**
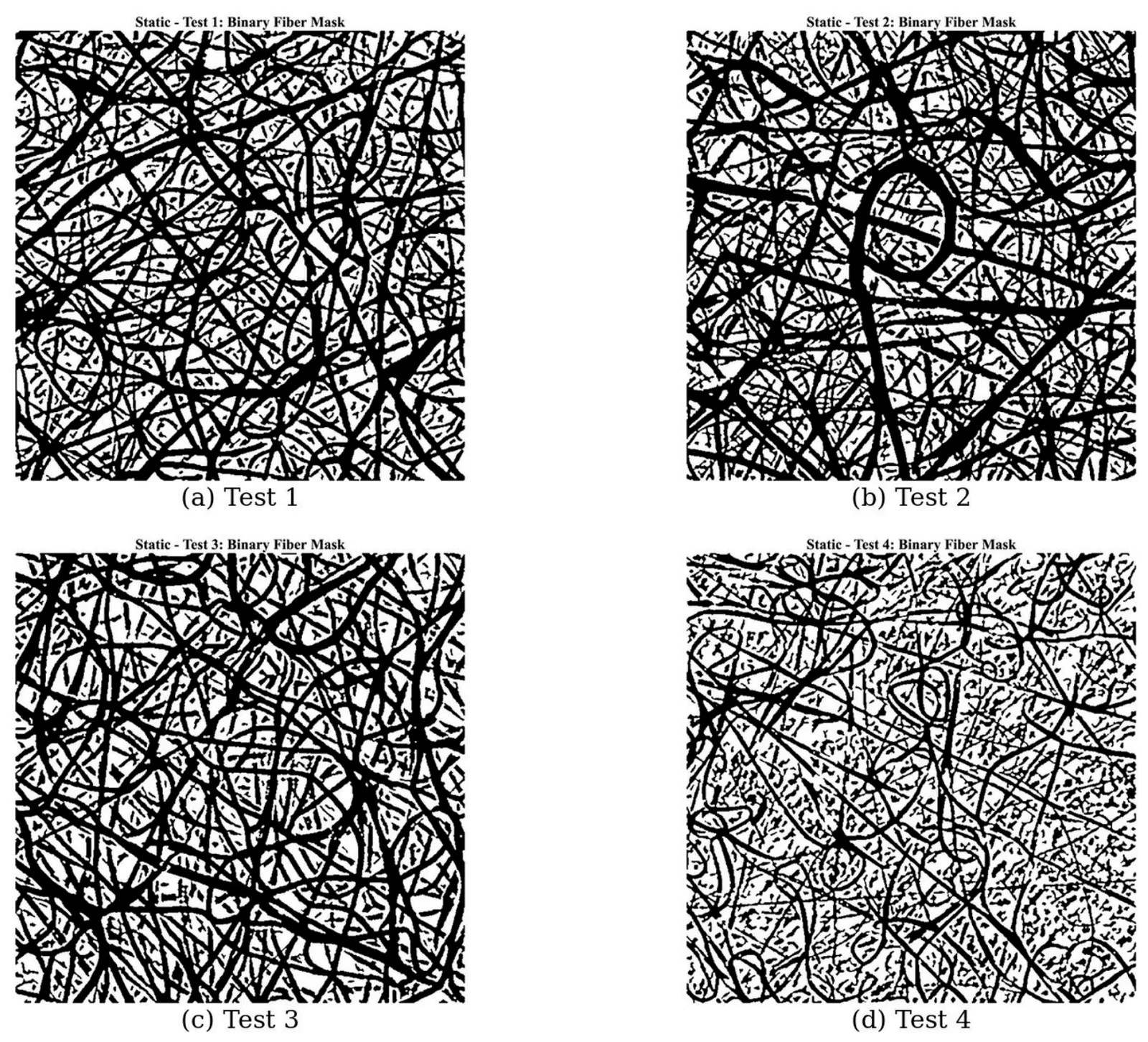
Static: binary fiber masks for the four independent depositions (T1–T4).

**Figure A17:**
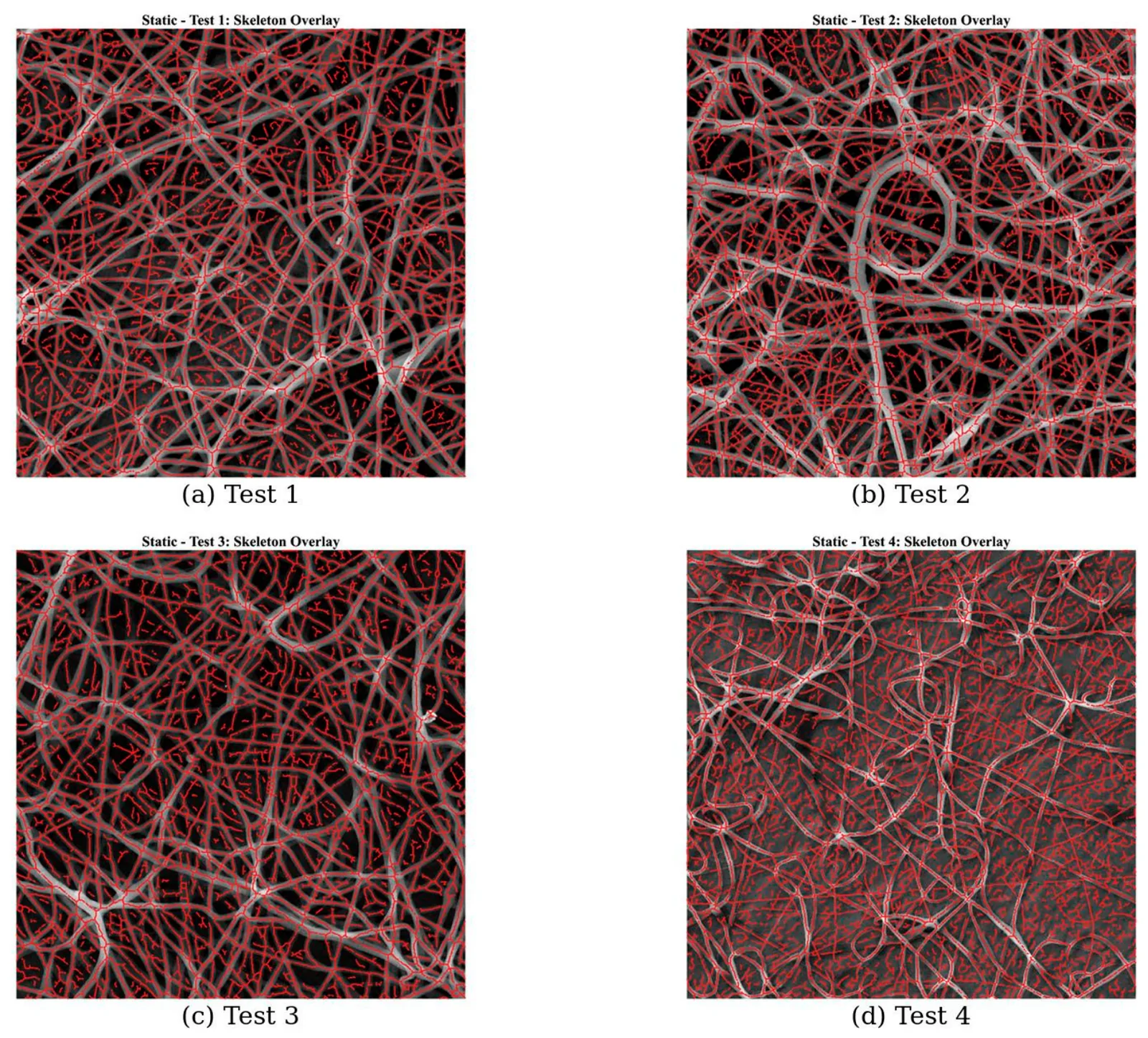
Static: skeleton overlays for the four independent depositions (T1–T4).

**Figure A18:**
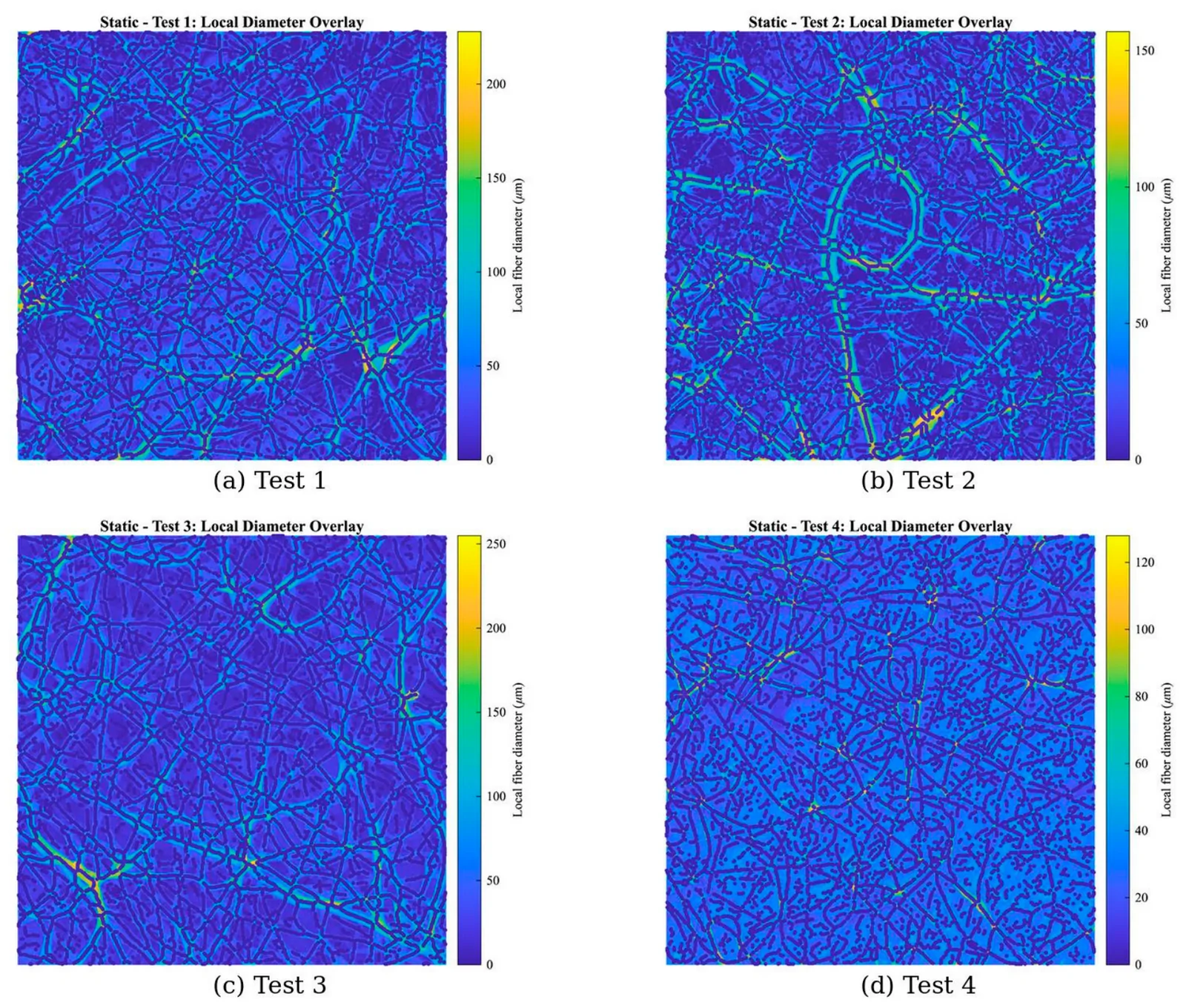
Static: local diameter overlays for the four independent depositions (T1–T4).

**Figure A19:**
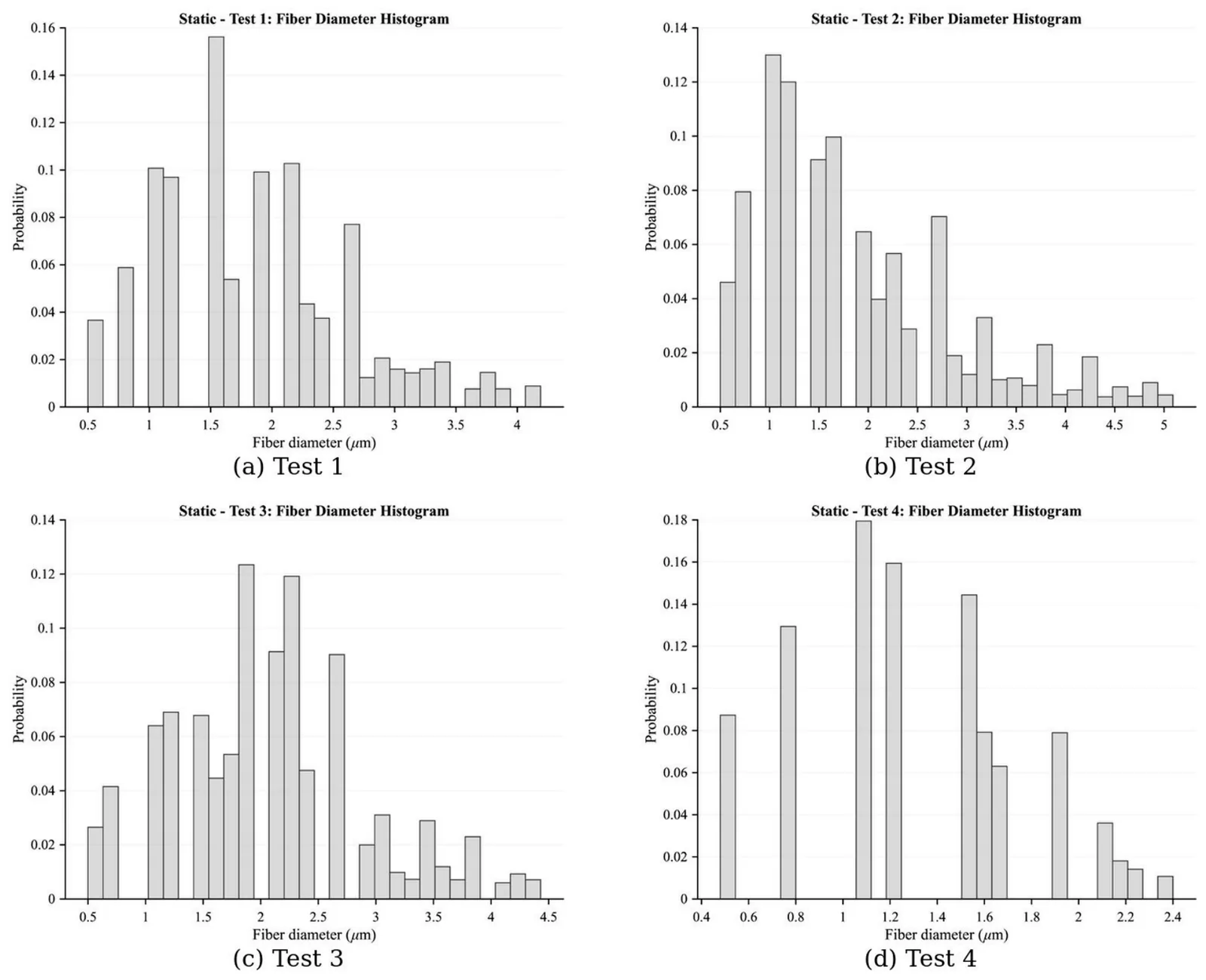
Static: fiber-diameter histograms for the four independent depositions (T1–T4).

**Figure A20:**
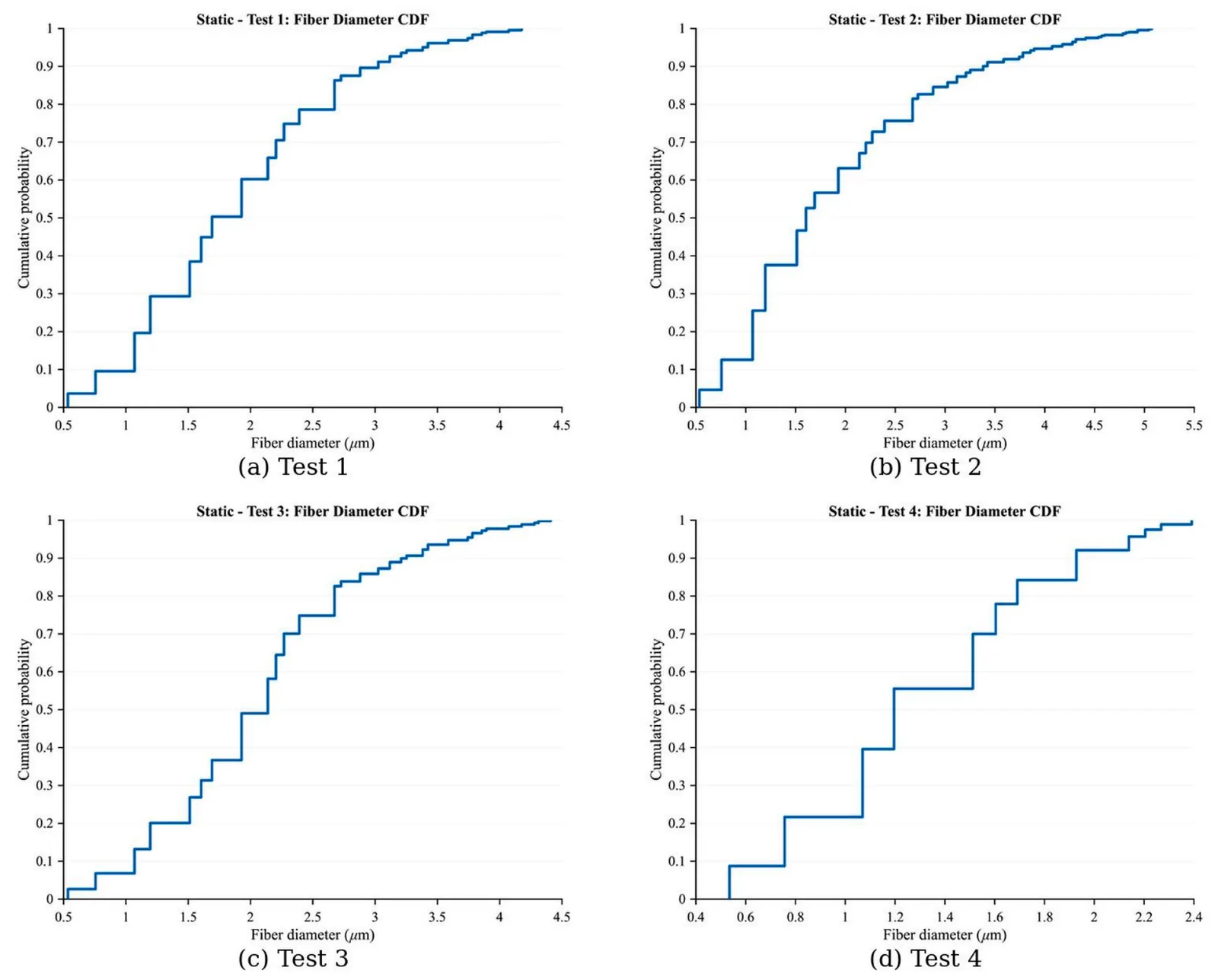
Static: fiber-diameter cumulative distributions for the four independent depositions (T1–T4).

**Figure A21:**
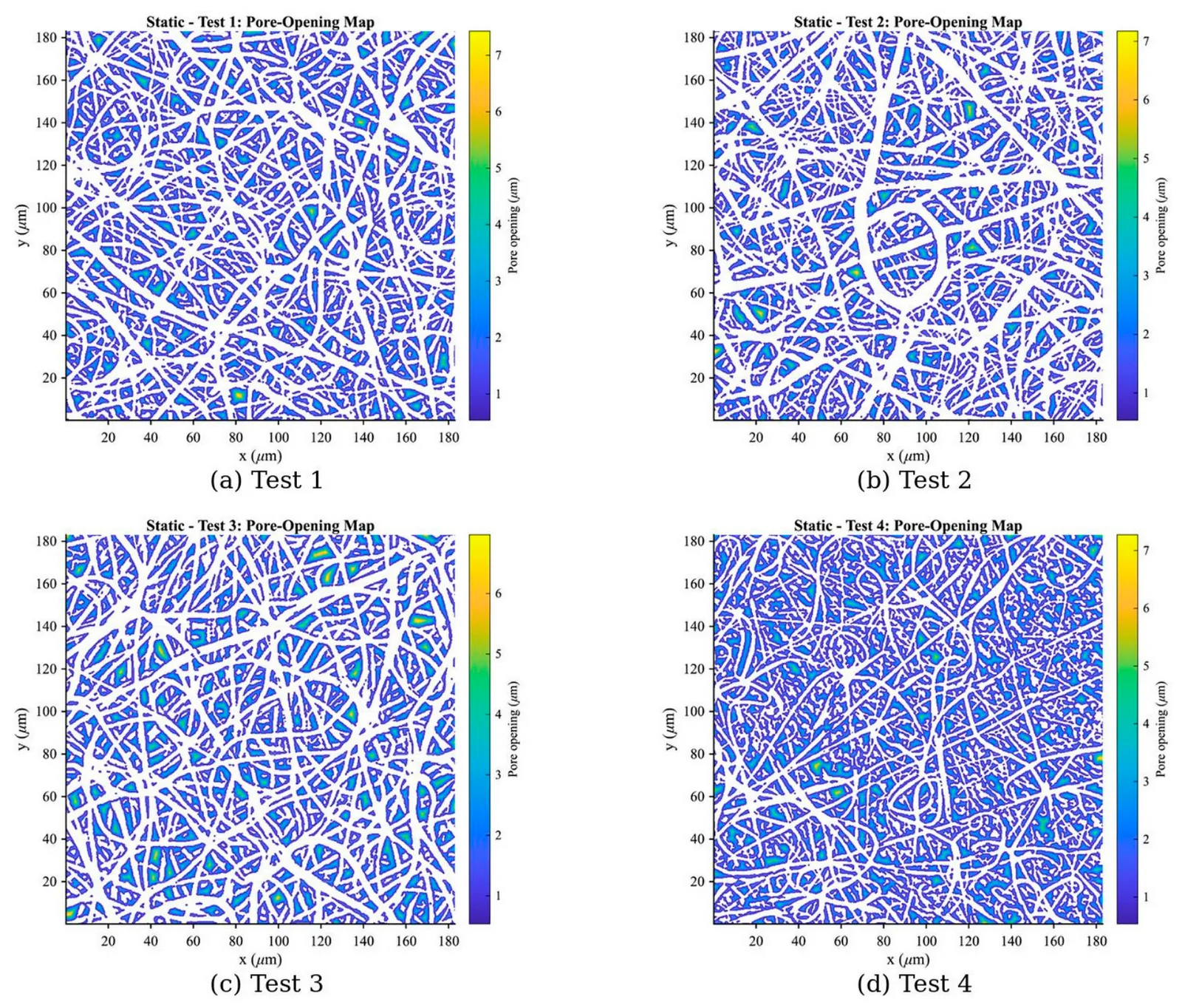
Static: local pore-opening maps for the four independent depositions (T1–T4).

**Figure A22:**
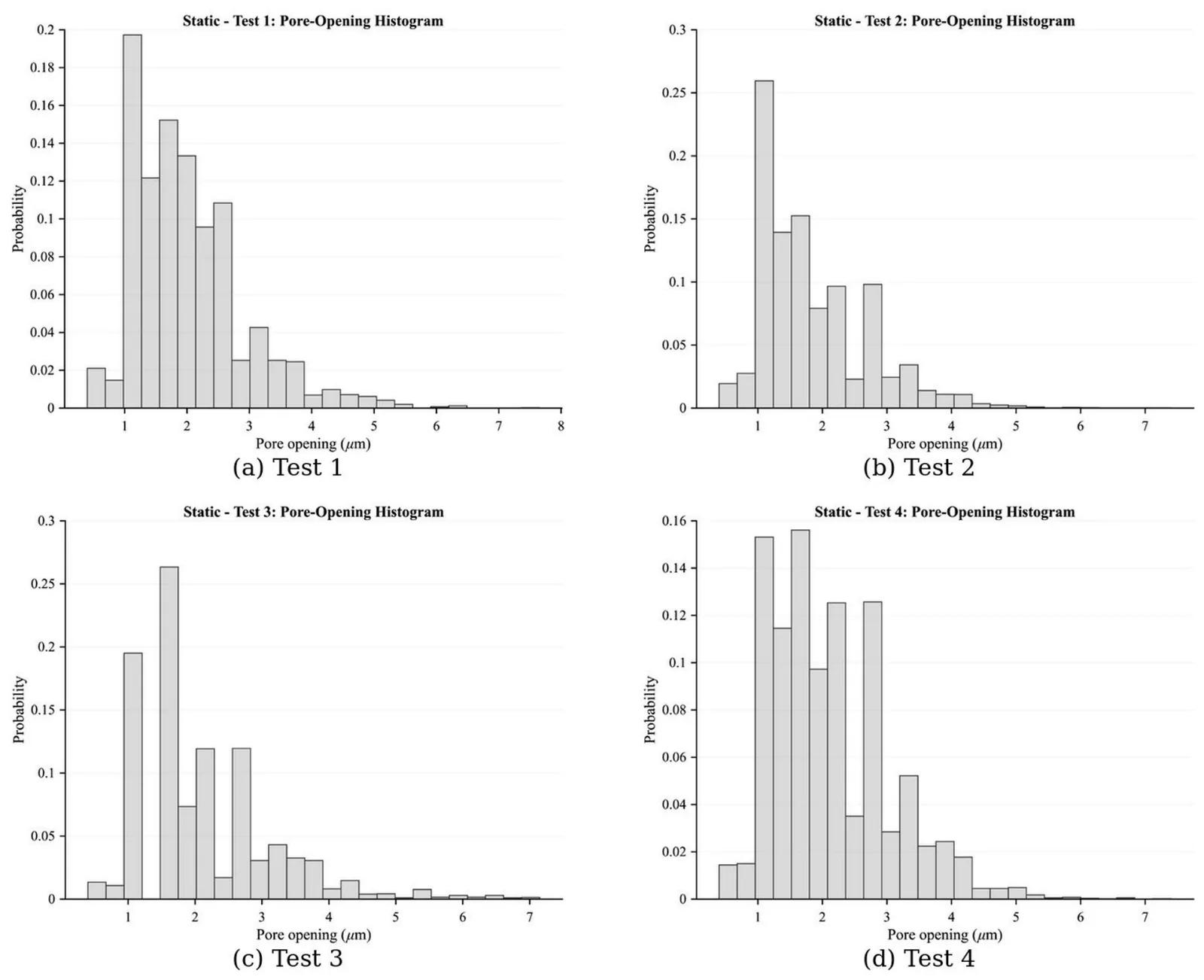
Static: pore-opening histograms for the four independent depositions (T1–T4).

**Figure A23:**
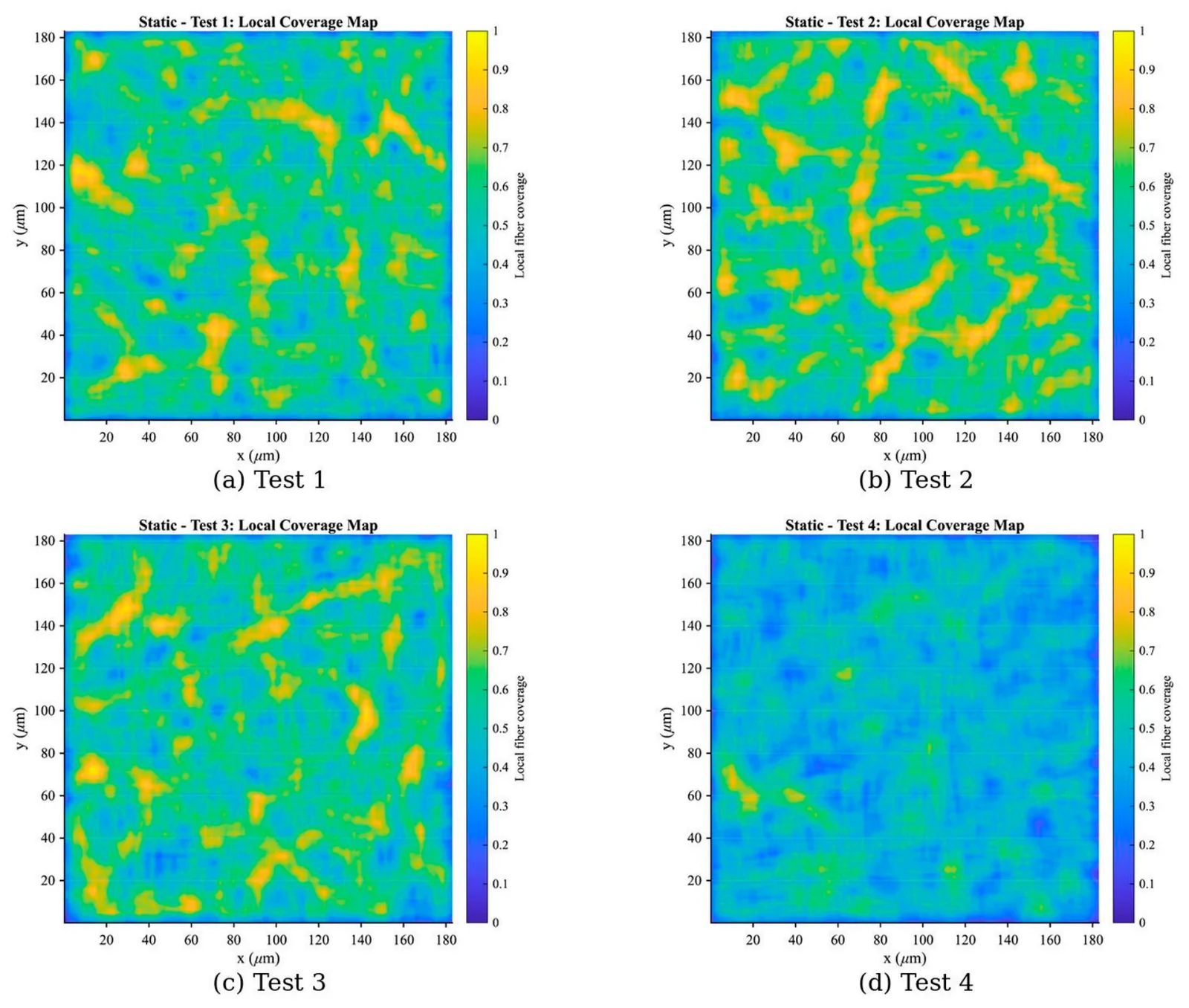
Static: local coverage maps for the four independent depositions (T1–T4).

**Figure A24:**
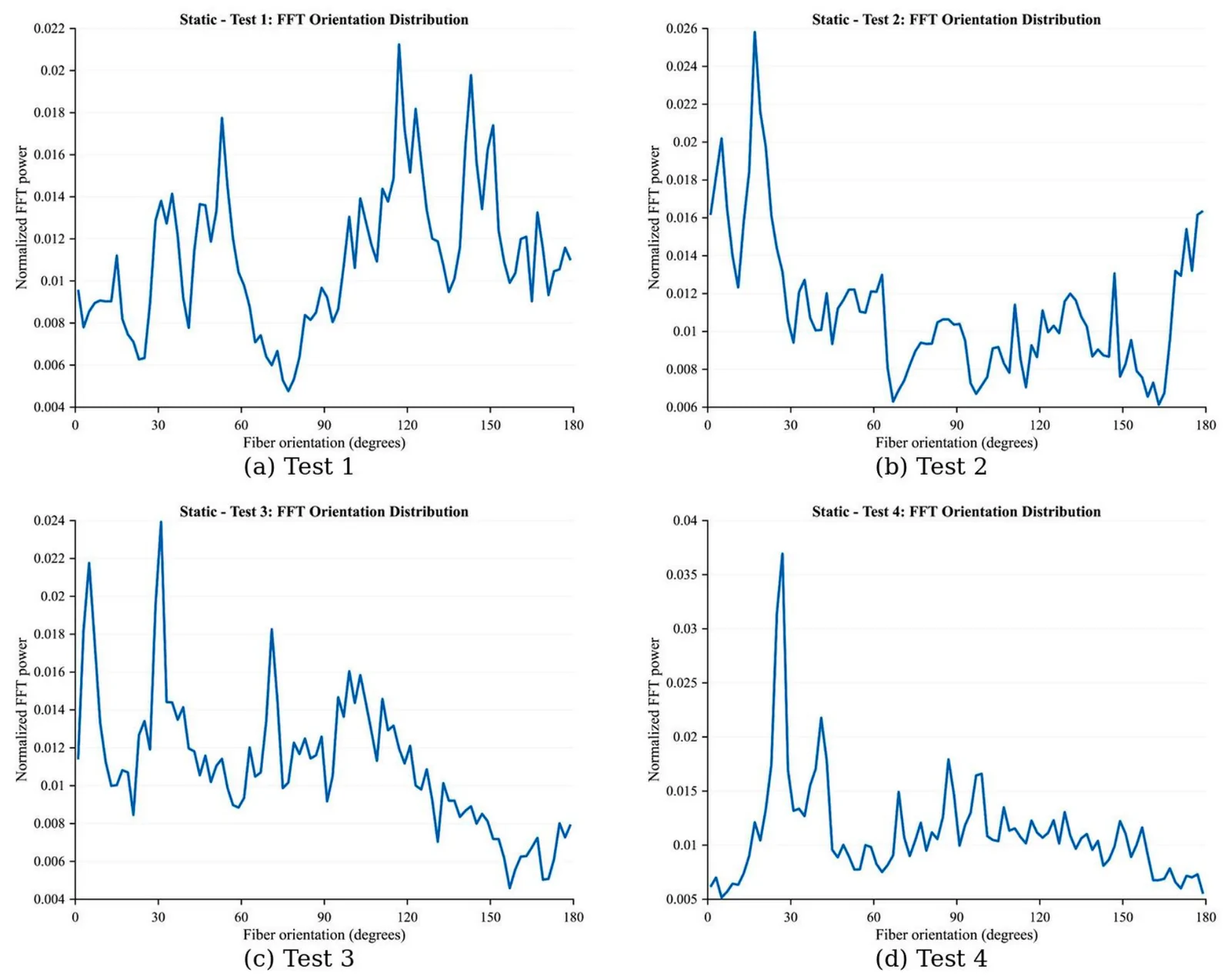
Static: FFT-based orientation distributions for the four independent depositions (T1–T4).

**Figure A25:**
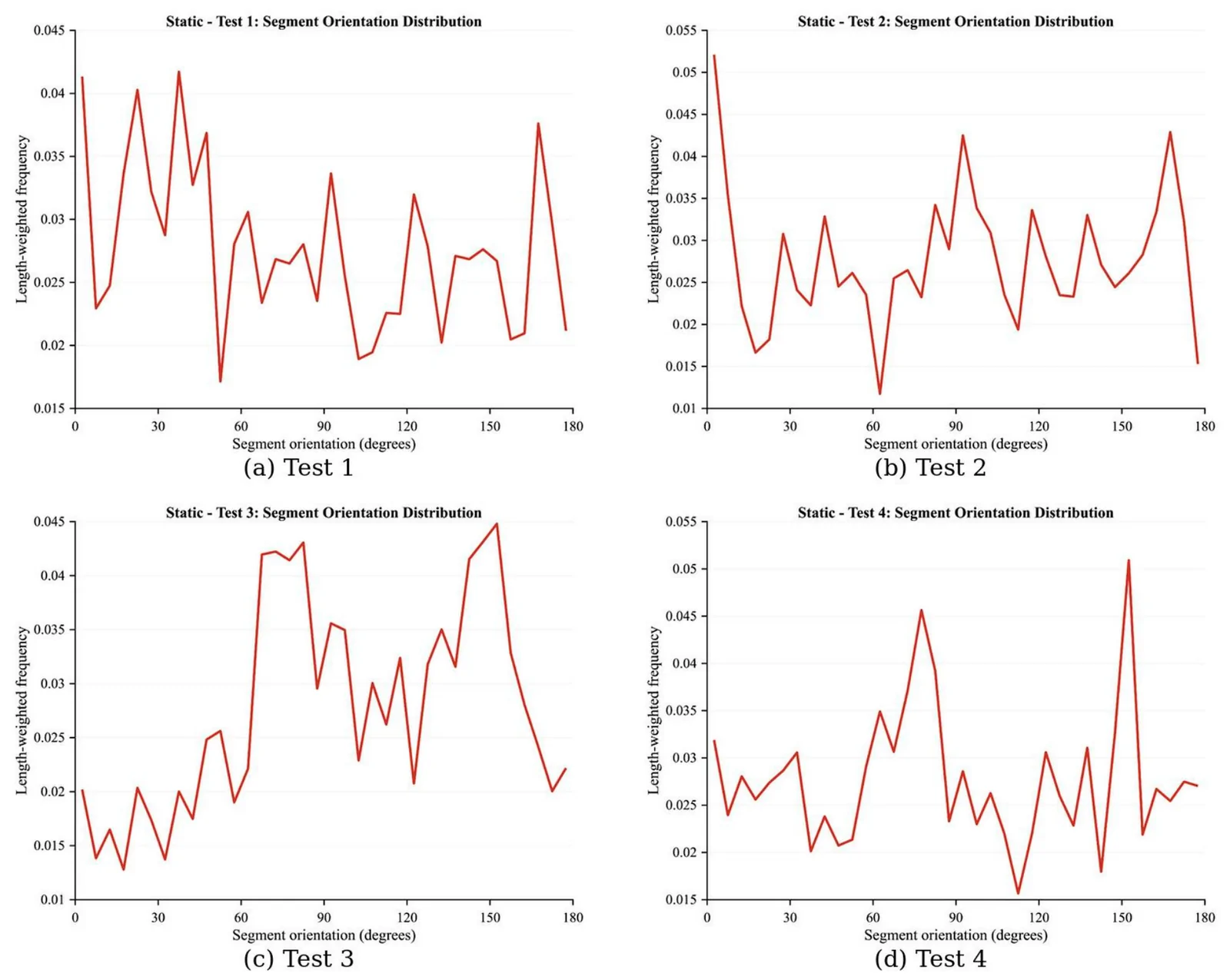
Static: skeleton-segment orientation distributions for the four independent depositions (T1–T4).

**Figure A26:**
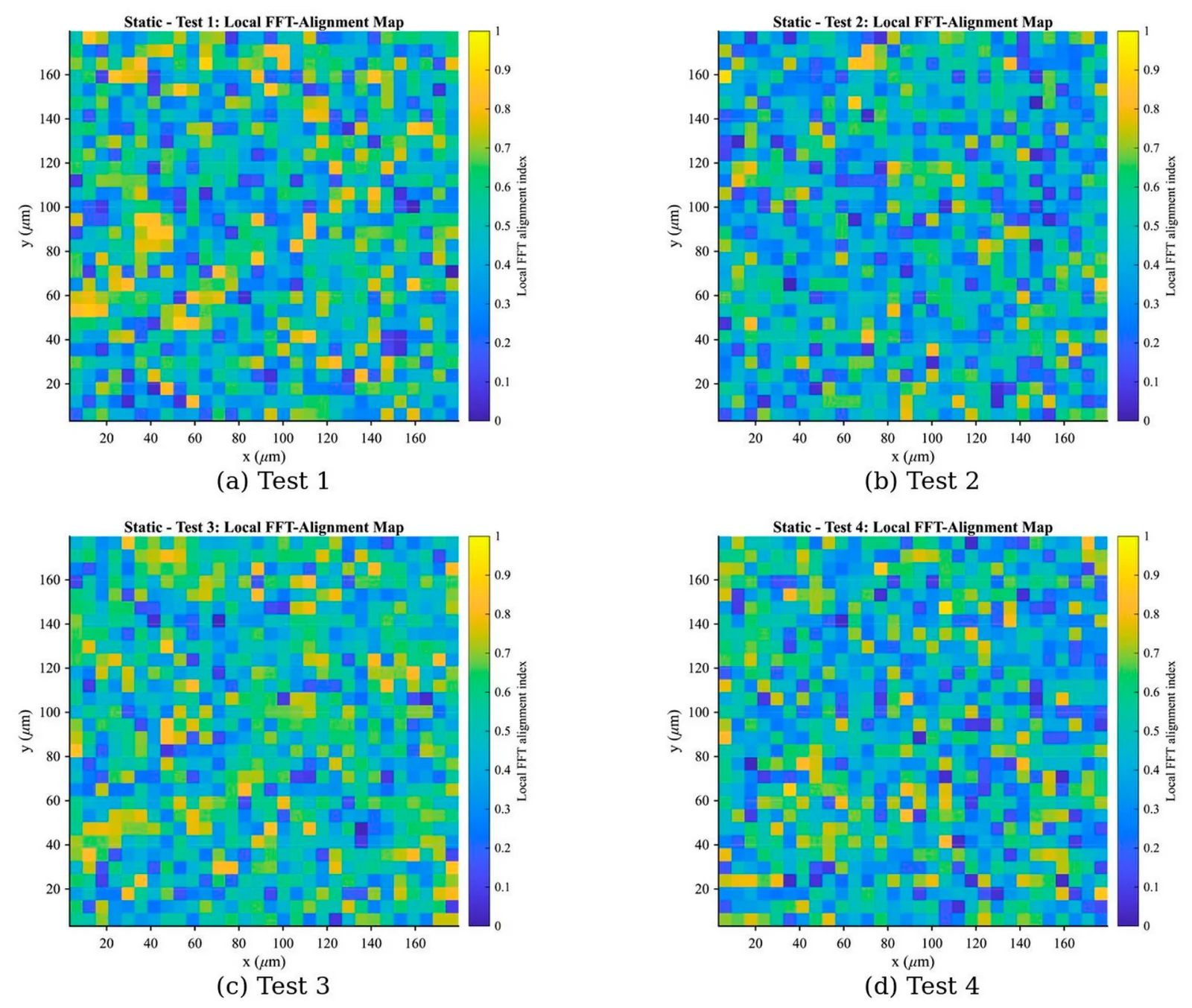
Static: local FFT-alignment maps for the four independent depositions (T1–T4).

**Figure A27:**
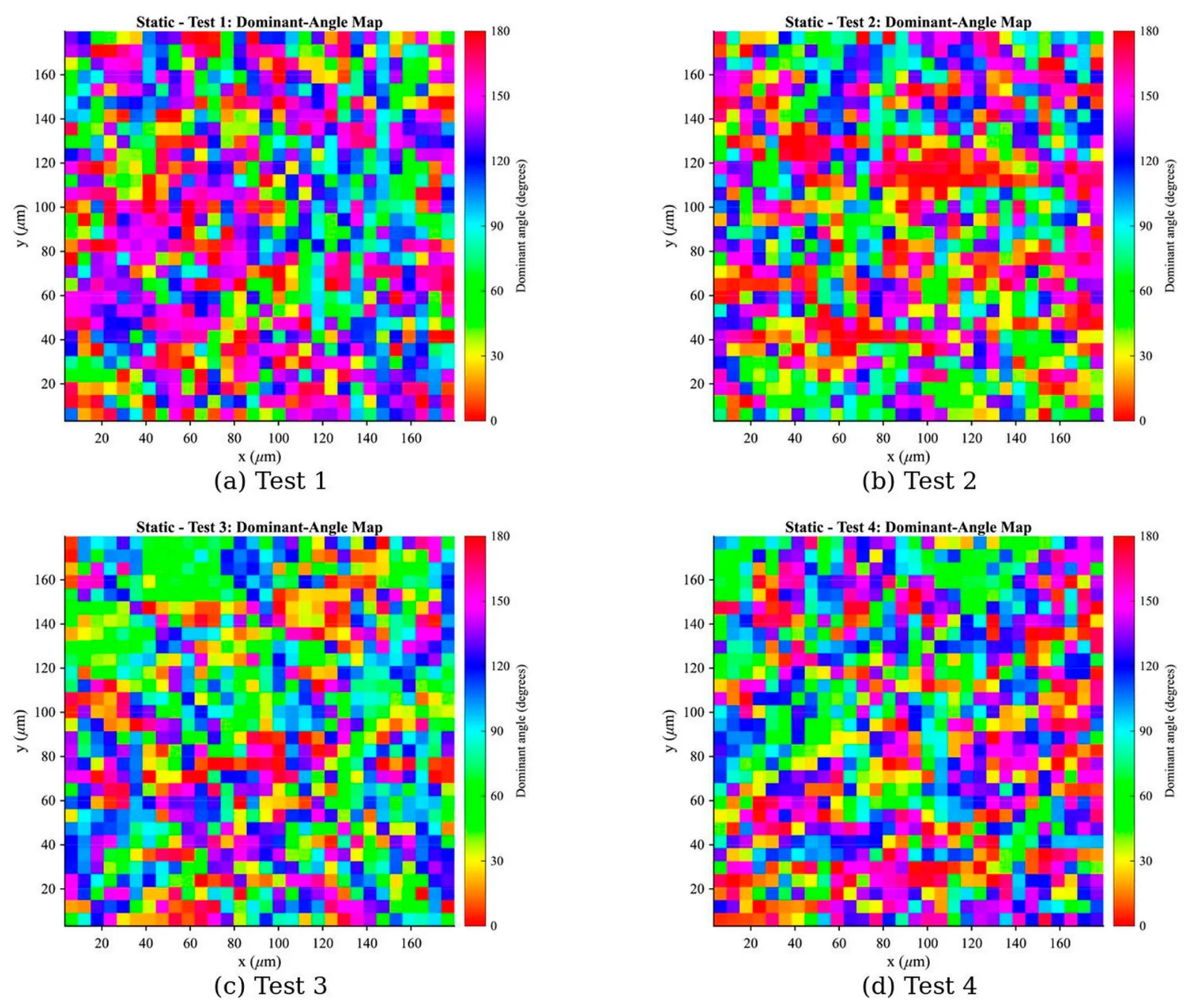
Static: dominant-angle maps for the four independent depositions (T1–T4).

**Figure A28:**
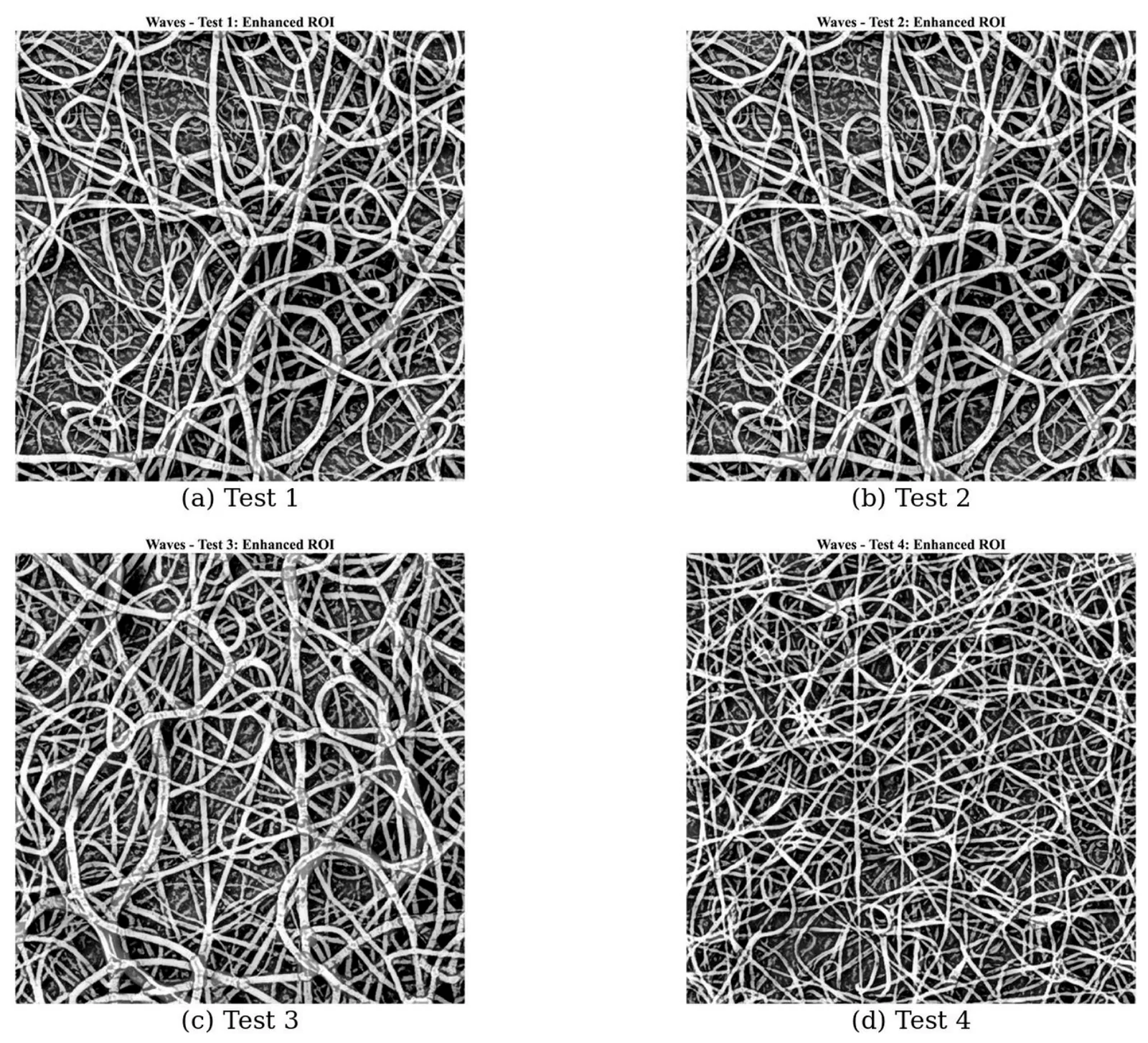
Waves: enhanced ROI images for the four independent depositions (T1–T4).

**Figure A29:**
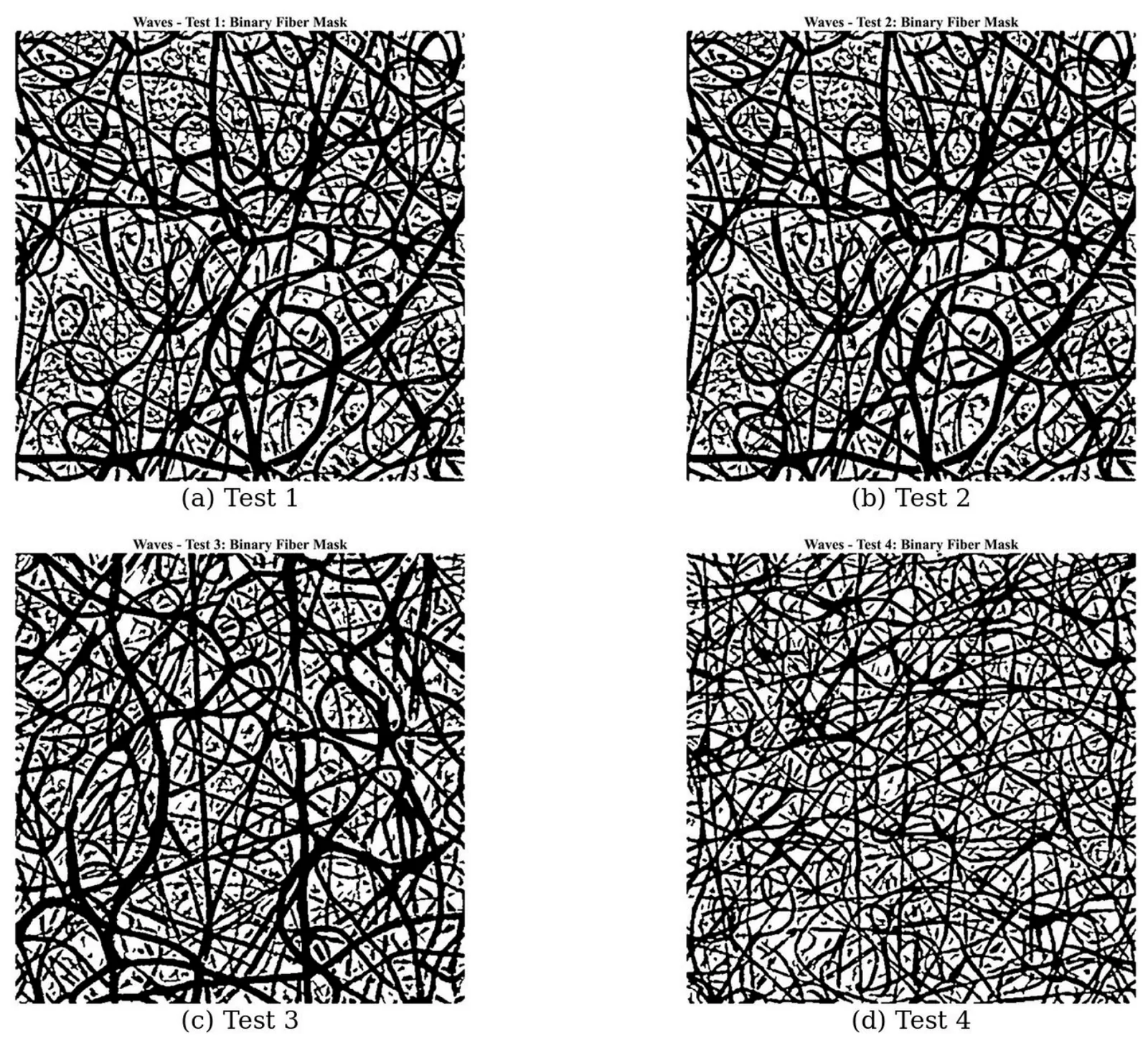
Waves: binary fiber masks for the four independent depositions (T1–T4).

**Figure A30:**
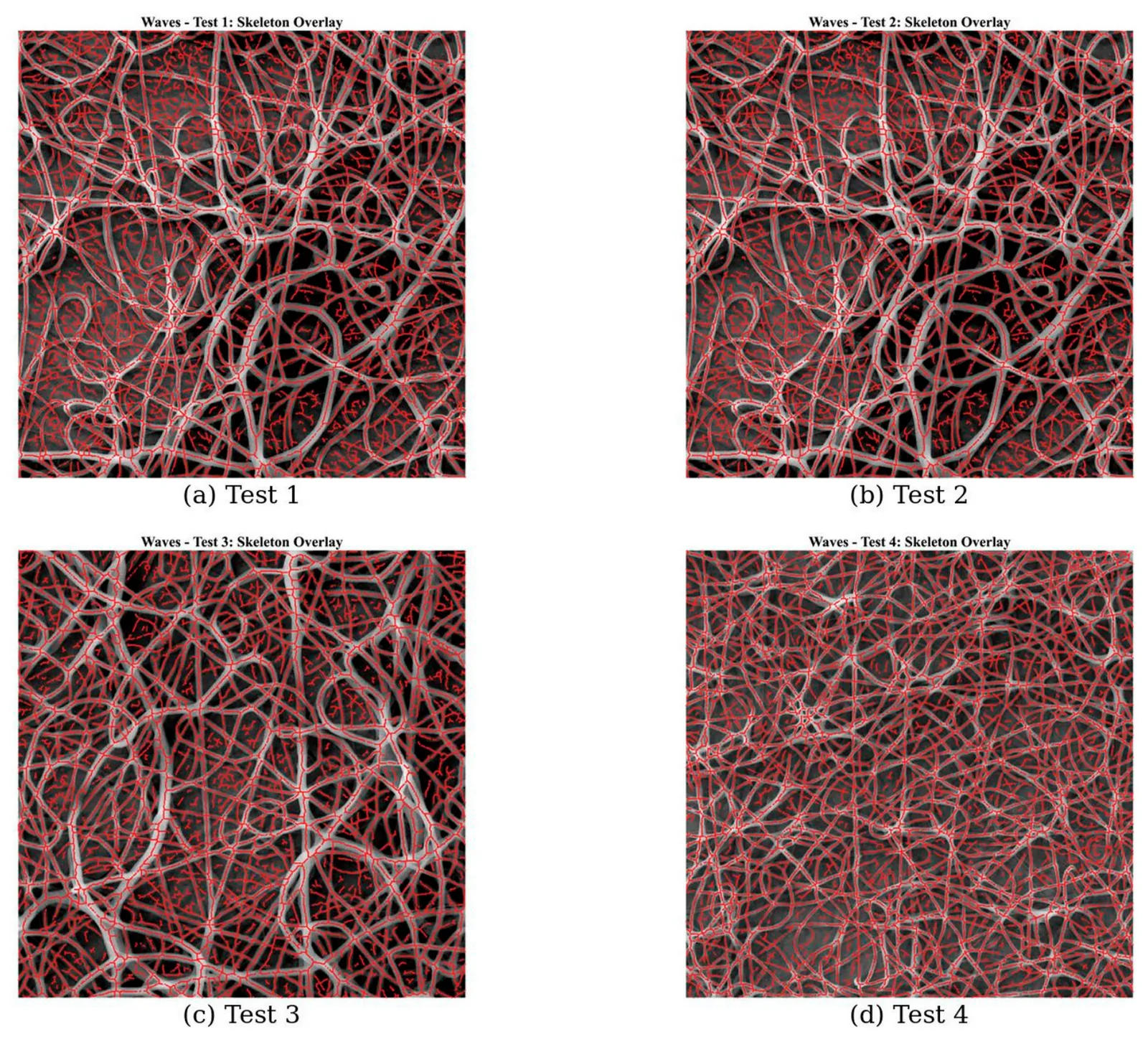
Waves: skeleton overlays for the four independent depositions (T1–T4).

**Figure A31:**
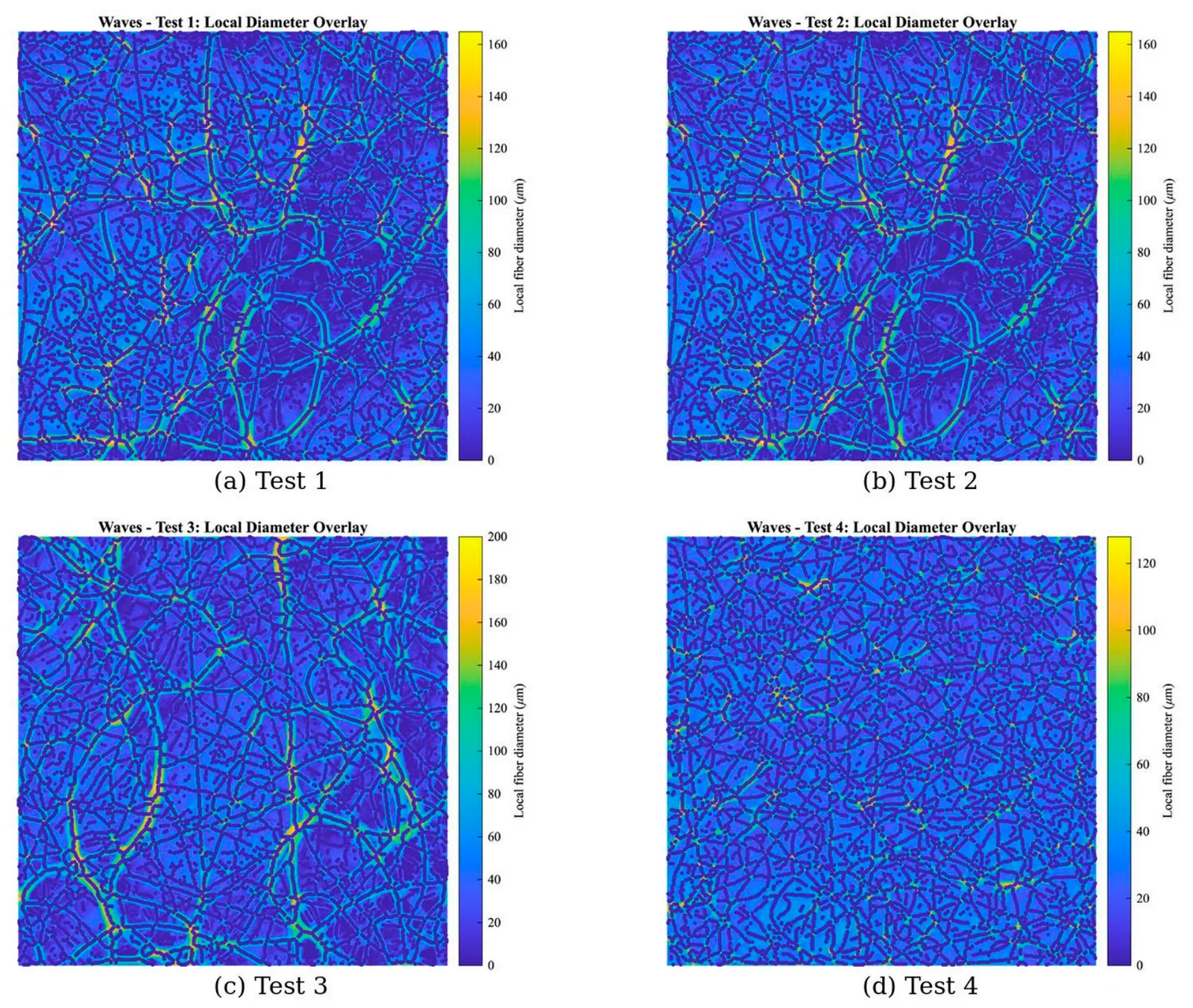
Waves: local diameter overlays for the four independent depositions (T1–T4).

**Figure A32:**
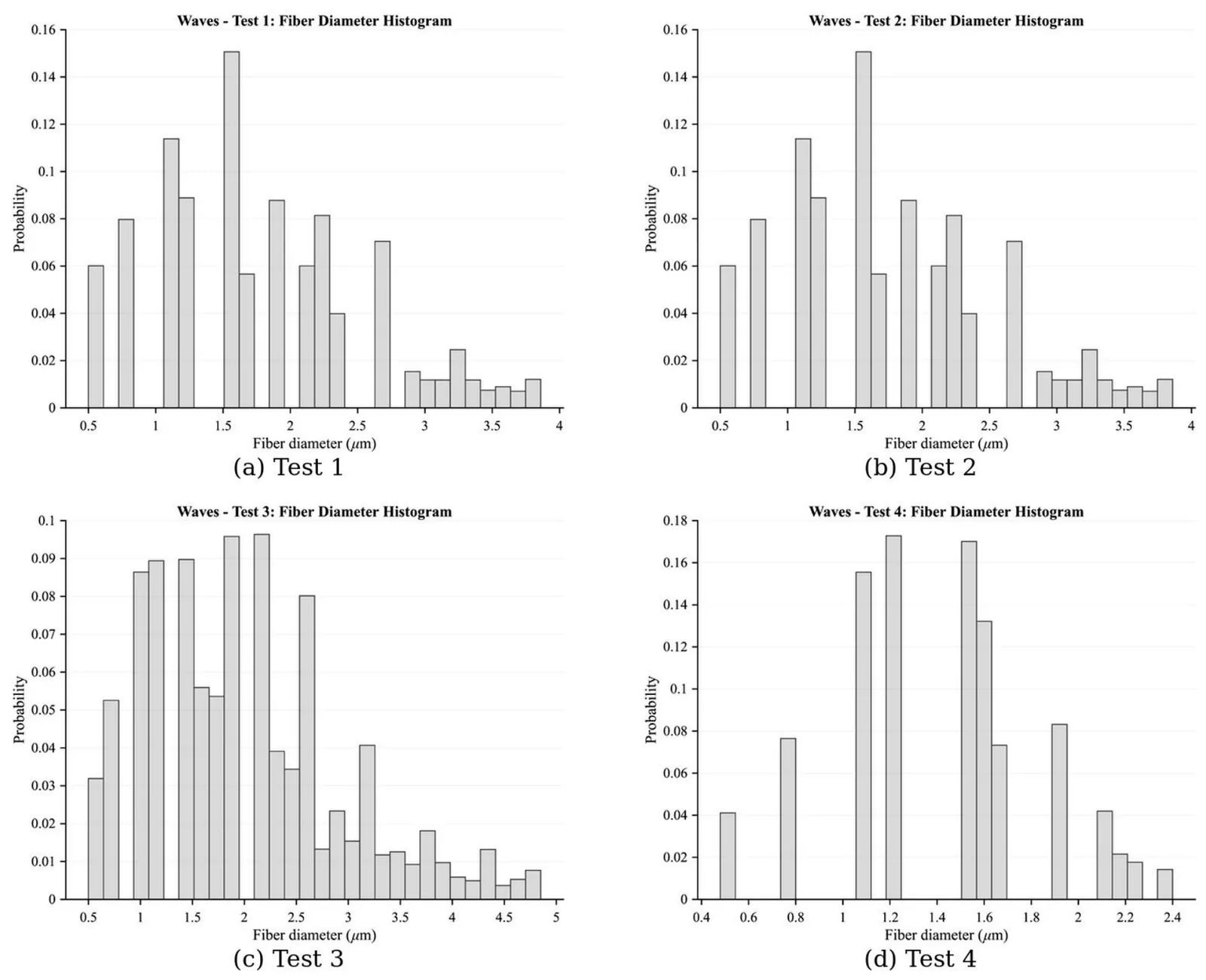
Waves: fiber-diameter histograms for the four independent depositions (T1–T4).

**Figure A33:**
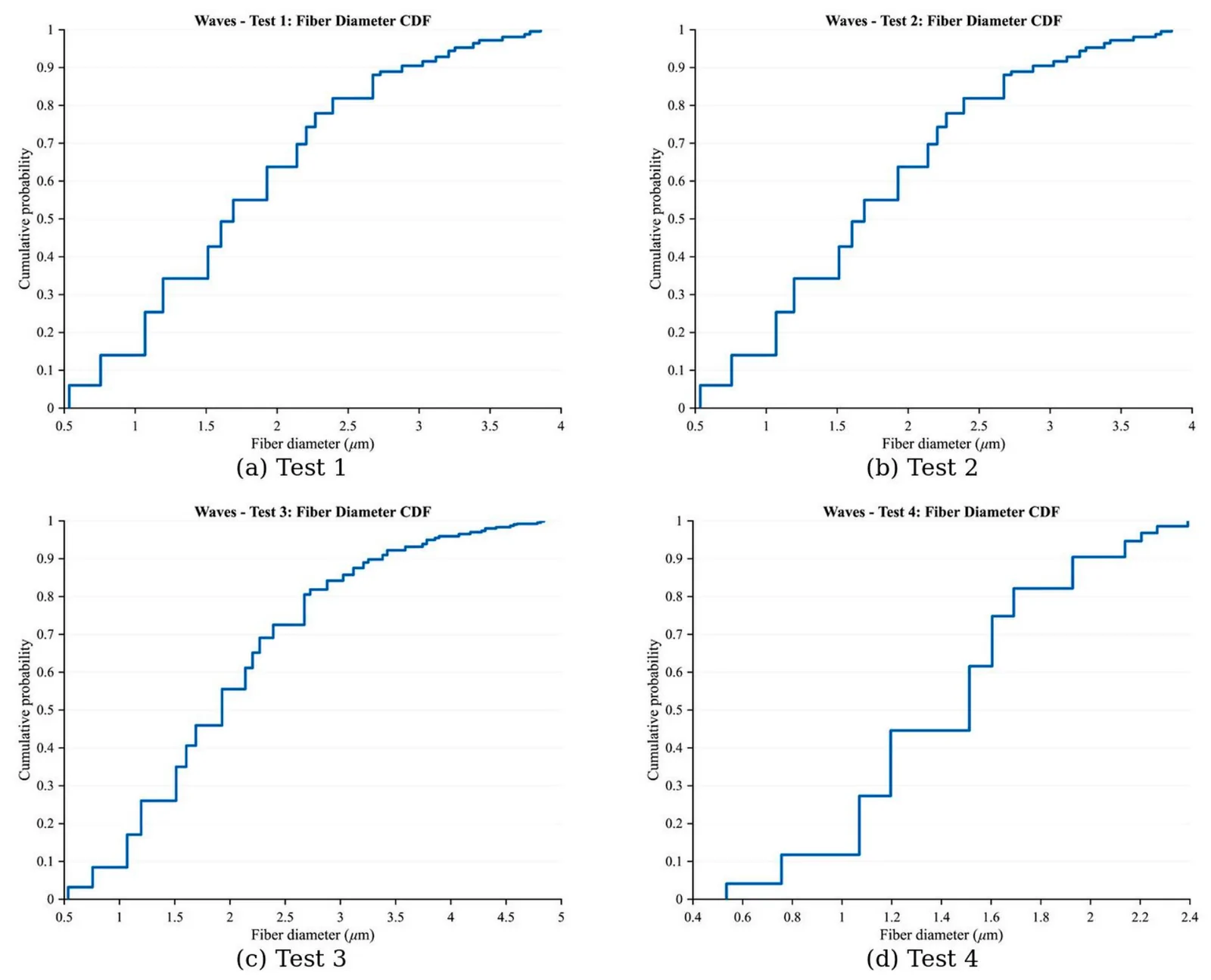
Waves: fiber-diameter cumulative distributions for the four independent depositions (T1–T4).

**Figure A34:**
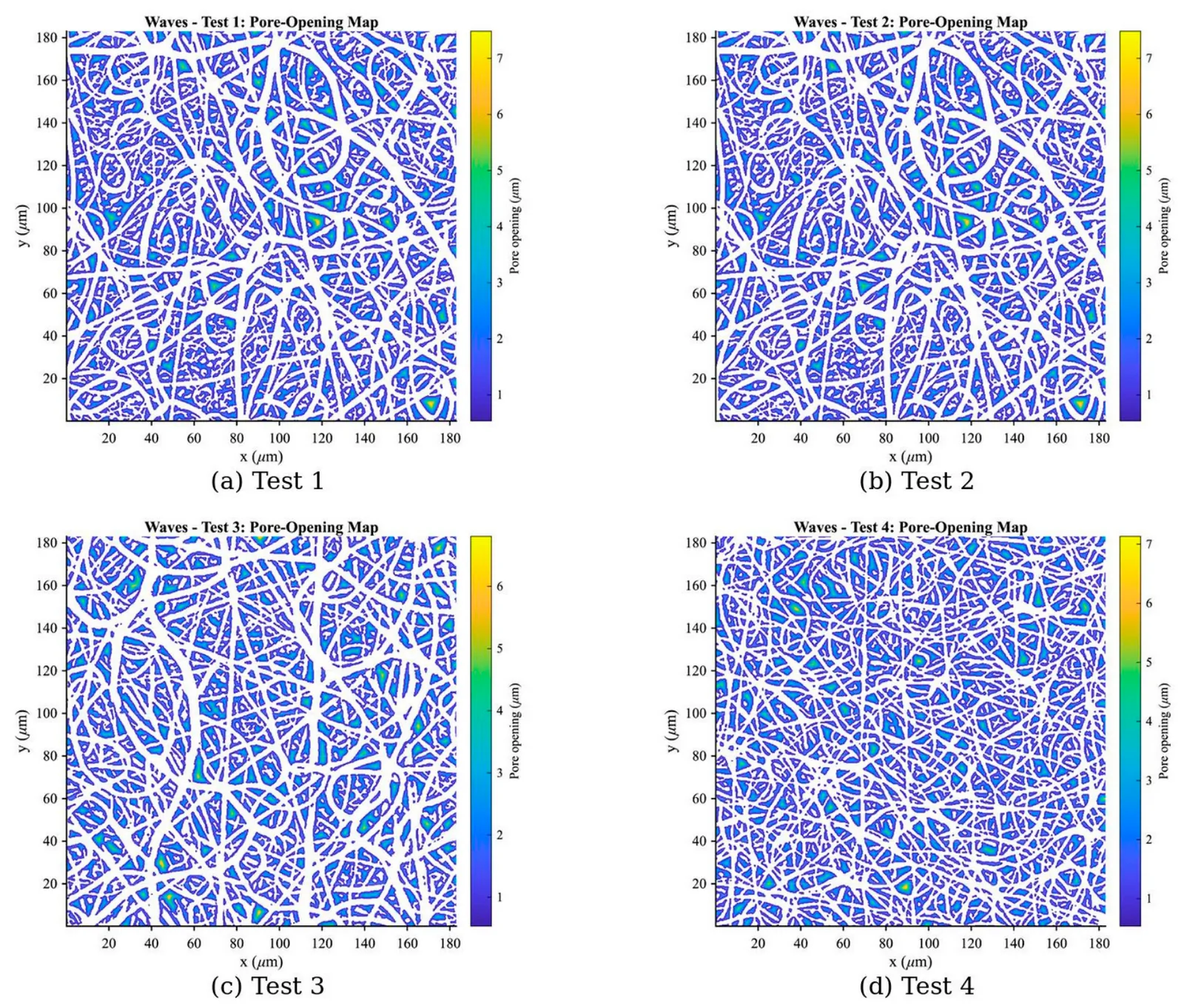
Waves: local pore-opening maps for the four independent depositions (T1–T4).

**Figure A35:**
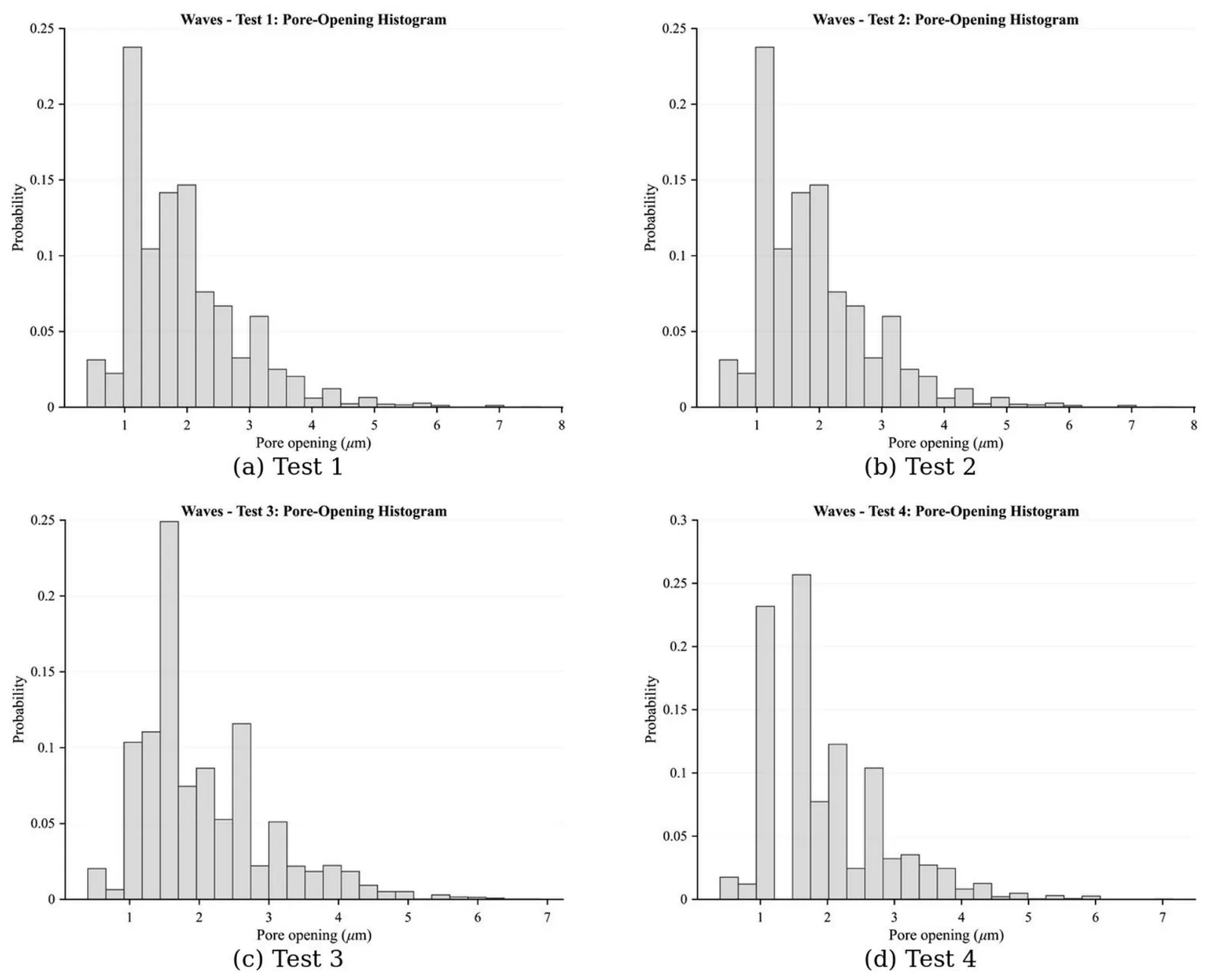
Waves: pore-opening histograms for the four independent depositions (T1–T4).

**Figure A36:**
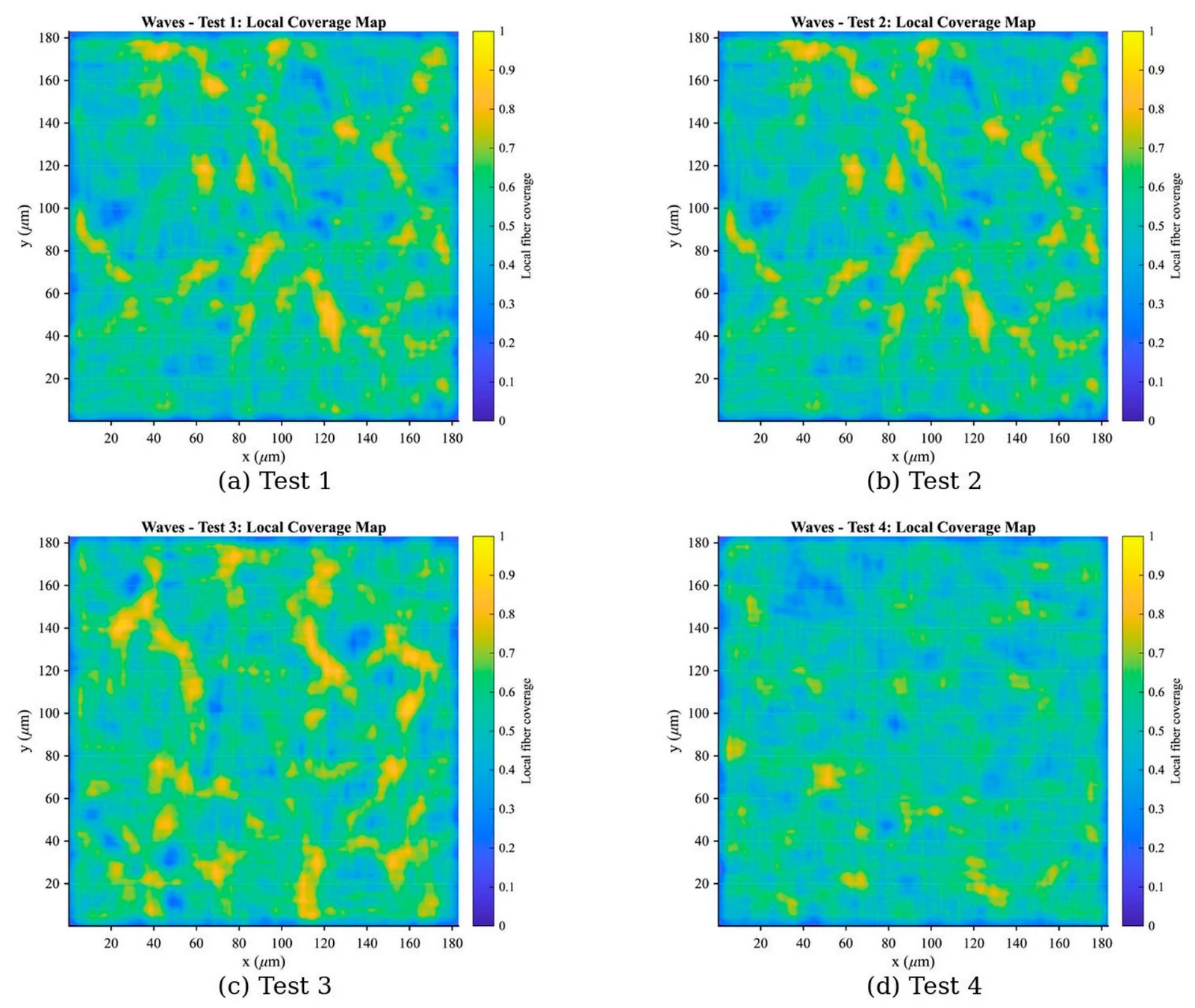
Waves: local coverage maps for the four independent depositions (T1–T4).

**Figure A37:**
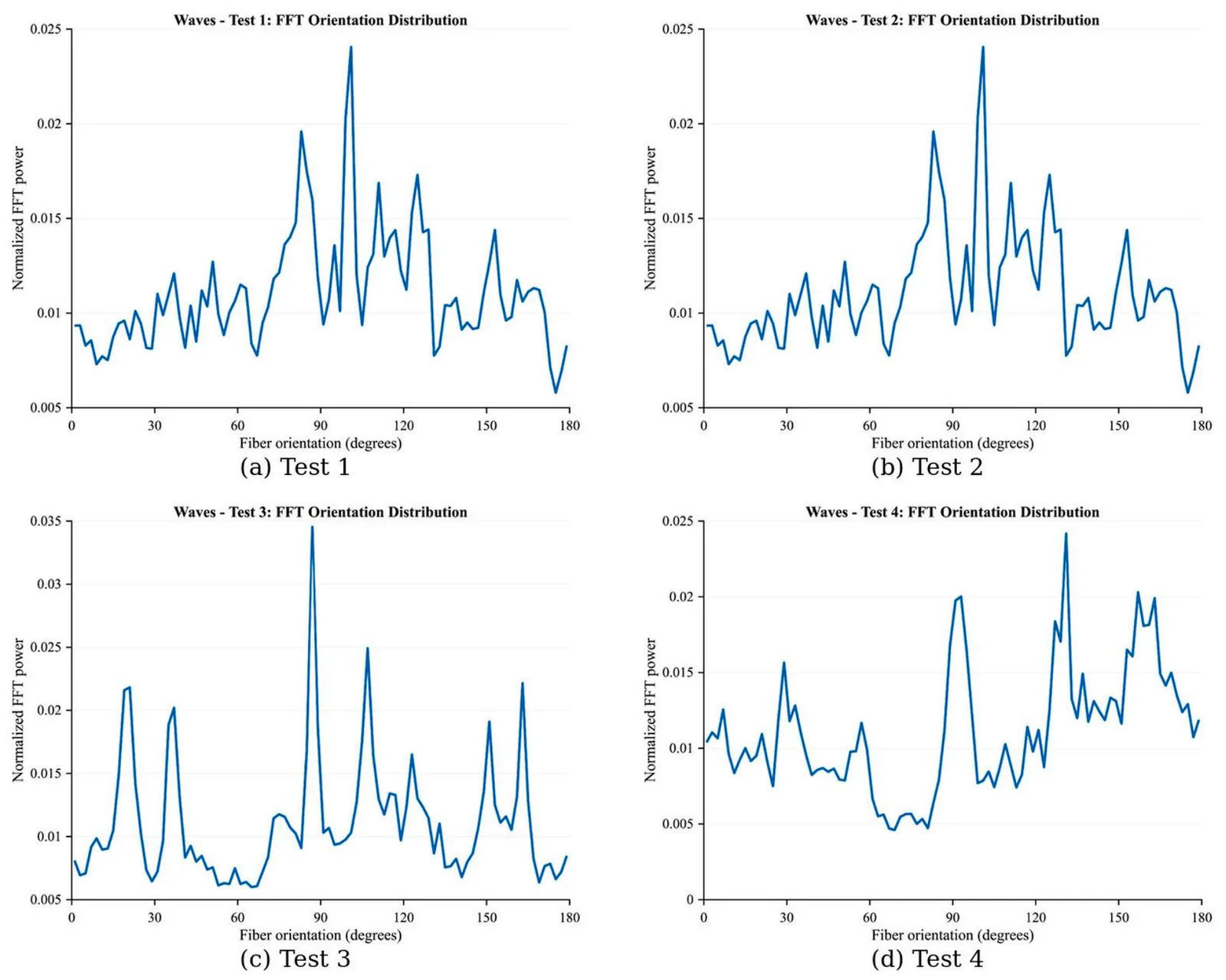
Waves: FFT-based orientation distributions for the four independent depositions (T1–T4).

**Figure A38:**
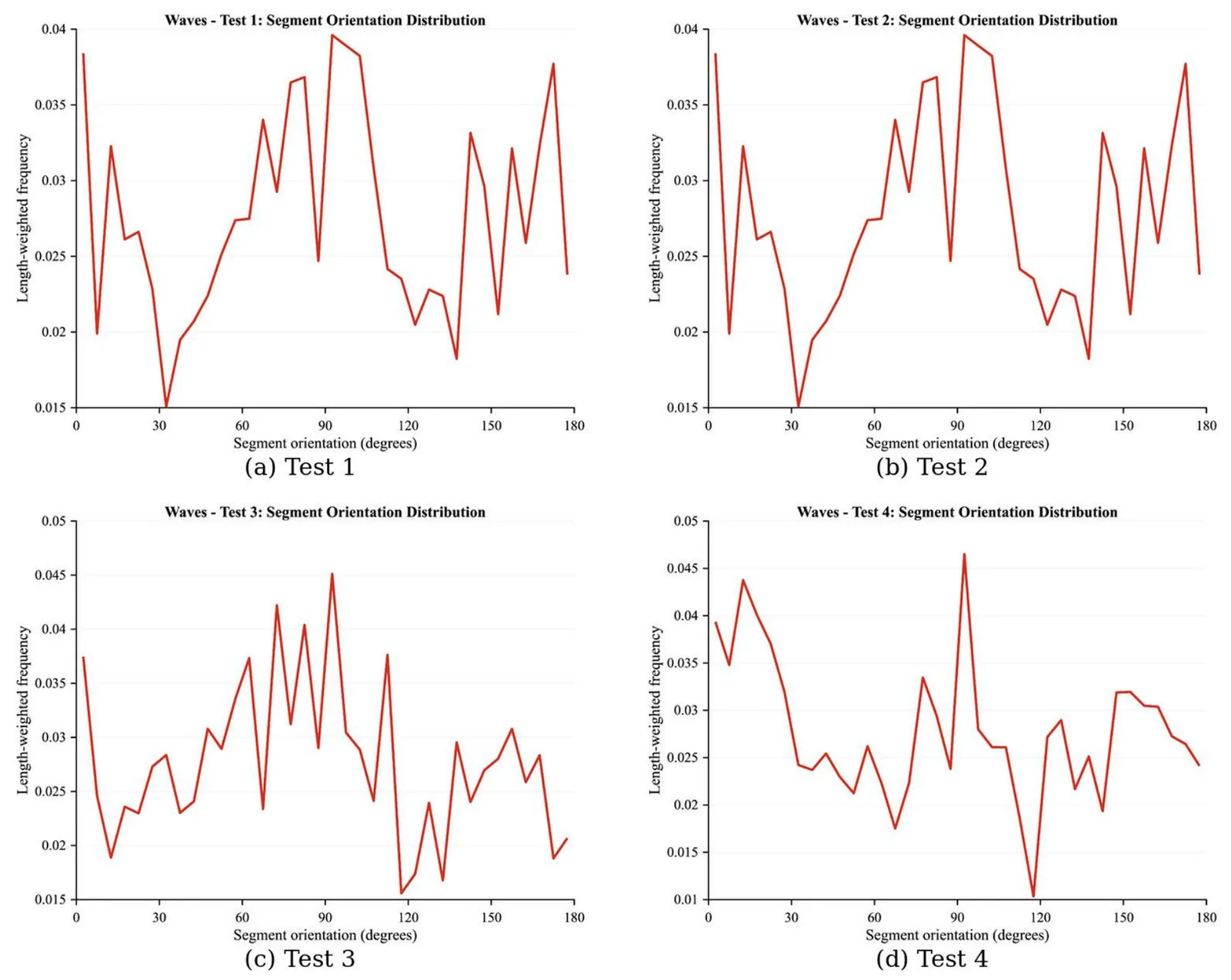
Waves: skeleton-segment orientation distributions for the four independent depositions (T1–T4).

**Figure A39:**
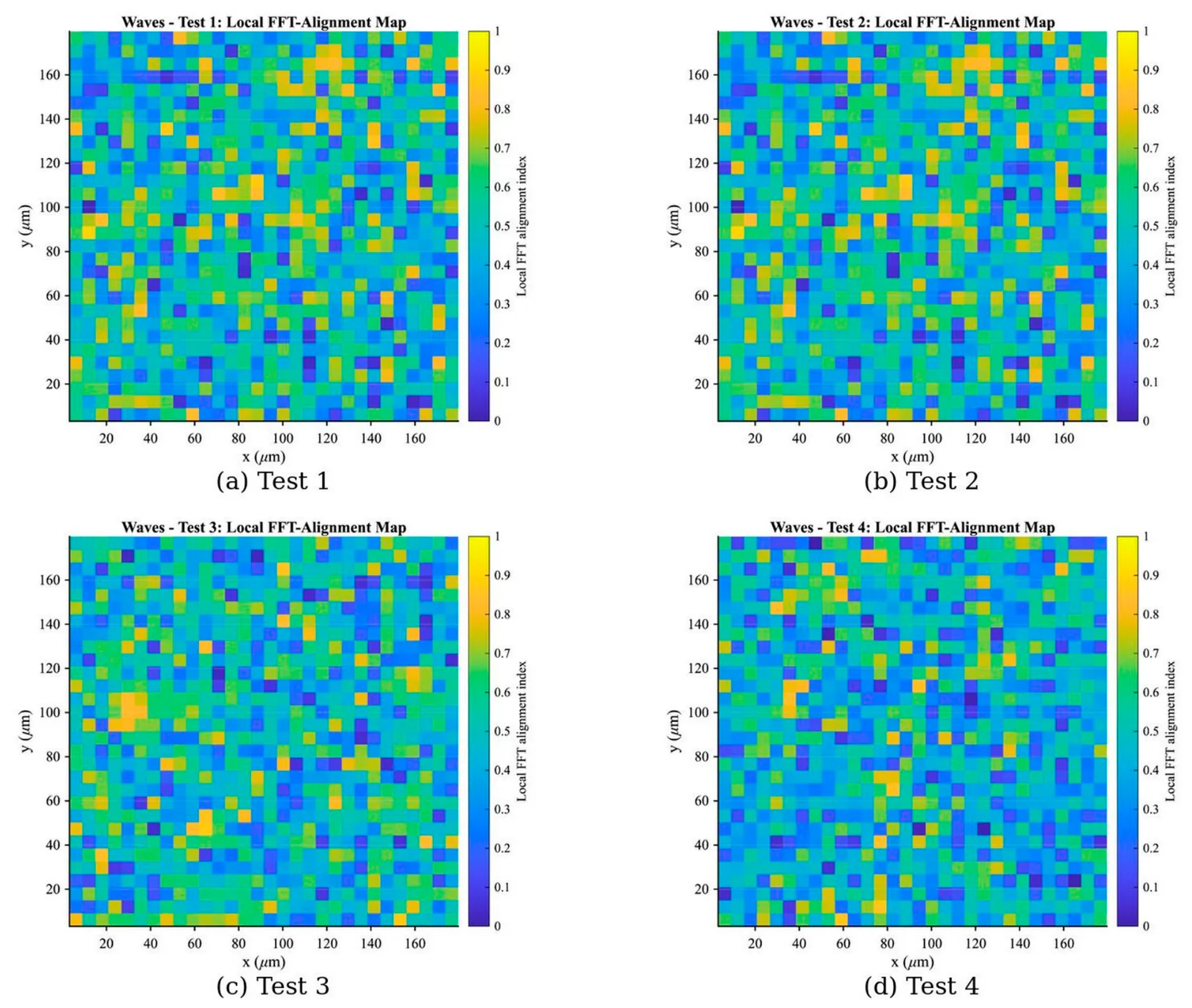
Waves: local FFT-alignment maps for the four independent depositions (T1–T4).

**Figure A40:**
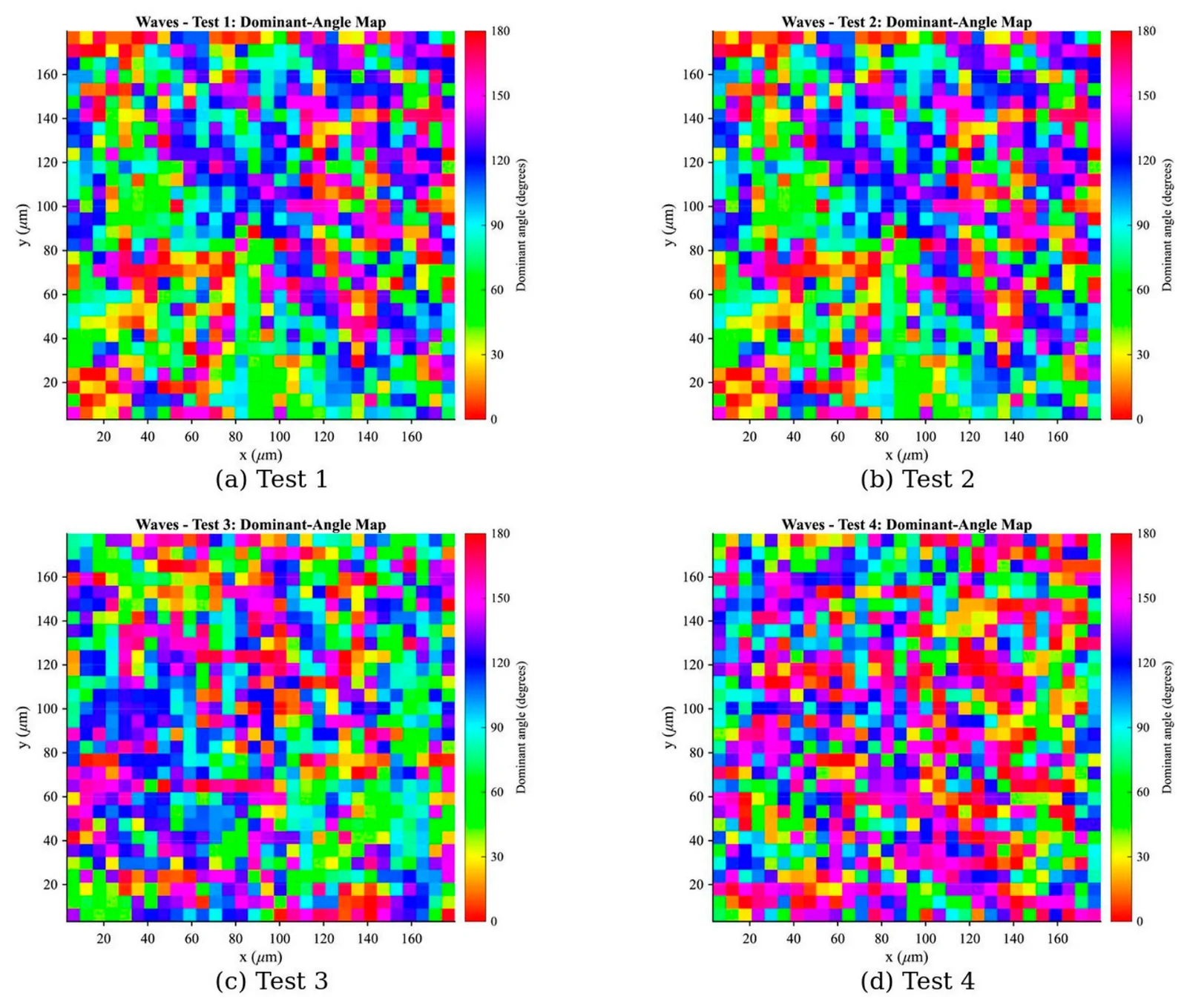
Waves: dominant-angle maps for the four independent depositions (T1–T4).

**Figure A41:**
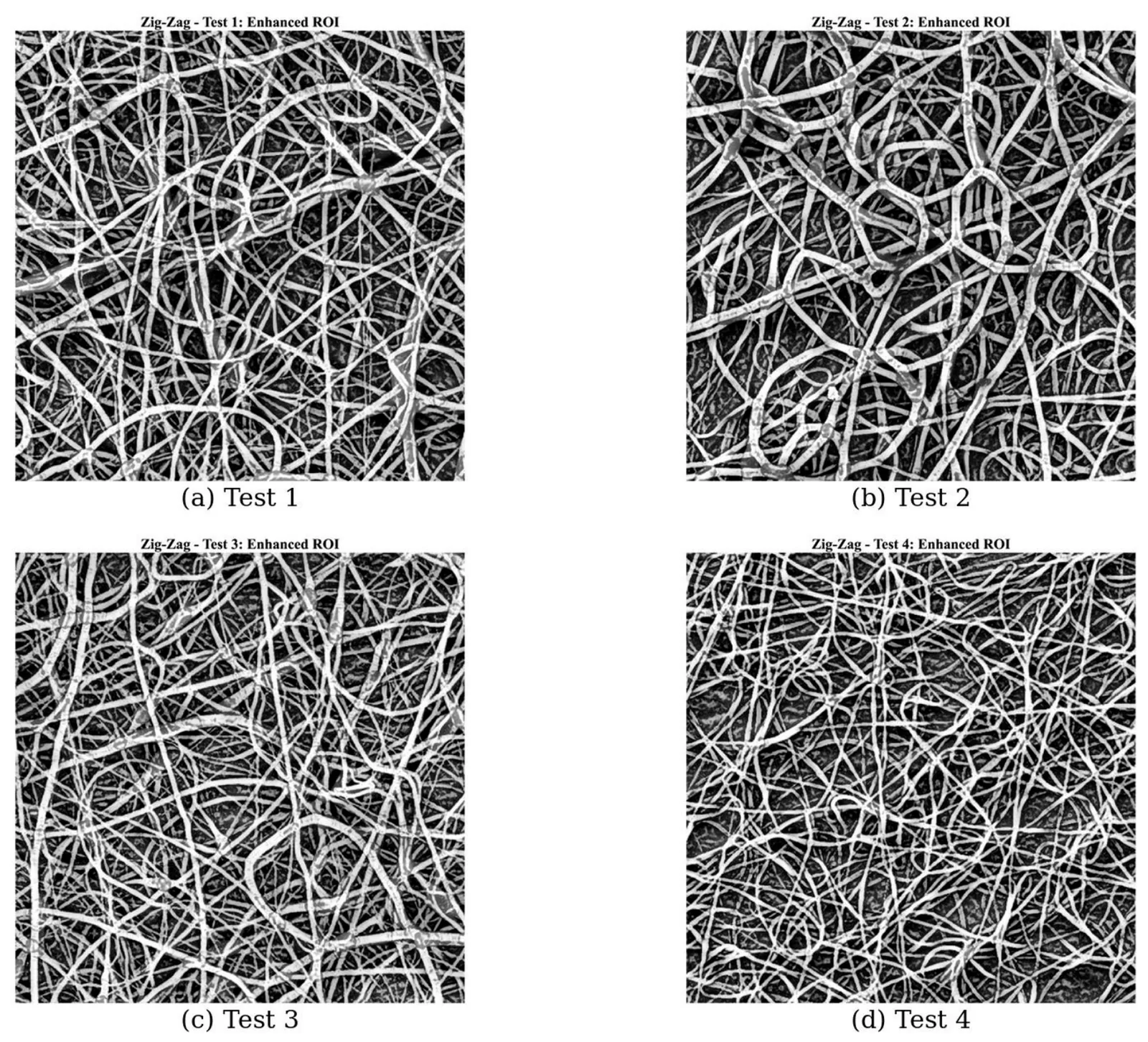
Zig-Zag: enhanced ROI images for the four independent depositions (T1–T4).

**Figure A42:**
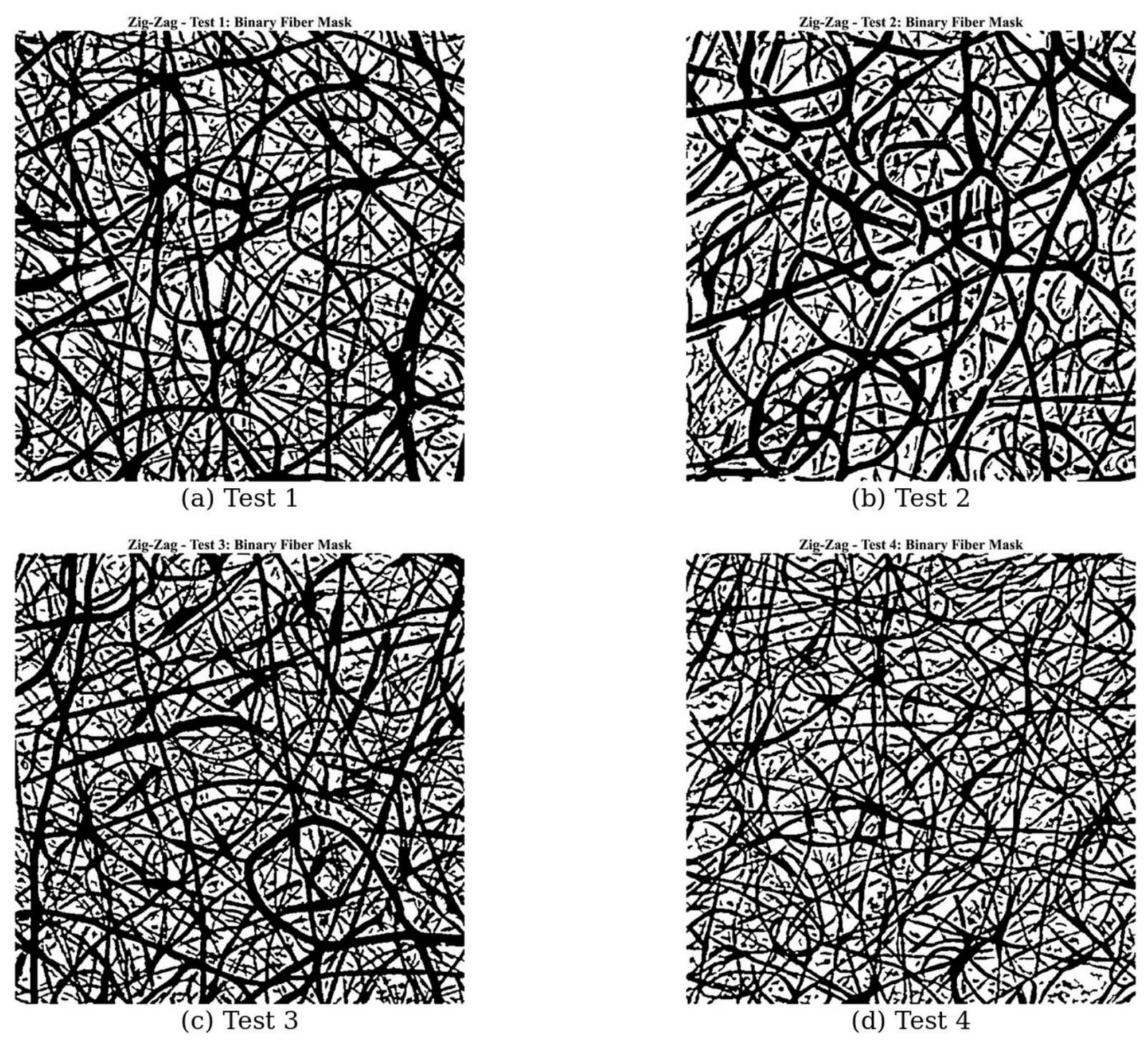
Zig-Zag: binary fiber masks for the four independent depositions (T1–T4).

**Figure A43:**
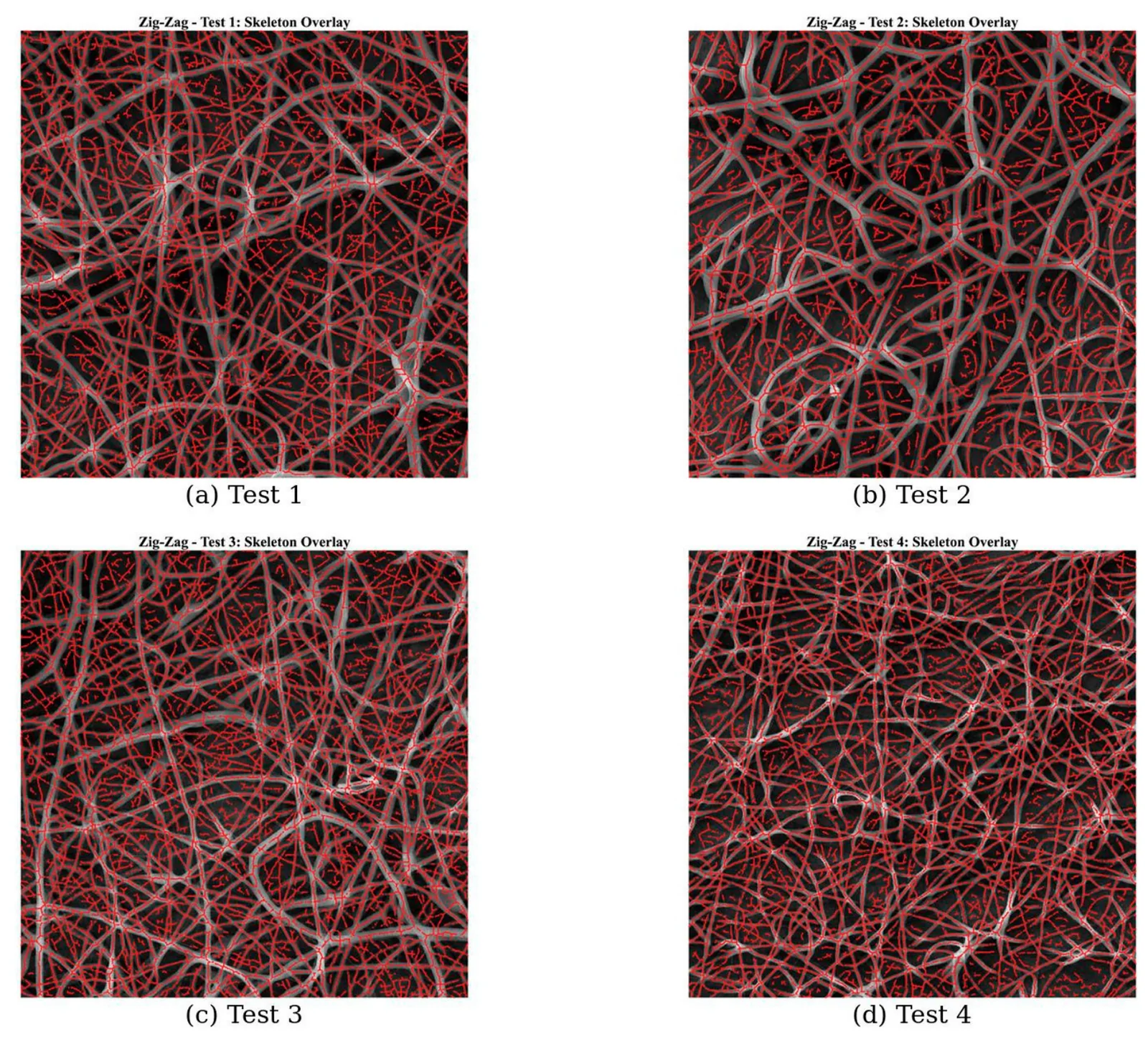
Zig-Zag: skeleton overlays for the four independent depositions (T1–T4).

**Figure A44:**
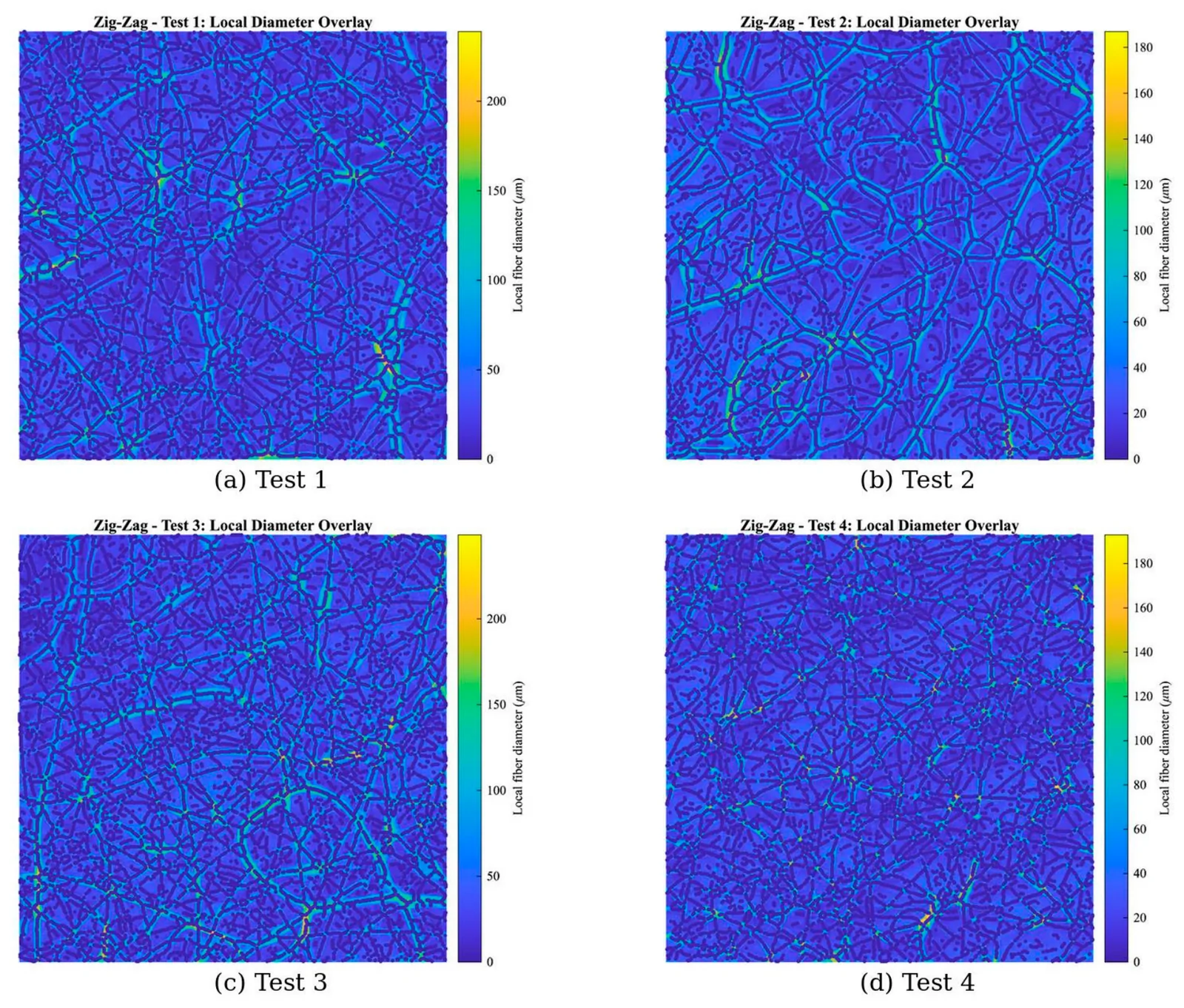
Zig-Zag: local diameter overlays for the four independent depositions (T1–T4).

**Figure A45:**
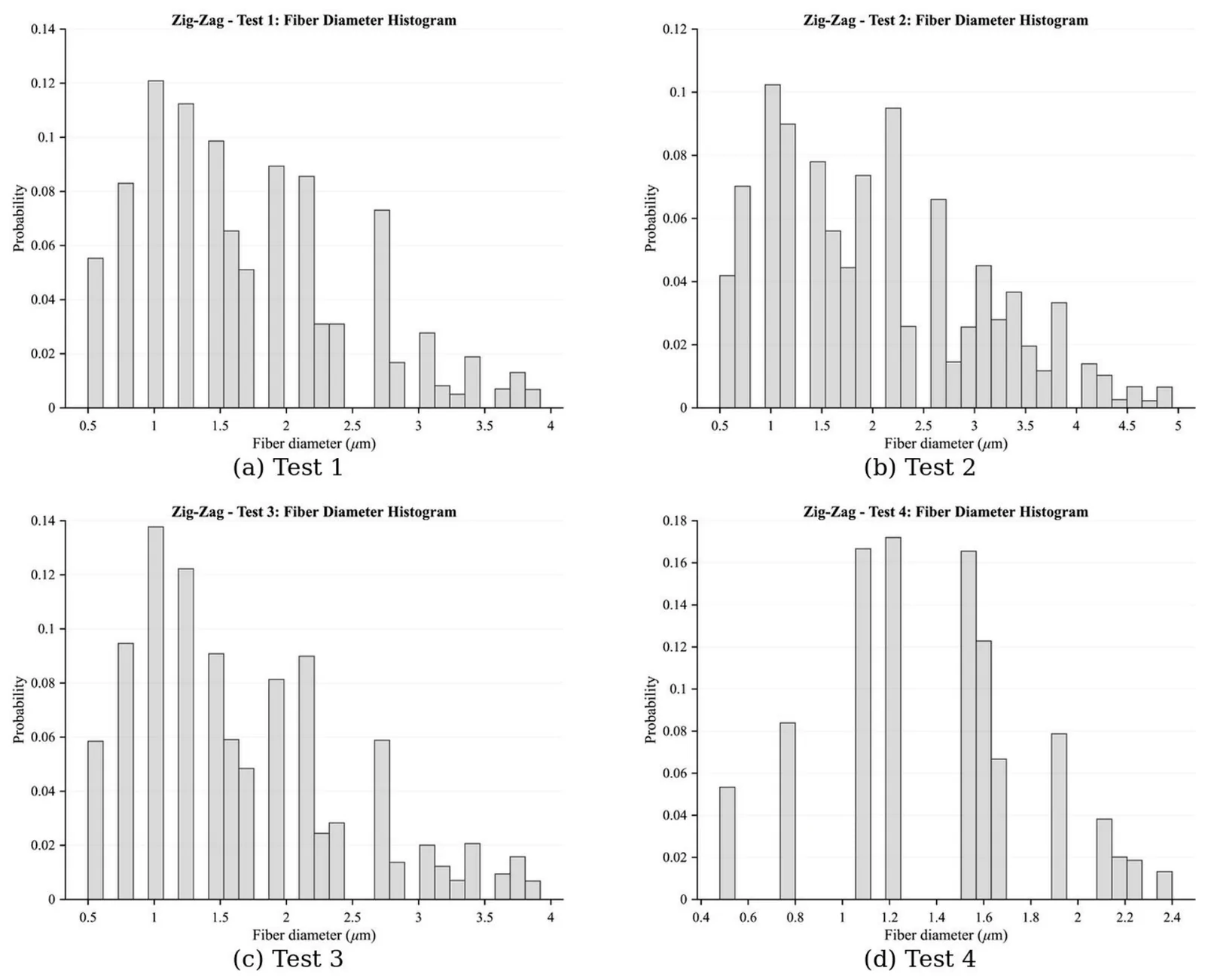
Zig-Zag: fiber-diameter histograms for the four independent depositions (T1–T4).

**Figure A46:**
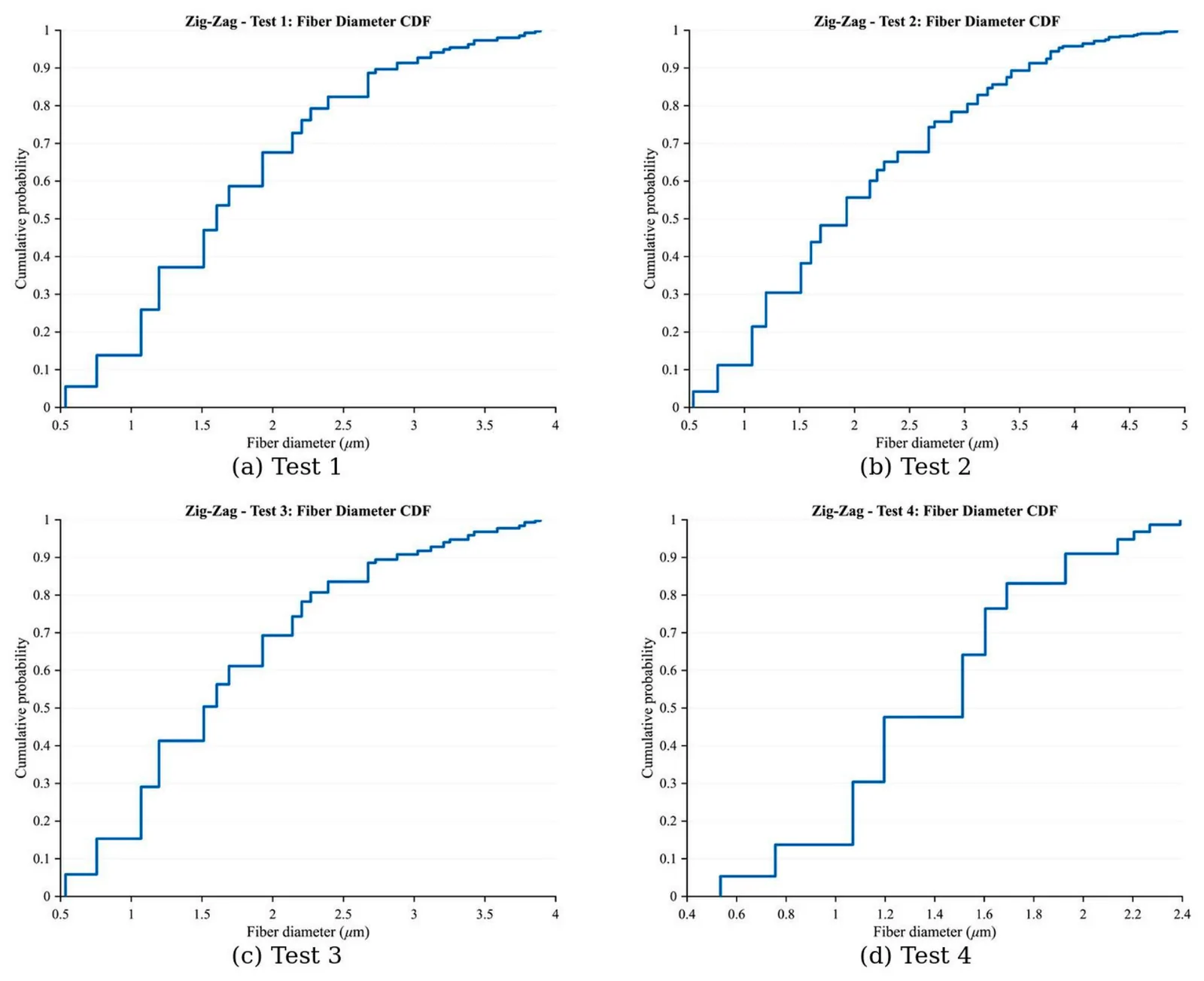
Zig-Zag: fiber-diameter cumulative distributions for the four independent depositions (T1–T4).

**Figure A47:**
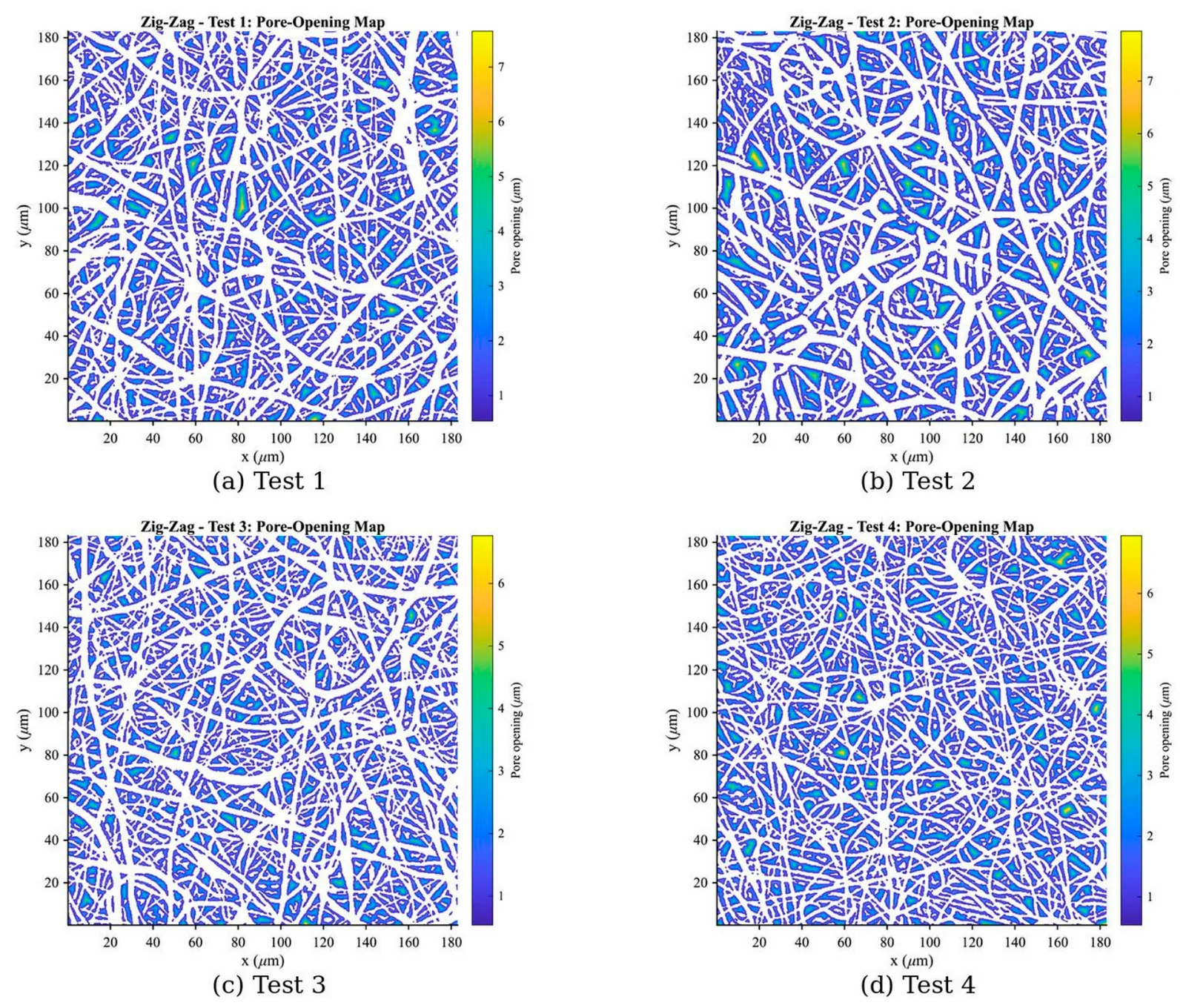
Zig-Zag: local pore-opening maps for the four independent depositions (T1–T4).

**Figure A48:**
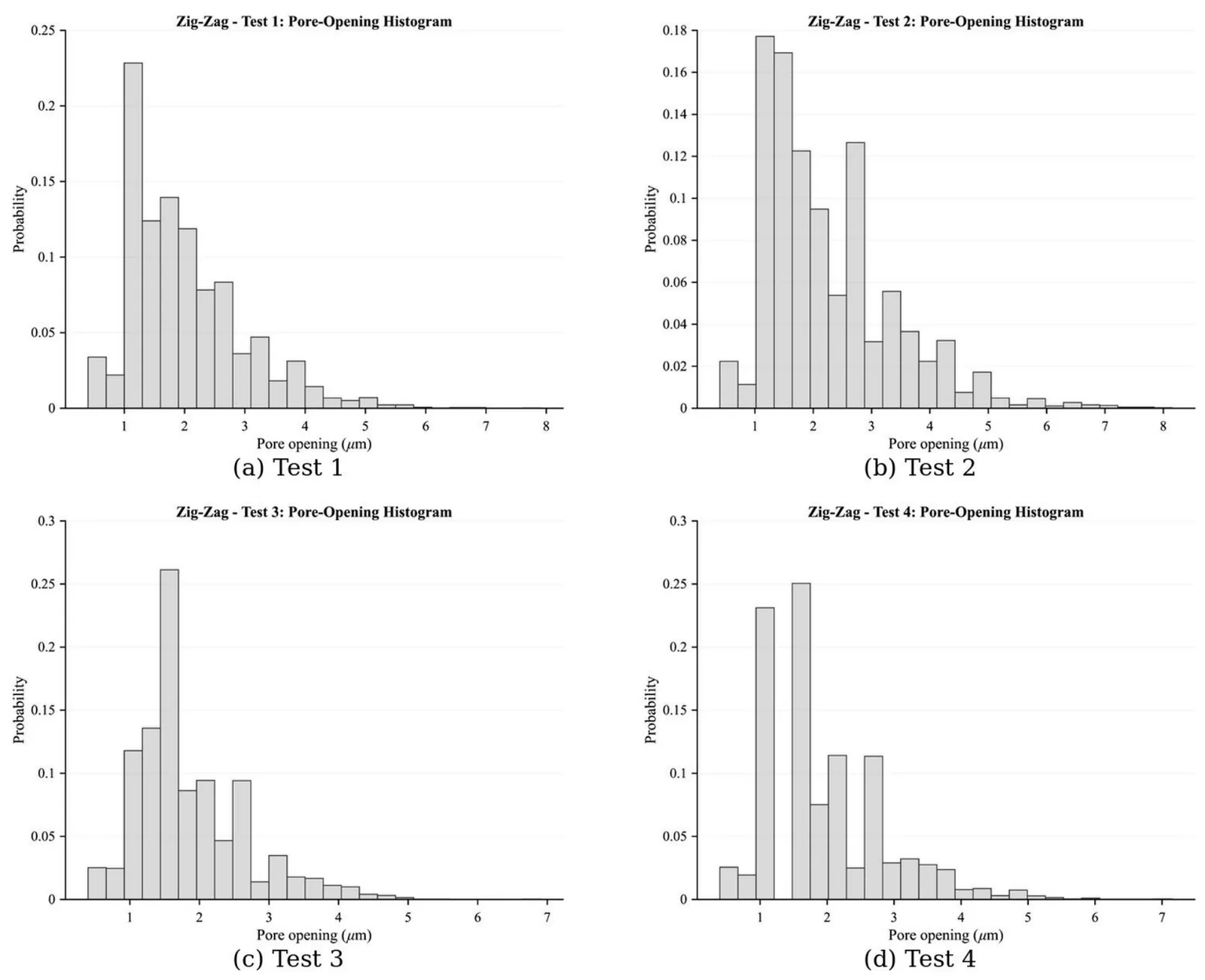
Zig-Zag: pore-opening histograms for the four independent depositions (T1–T4).

**Figure A49:**
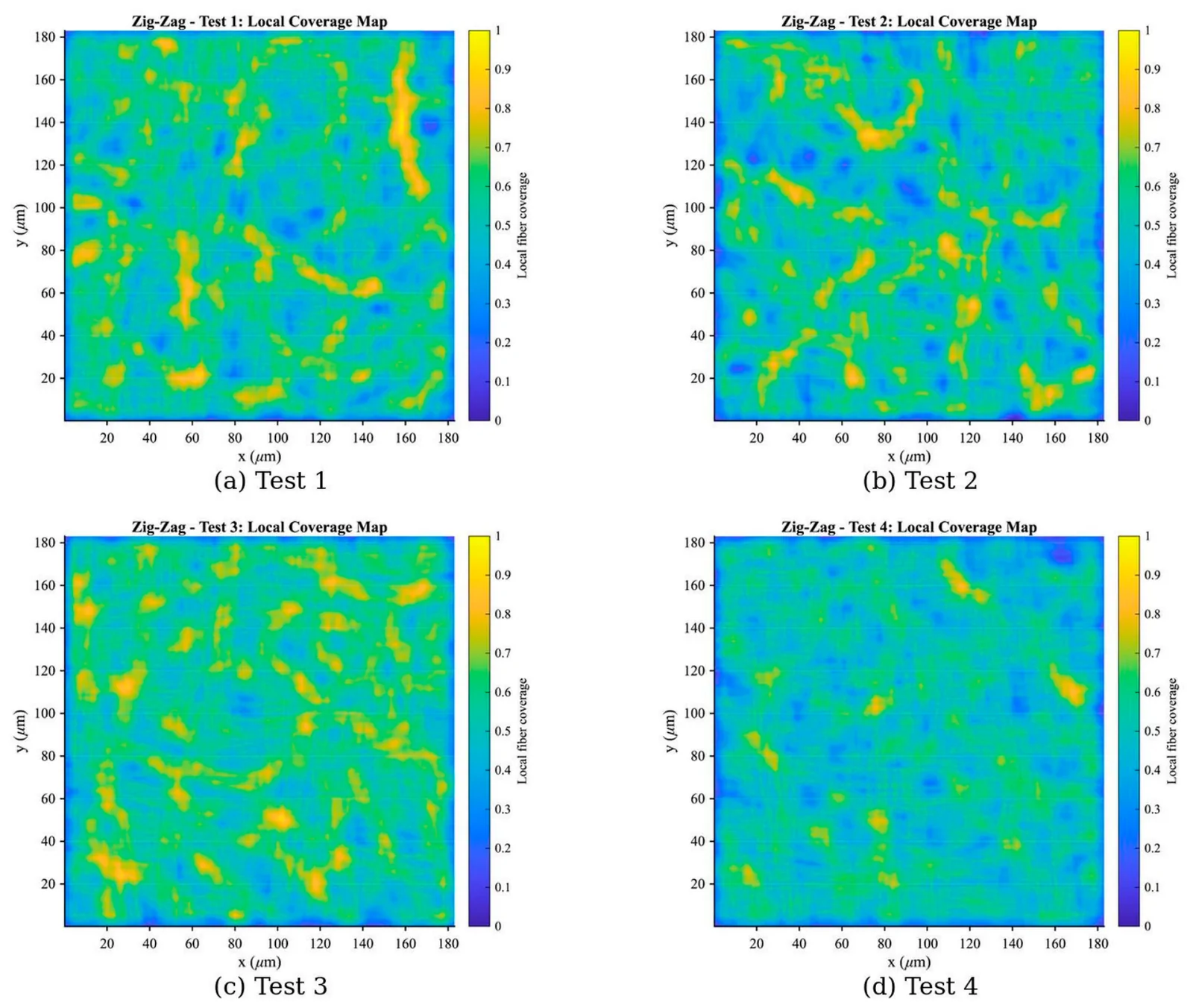
Zig-Zag: local coverage maps for the four independent depositions (T1–T4).

**Figure A50:**
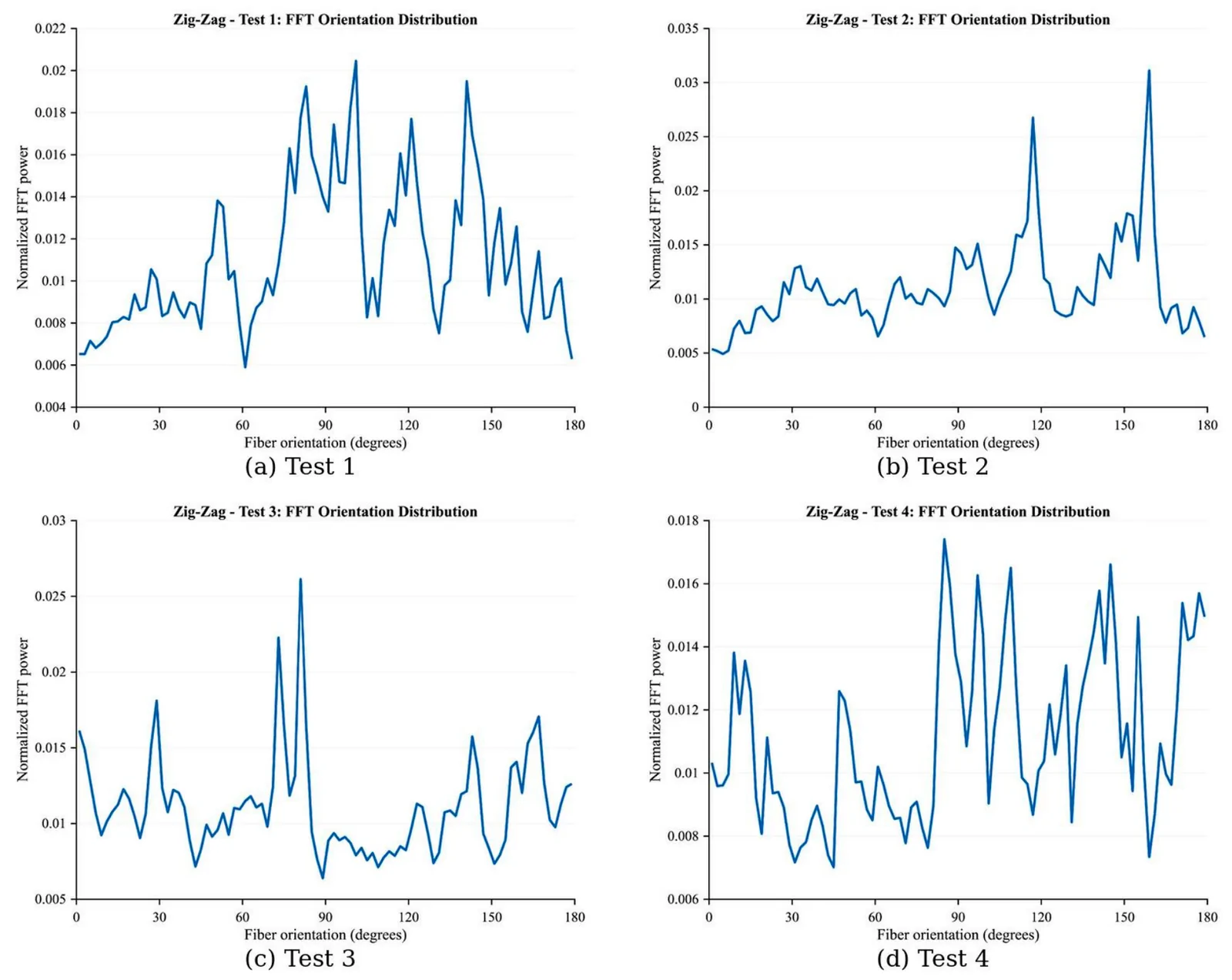
Zig-Zag: FFT-based orientation distributions for the four independent depositions (T1–T4).

**Figure A51:**
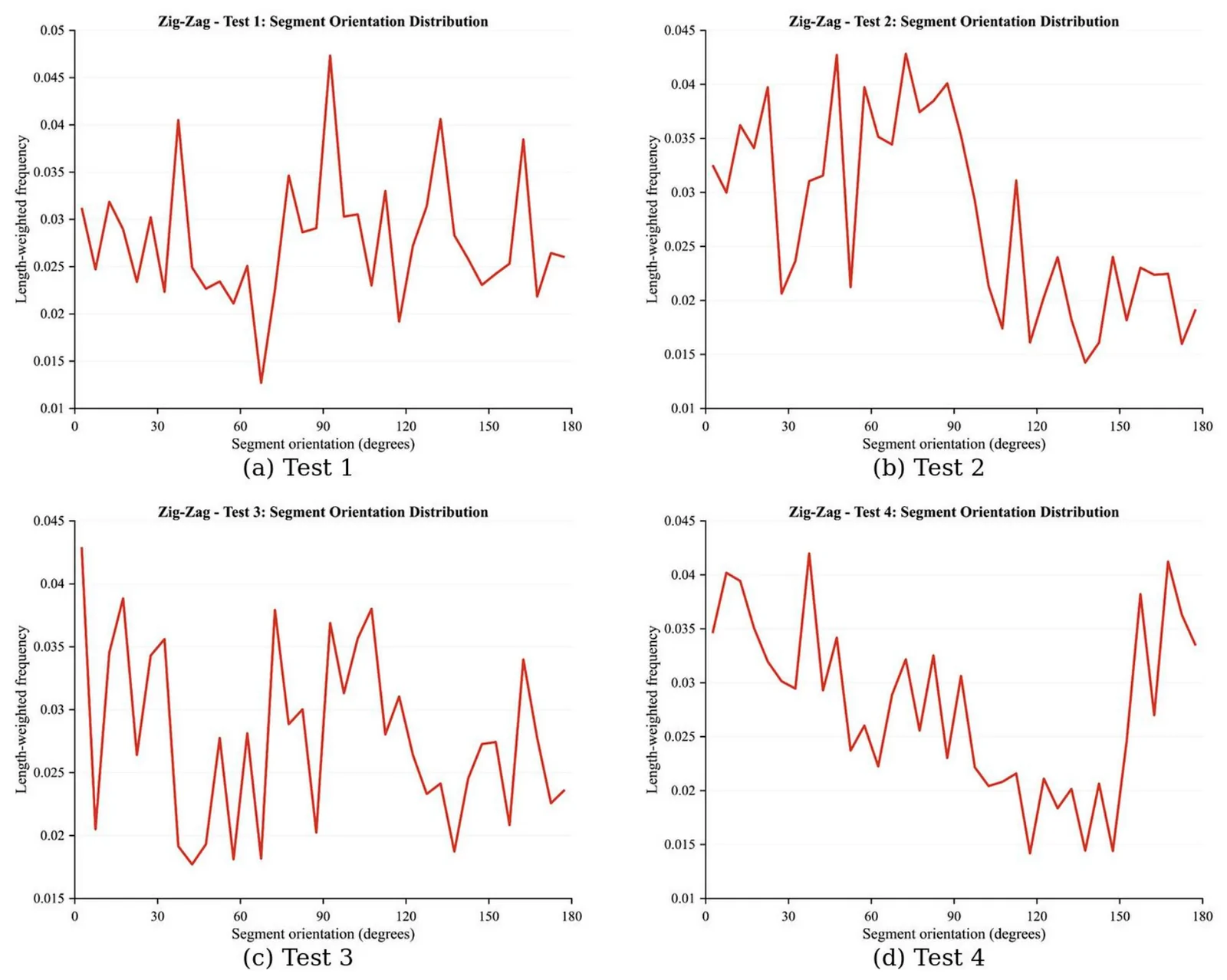
Zig-Zag: skeleton-segment orientation distributions for the four independent depositions (T1–T4).

**Figure A52:**
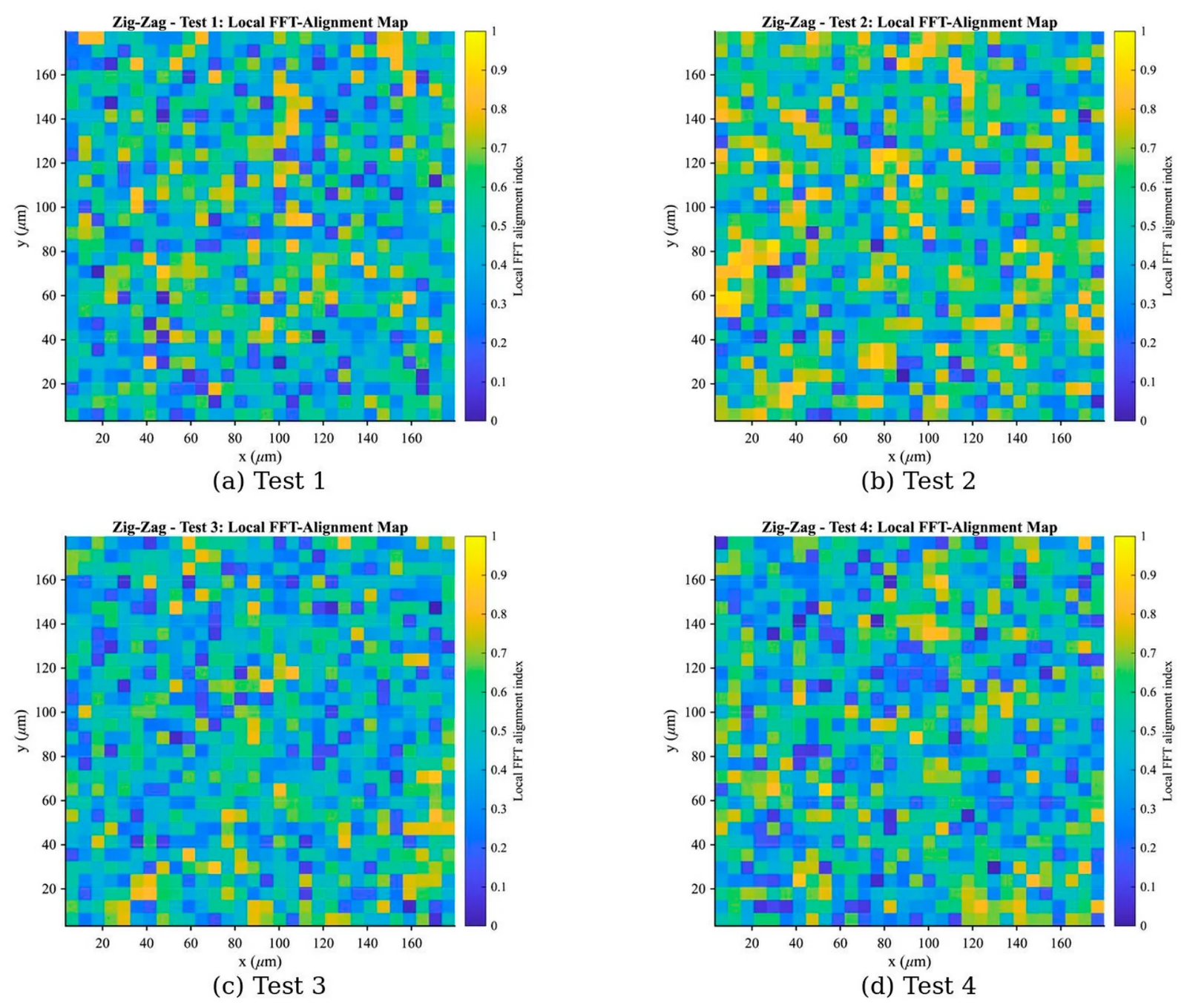
Zig-Zag: local FFT-alignment maps for the four independent depositions (T1–T4).

**Figure A53:**
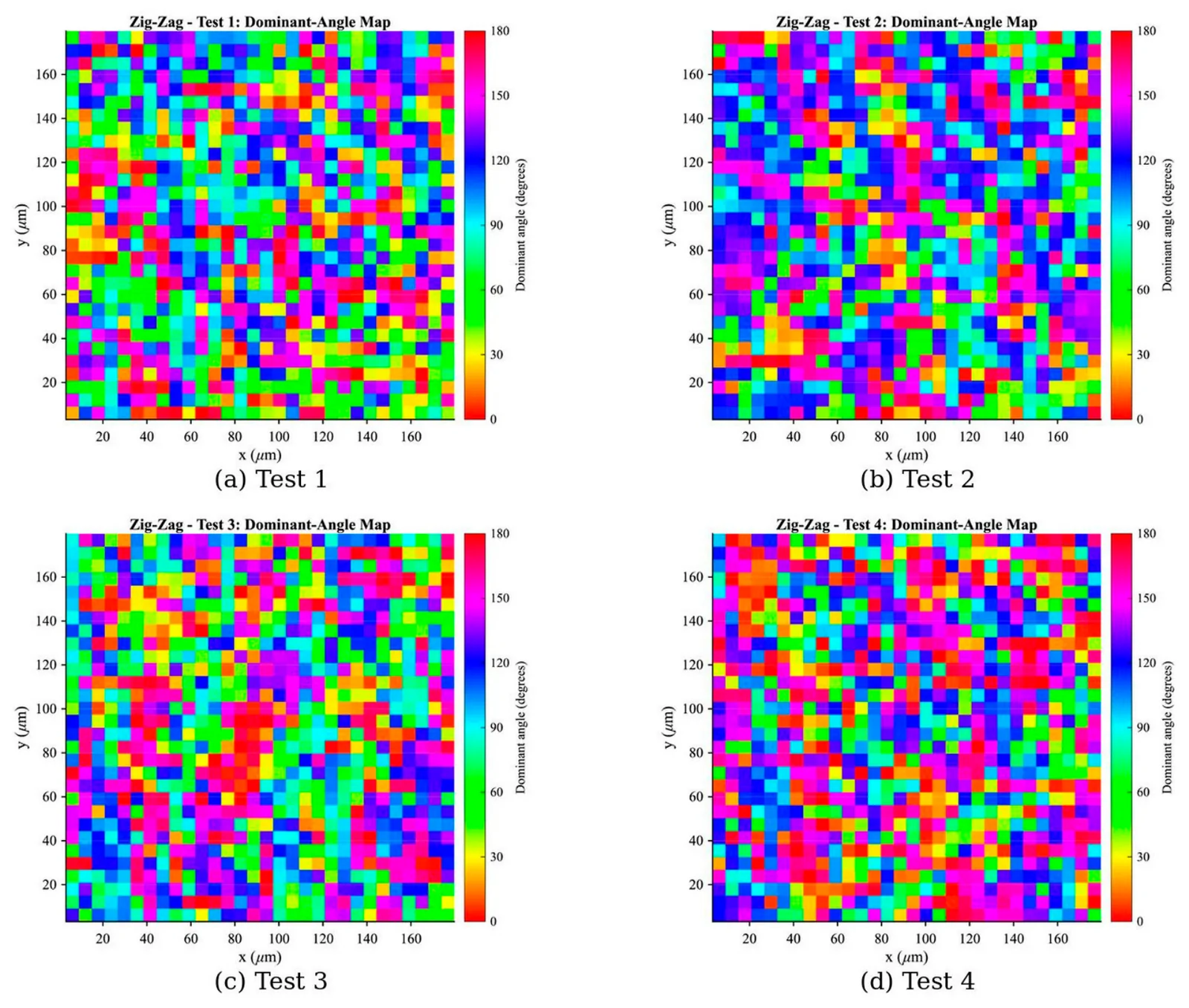
Zig-Zag: dominant-angle maps for the four independent depositions (T1–T4).

## Data Availability

The data supporting the reported results are contained within the article and the associated SEM and tensile-analysis files. MATLAB R2025b, Update 7 (Version 25.2.0.3360999) code used for image processing and workflow files are available from the corresponding author upon reasonable request.

## Abbreviations

Abbreviation	Meaning
PCL	Polycaprolactone
SEM	Scanning electron microscopy
ROI	Region of interest
FFT	Fast Fourier transform
CV	Coefficient of variation
PDI	Polydispersity index
GSM	Grams per square metre/basis weight

## Appendix A

Appendix A provides the complete replicate-resolved SEM image-processing outputs used in the revised quantitative analysis. The four original full-field SEM images for each trajectory are already displayed in Figure 3, Figure 4, Figure 5 and Figure 6 and are intentionally not repeated here. Figure A1 documents automatic scale-bar/ROI detection across all 16 SEM images; Figure A2, Figure A3, Figure A4, Figure A5, Figure A6, Figure A7, Figure A8, Figure A9, Figure A10, Figure A11, Figure A12, Figure A13, Figure A14, Figure A15, Figure A16, Figure A17, Figure A18, Figure A19, Figure A20, Figure A21, Figure A22, Figure A23, Figure A24, Figure A25, Figure A26, Figure A27, Figure A28, Figure A29, Figure A30, Figure A31, Figure A32, Figure A33, Figure A34, Figure A35, Figure A36, Figure A37, Figure A38, Figure A39, Figure A40, Figure A41, Figure A42, Figure A43, Figure A44, Figure A45, Figure A46, Figure A47, Figure A48, Figure A49, Figure A50, Figure A51, Figure A52 and Figure A53 group the remaining outputs by trajectory and analysis type. In every four-panel figure, (a)–(d) correspond to Tests 1–4, which were obtained from four independent deposition runs.

### Appendix A.1. Spiral Condition

The following figures present every replicate-resolved image-processing output for the four independent Spiral depositions that is not already shown in the main-text composite figure. Each grouped figure contains Tests 1–4 exactly once.

### Appendix A.2. Static Condition

The following figures present every replicate-resolved image-processing output for the four independent Static depositions that is not already shown in the main-text composite figure. Each grouped figure contains Tests 1–4 exactly once.

### Appendix A.3. Waves Condition

The following figures present every replicate-resolved image-processing output for the four independent Waves depositions that is not already shown in the main-text composite figure. Each grouped figure contains Tests 1–4 exactly once.

### Appendix A.4. Zig-Zag Condition

The following figures present every replicate-resolved image-processing output for the four independent Zig-Zag depositions that is not already shown in the main-text composite figure. Each grouped figure contains Tests 1–4 exactly once.
